# Supplementary material for: Genome-wide association study identifies risk loci for progressive chronic lymphocytic leukemia
Source: Nat Commun. 2021 Jan 28;12:665. doi: 10.1038/s41467-020-20822-9 (PMC7843618; doi:10.1038/s41467-020-20822-9)
Supplement: Supplementary file 1 — Supplementary Information [file 41467_2020_20822_MOESM1_ESM.pdf]

Genome-wide association study identifies risk loci for progressive chronic lymphocytic leukemia

Wei-Yu Lin[1], Sarah E. Fordham[1], Nicola Sunter[1], Claire Elstob[1], Thahira Rahman[1], Elaine Willmore[1], Colin Shepherd[1], Gordon Strathdee[1], Tryfonia Mainou-Fowler[1], Rachel Piddock[1], Hannah Mearns[1], Timothy Barrow[2], Richard S. Houlston[3], Helen Marr[4], Jonathan Wallis[4], Geoffrey Summerfield[5], Scott Marshall[6], Andrew Pettitt[7], Christopher Pepper[8], Christopher Fegan[9], Francesco Forconi[10], Martin J. S. Dyer[11], Sandrine Jayne[11], April Sellors[11], Anna Schuh[12], Pauline Robbe[12], David Oscier[13], James Bailey[14], Syed Rais[14], Alison Bentley[15], Lynn Cawkwell[16], Paul Evans[17], Peter Hillmen[18], Guy Pratt[19], David J. Allsup[14,15,*,+], James M. Allan[1,*,+]

[1] *Translational and Clinical Research Institute, Newcastle University Centre for Cancer, Faculty of Medical Sciences, Newcastle University, Newcastle upon Tyne, UK.*
[2] *Faculty of Health Sciences & Wellbeing, University of Sunderland, Sunderland, UK.*
[3] *Division of Genetics and Epidemiology, The Institute of Cancer Research, London, UK.*
[4] *Department of Haematology, Freeman Hospital, Newcastle upon Tyne, UK.*
[5] *Queen Elizabeth Hospital, Gateshead, UK.*
[6] *City Hospitals Sunderland NHS Trust, Sunderland, UK.*
[7] *University of Liverpool, Liverpool UK.*
[8] *Brighton and Sussex Medical School, University of Sussex, Brighton, UK.*
[9] *Institute of Cancer and Genetics, School of Medicine, Cardiff, UK.*
[10] *Cancer Sciences Unit, Cancer Research UK and NIHR Experimental Cancer Medicine Centres, University of Southampton, Southampton, United Kingdom.*
[11] *The Ernest and Helen Scott Haematological Research Institute, Leicester Cancer Research Centre, University of Leicester, Leicester, UK.*
[12] *University of Oxford, Oxford, UK.*
[13] *Royal Bournemouth Hospital, Bournemouth, UK.*
[14] *Hull University Teaching Hospital NHS Trust, Hull, UK.*
[15] *Hull York Medical School, Hull, UK.*
[16] *University of Hull, Hull, UK.*
[17] *Haematological Malignancy Diagnostic Service Laboratory, St James's Institute of Oncology, Leeds, UK.*
[18] *Section of Experimental Haematology, Leeds Institute of Medical Research at St James's, University of Leeds, Leeds, UK.*
[19] *University of Birmingham, Birmingham, UK.*
[*] *Joint contribution.*
[+] *Corresponding author: **James M. Allan**. Email: james.allan@newcastle.ac.uk. Telephone: +44 (0) 191 208 4435, Northern Institute for Cancer Research, Newcastle University, Newcastle upon Tyne, United Kingdom, NE2 4HH; **David Allsup**. Email hycda1@hyms.ac.uk. Telephone +44(0) 1482 461294, Hull York Medical School, University of Hull, Hull United Kingdom, HU6 7RX.*

List of Figures

1 Kaplan-Meier plot of time to first treatment for CLL patients stratified by Binet stage of disease . . . 1
2 Kaplan-Meier plot of time to first treatment for CLL patients stratified by IGHV status 2
3 Kaplan-Meier plot of time to first treatment for CLL patients stratified by CD38 status 3
4 Kaplan-Meier plot of time to first treatment for CLL patients stratified by $\beta2$ Microglobulin . . . 4
5 Kaplan-Meier plot of time to first treatment for CLL patients stratified by TP53 status 5
6 GWAS QC and analysis flowchart . . . 6
7 Principal component analysis plot of ethnicity structure in CLL GWAS and 1000 genomes population panels . . . 7
8 Sanger validation genotyping for rs736456 and rs3778076 . . . 8
9 Quantile-Quantile plot of fixed-effect meta-analysis for time to first treatment . . . 9
10 Regional association plots for association analysis conditioning on the top variant . . 10
11 Associations between post-treatment survival and risk variants for progressive CLL . 11
12 Time to first treatment for CLL patients stratified by Binet stage of disease and SNP genotypes . . . 12
13 Time to first treatment for CLL patients stratified by IGHV status and SNP genotypes 13
14 Time to first treatment for CLL patients stratified by CD38 status and SNP genotypes 14
15 Time to first treatment for CLL patients stratified by $\beta2$ Microglobulin and SNP genotypes . . . 15
16 Time to first treatment for CLL patients stratified by TP53 status and SNP genotypes 16
17 Survival curves in low-risk CLLs by rs736456 (a) rs3778076 (b) and risk allele groups of rs736456 and rs3778076 (c) . . . 18
18 Regional association plot of TTFT for known CLL etiological risk variant rs34676223 19
19 Regional association plot of TTFT for known CLL etiological risk variant rs41271473 20
20 Regional association plot of TTFT for known CLL etiological risk variant rs3770745 . 21
21 Regional association plot of TTFT for known CLL etiological risk variant rs13401811 22
22 Regional association plot of TTFT for known CLL etiological risk variant rs17483466 23
23 Regional association plot of TTFT for known CLL etiological risk variant rs9308731 . 24
24 Regional association plot of TTFT for known CLL etiological risk variant rs3769825 . 25
25 Regional association plot of TTFT for known CLL etiological risk variant rs13397985 26
26 Regional association plot of TTFT for known CLL etiological risk variant rs757978 . . 27
27 Regional association plot of TTFT for known CLL etiological risk variant rs9880772 . 28
28 Regional association plot of TTFT for known CLL etiological risk variant rs1274963 . 29
29 Regional association plot of TTFT for known CLL etiological risk variant rs10936599 30
30 Regional association plot of TTFT for known CLL etiological risk variant rs10028805 31
31 Regional association plot of TTFT for known CLL etiological risk variant rs898518 . . 32
32 Regional association plot of TTFT for known CLL etiological risk variant rs57214277 33
33 Regional association plot of TTFT for known CLL etiological risk variant rs31490 . . 34
34 Regional association plot of TTFT for known CLL etiological risk variant rs872071 . . 35
35 Regional association plot of TTFT for known CLL etiological risk variant rs73718779 36
36 Regional association plot of TTFT for known CLL etiological risk variant rs674313 . . 37
37 Regional association plot of TTFT for known CLL etiological risk variant rs210142 . . 38
38 Regional association plot of TTFT for known CLL etiological risk variant rs3800461 . 39
39 Regional association plot of TTFT for known CLL etiological risk variant rs2236256 . 40
40 Regional association plot of TTFT for known CLL etiological risk variant rs17246404 41
41 Regional association plot of TTFT for known CLL etiological risk variant rs2456449 . 42
42 Regional association plot of TTFT for known CLL etiological risk variant rs1679013 . 43
43 Regional association plot of TTFT for known CLL etiological risk variant rs4406737 . 44
44 Regional association plot of TTFT for known CLL etiological risk variant rs61904987 45

45 Regional association plot of TTFT for known CLL etiological risk variant rs735665 . . 46
46 Regional association plot of TTFT for known CLL etiological risk variant rs10735079 47
47 Regional association plot of TTFT for known CLL etiological risk variant rs8024033 . 48
48 Regional association plot of TTFT for known CLL etiological risk variant rs7169431 . 49
49 Regional association plot of TTFT for known CLL etiological risk variant rs7176508 . 50
50 Regional association plot of TTFT for known CLL etiological risk variant rs783540 . . 51
51 Regional association plot of TTFT for known CLL etiological risk variant rs391525 . . 52
52 Regional association plot of TTFT for known CLL etiological risk variant rs305065 . . 53
53 Regional association plot of TTFT for known CLL etiological risk variant rs305061 . . 54
54 Regional association plot of TTFT for known CLL etiological risk variant rs1036935 . 55
55 Regional association plot of TTFT for known CLL etiological risk variant rs4368253 . 56
56 Regional association plot of TTFT for known CLL etiological risk variant rs4987855 . 57
57 Regional association plot of TTFT for known CLL etiological risk variant rs7254272 . 58
58 Regional association plot of TTFT for known CLL etiological risk variant rs11083846 59
59 Regional association plot of TTFT for known CLL etiological risk variant rs140522 . . 60
60 Regional and forest plots of rs4752676 61
61 Regional and forest plots of rs736457 62
62 Regional and forest plots of rs11757517 63

List of Tables

1 Demographic and clinical characteristics of chronic lymphocytic leukemia (CLL) cases 64
2 eQTL results for rs736456 65
3 eQTL results for rs3778076 66
4 eQTL results for rs3800461 67

Supplementary Figures

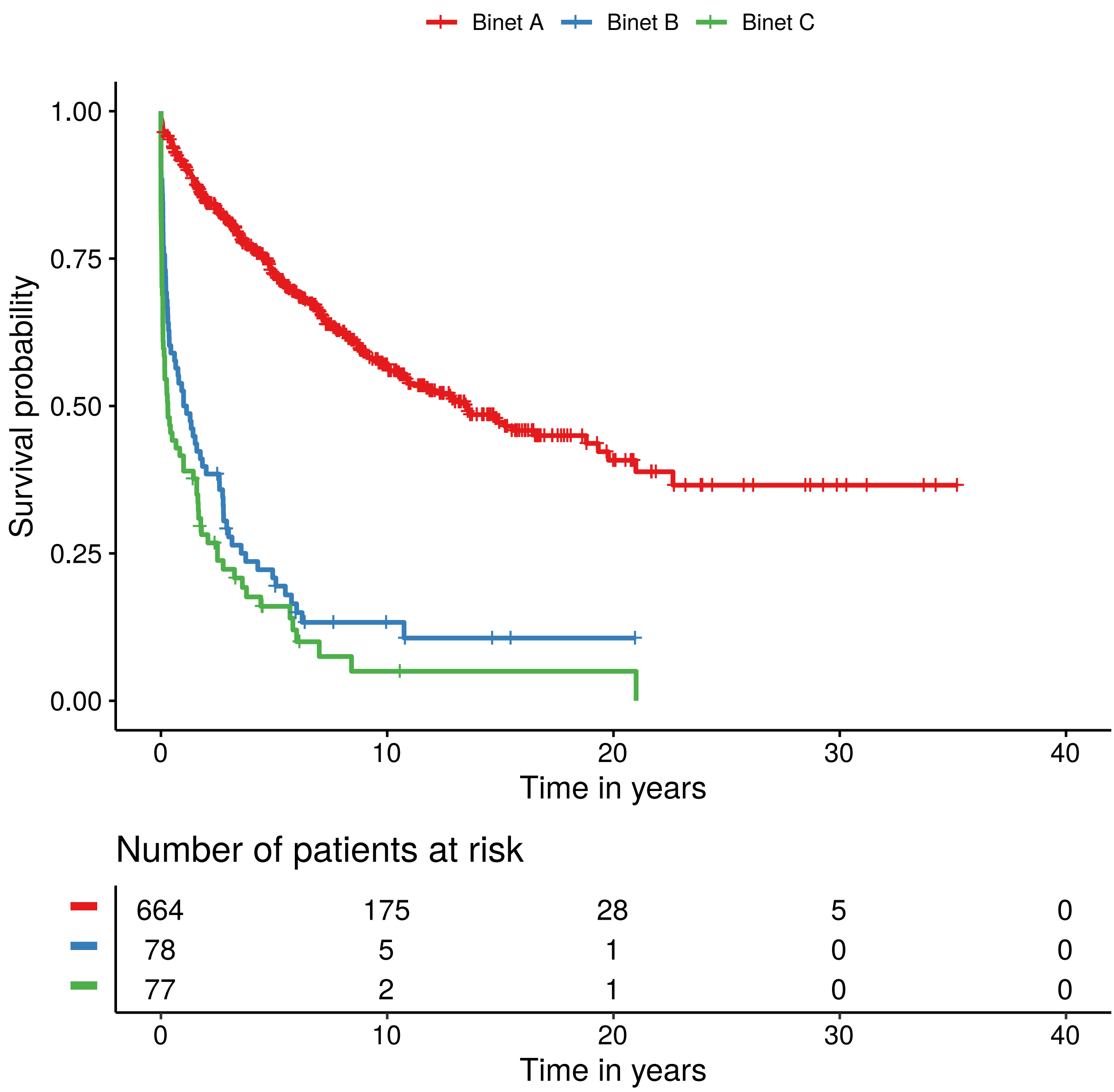

Supplementary Figure 1: **Kaplan-Meier plot showing time to first treatment (TTFT) for CLL patients stratified by Binet stage of disease**. TTFT is defined as the time from diagnosis of CLL to treatment or last follow-up without treatment. Patients censored at last follow-up are indicated by a cross. The number of patients in each group at each timepoint are indicated in the table.

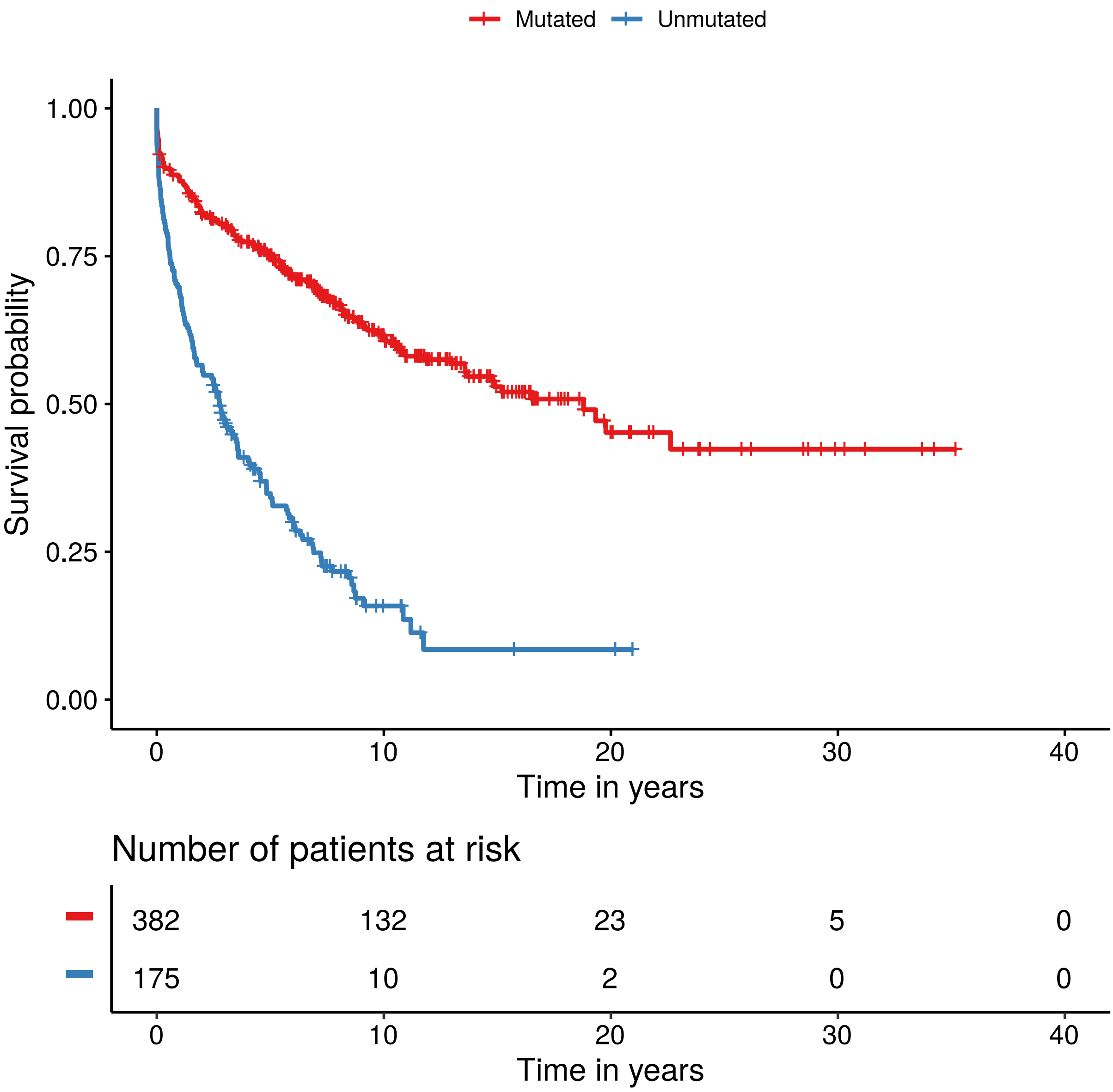

Supplementary Figure 2: **Kaplan-Meier plot showing time to first treatment (TTFT) for CLL patients stratified by IGHV status**. TTFT is defined as the time from diagnosis of CLL to treatment or last follow-up without treatment. Patients censored at last follow-up are indicated by a cross. The number of patients in each group at each timepoint are indicated in the table.

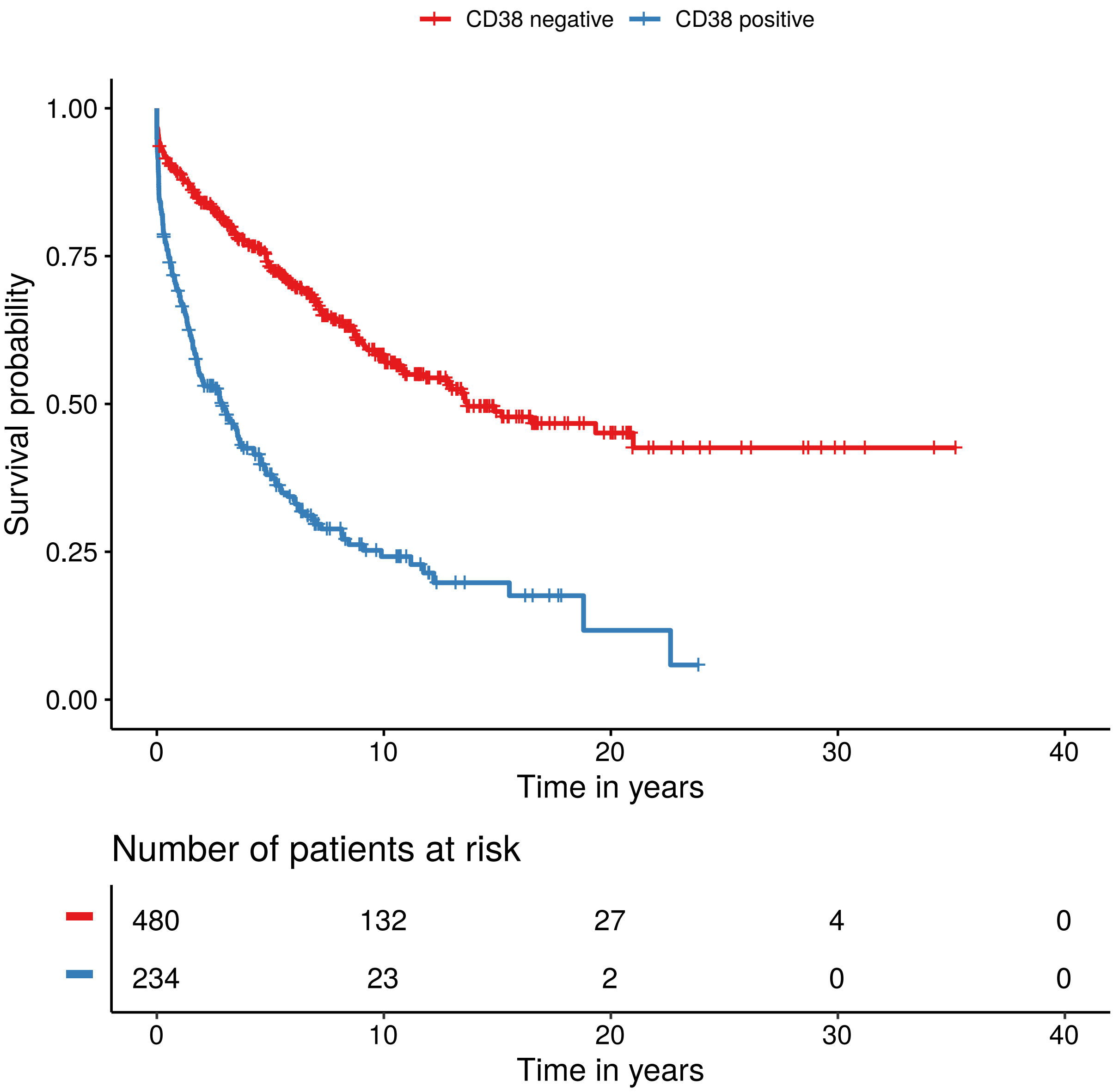

Supplementary Figure 3: **Kaplan-Meier plot showing time to first treatment (TTFT) for CLL patients stratified by CD38 status**. TTFT is defined as the time from diagnosis of CLL to treatment or last follow-up without treatment. Patients censored at last follow-up are indicated by a cross. The number of patients in each group at each timepoint are indicated in the table.

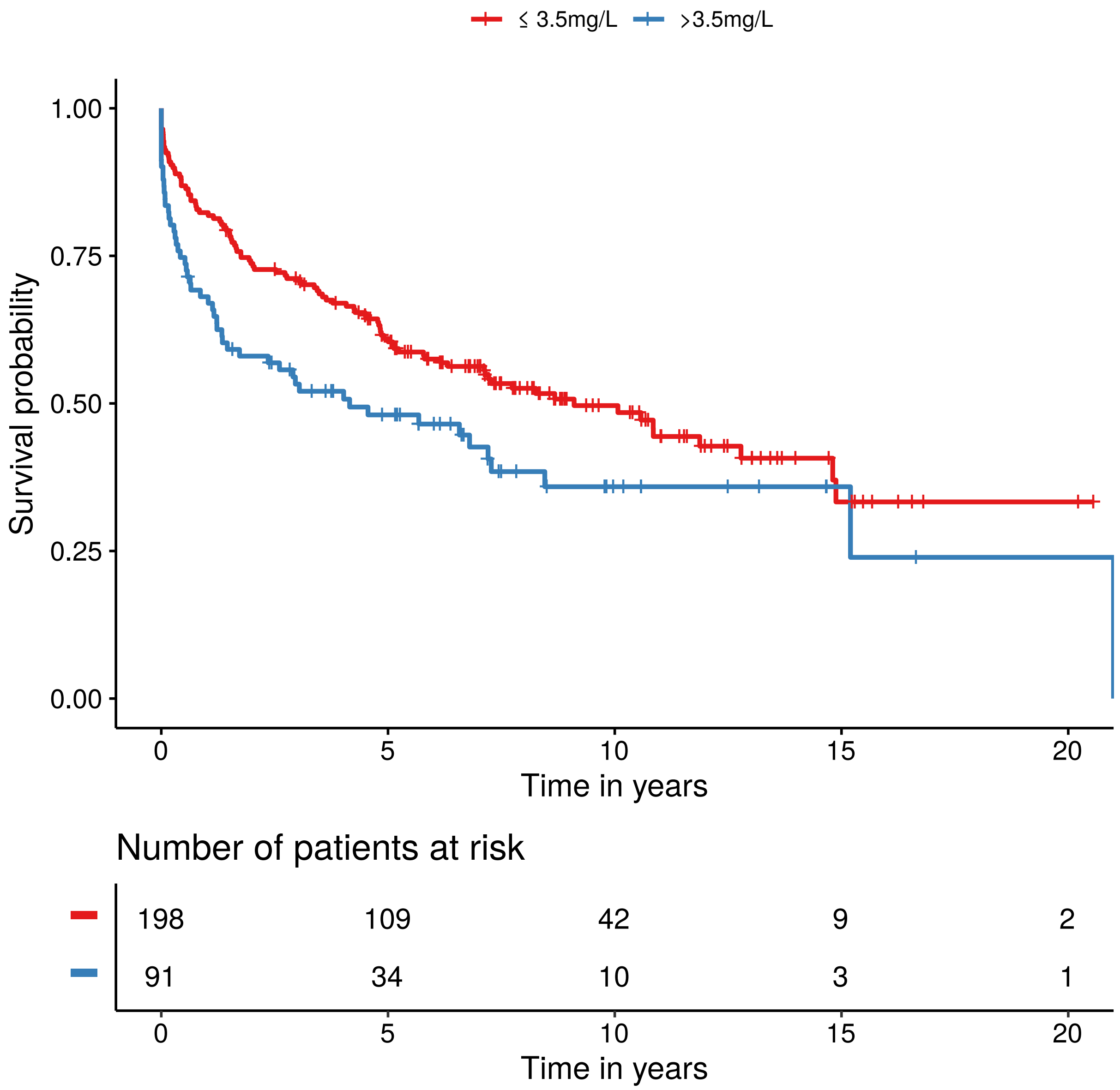

Supplementary Figure 4: **Kaplan-Meier plot showing time to first treatment (TTFT) for CLL patients stratified by $\beta 2$ Microglobulin**. TTFT is defined as the time from diagnosis of CLL to treatment or last follow-up without treatment. Patients censored at last follow-up are indicated by a cross. The number of patients in each group at each timepoint are indicated in the table. High serum $\beta 2$ microglobulin is defined as > 3.5 mg/L.

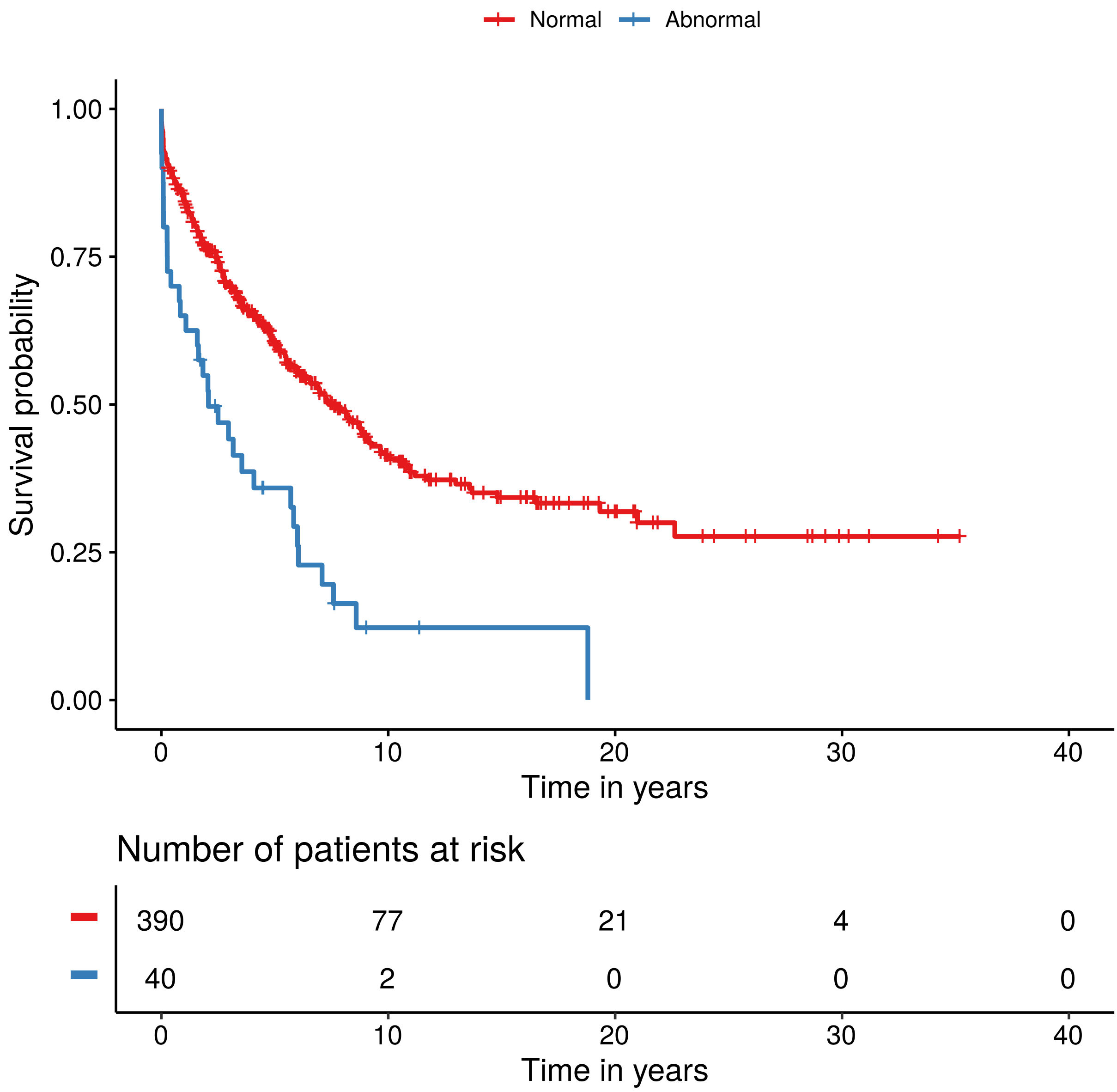

Supplementary Figure 5: **Kaplan-Meier plot showing time to first treatment (TTFT) for CLL patients stratified by TP53 status**. TTFT is defined as the time from diagnosis of CLL to treatment or last follow-up without treatment. Patients censored at last follow-up are indicated by a cross. The number of patients in each group at each timepoint are indicated in the table. Abnormal is defined as a 17p deletion by FISH or a TP53 mutation by Sanger sequencing. It should be noted that TP53 status is not routinely determined in patients with early stage CLL. As such, the cohort tested for TP53 has an over-representation of patients with progressive CLL.

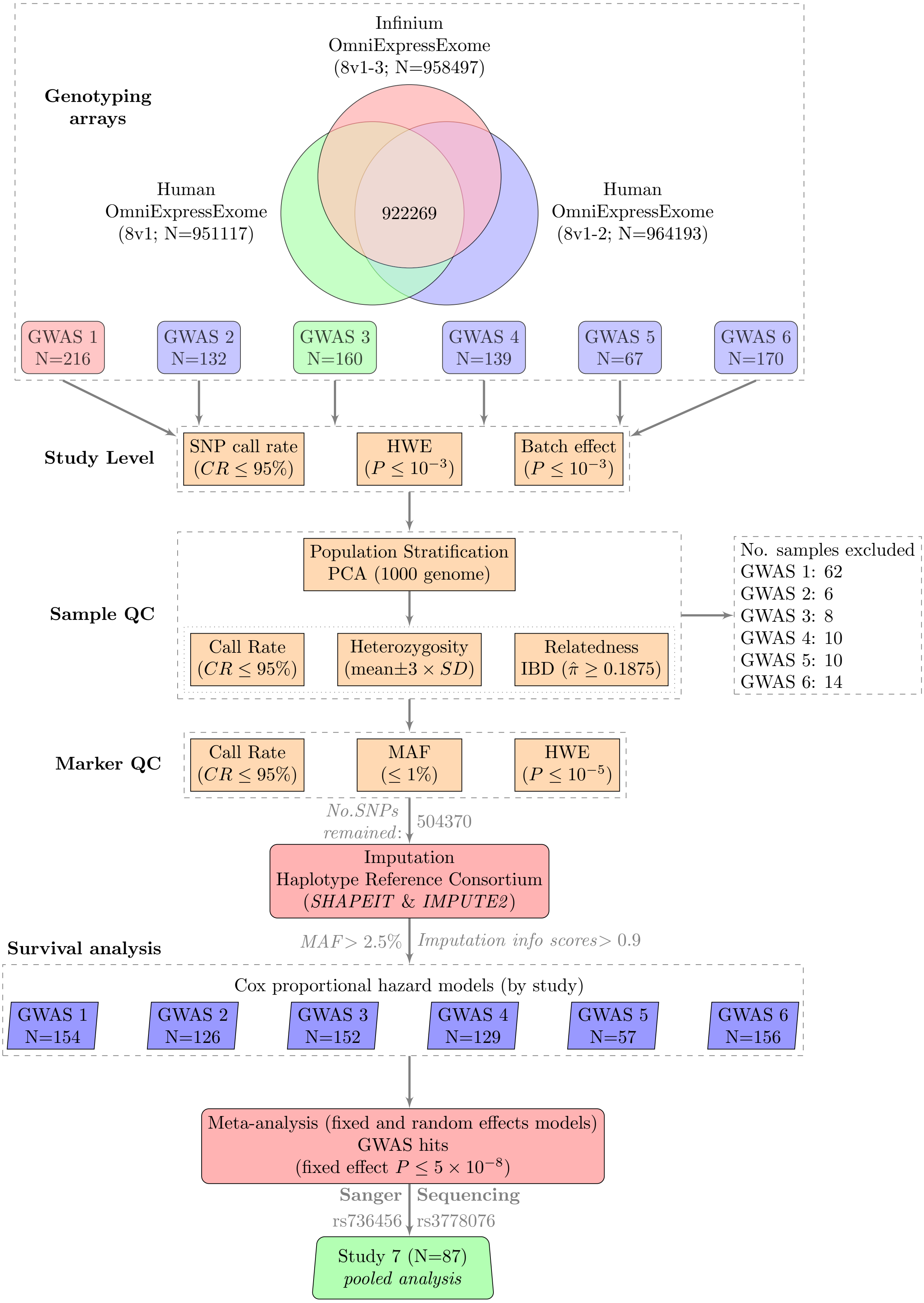

Supplementary Figure 6: **Details of quality control filters applied to each CLL GWAS and data analysis workflow.** SNPs with a call rate $< 95\%$, significant heterogeneity between studies (batch effect $P \leq 10^{-3}$) or showing significant deviation from Hardy-Weinberg equilibrium ($P \leq 10^{-3}$) were excluded. Samples were excluded due to low call rate ($< 95\%$), ancestry (principle components analysis), relatedness ($\pi \geq 0.1875$) or heterozygosity (mean $\pm 3\times$ SD). Imputed SNPs with information score < 0.9 and MAF < 0.025 were excluded. For each study, allelic dosage was estimated for the minor allele at each variant position and included in a cox proportional hazard model to estimate hazard ratio (HR) and 95% confidence interval (CI). Study-specific single nucleotide polymorphism (SNP) effects were combined using an inverse-variance-weighted method (fixed effects model) and the DerSimonian-Laird approach (random effects model). SNPs with fixed-effect P values of $\leq 5 \times 10^{-8}$ were deemed significant at genome-wide level.

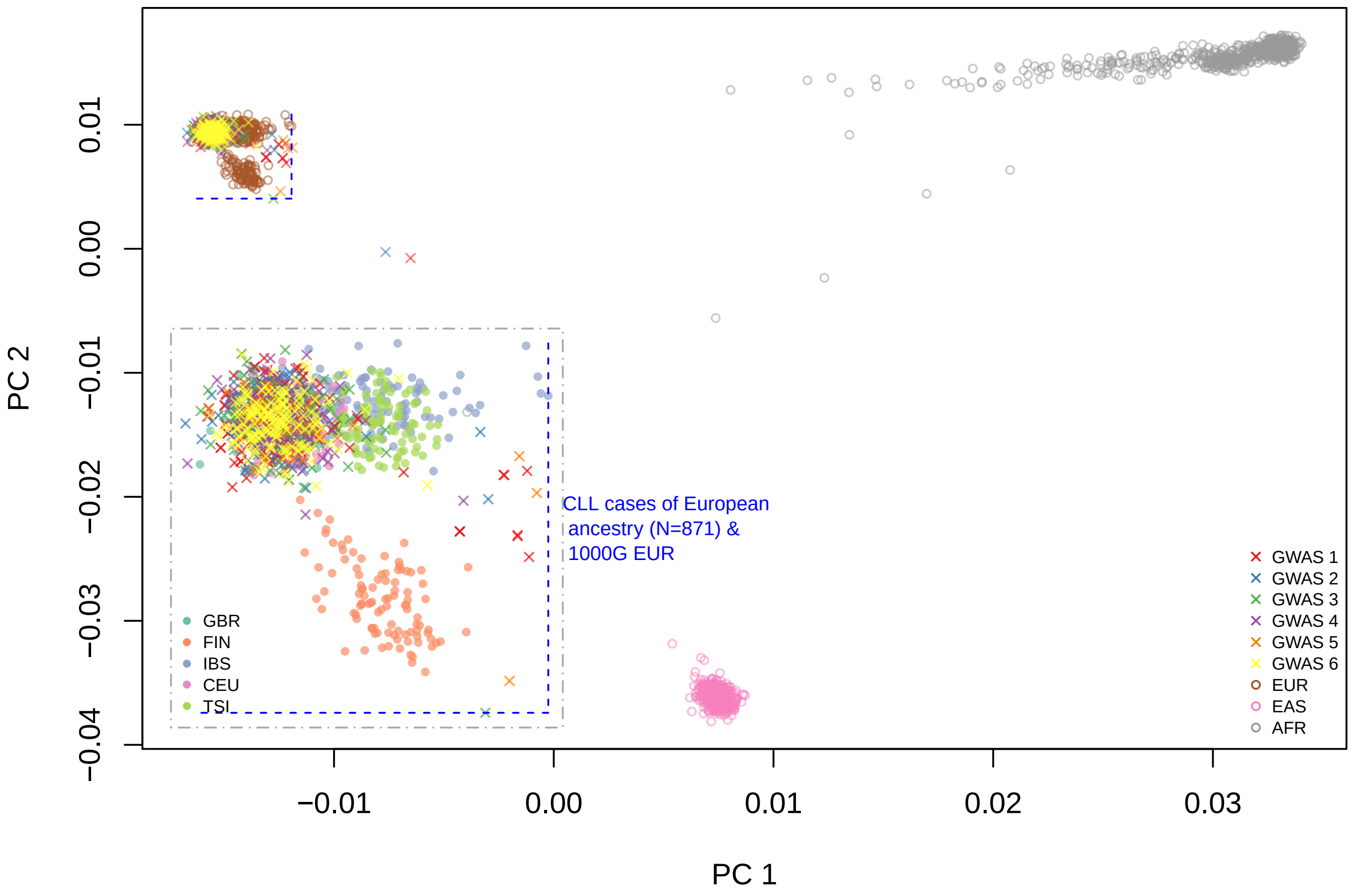

Supplementary Figure 7: **Principal component analysis (PCA) plot of ethnicity structure in CLL GWAS and 1000 genomes population panels.** The first two principal components of the analysis are plotted for CLL cases recruited to this study (crosses). 1000 genomes European (EUR), East Asian (EAS) and African (AFR) individuals (open circles) are plotted in brown, pink and gray, respectively. CLL cases of European ancestry included in subsequent analysis (top-left corner) are shown in the inset together with 1000G EUR sub-populations (closed circles) comprising CEU (Utah Residents (CEPH) with Northern and Western European Ancestry; pink), GBR (British in England and Scotland; dark green), FIN (Finnish in Finland; orange), IBS (Iberian Population in Spain; blue), and TSI (Toscani in Italia; light green). PC1, principal component 1; PC2, principal component 2.

a

rs Identifier	Primer sequences	PCR conditions
rs736456	F 5' CTGTTTGAGGCAGGCTTCTC 3'<br>R 5' GAGCCCTTCCCTGAAAACCTC 3'	25 µL reactions included: 9 µL H_2O, 12.5 µL DreamTaq Green PCR Master Mix (2X), 1.25 µL of each forward and reverse primers (10 µM) and 50 ng DNA (1 µL).<br>Cycling conditions: 2 minutes at 95 °C followed by 35 cycles (25 seconds at 95 °C, 35 seconds at 60 °C and 45 seconds at 72 °C). Final step of 5 minutes at 72 °C with 4 °C hold.
rs3778076	F 5' CTACTTTCCCCGATGCCTGG 3'<br>R 5' ATGTCCTGGGGTTTCAGTGC 3'	

b

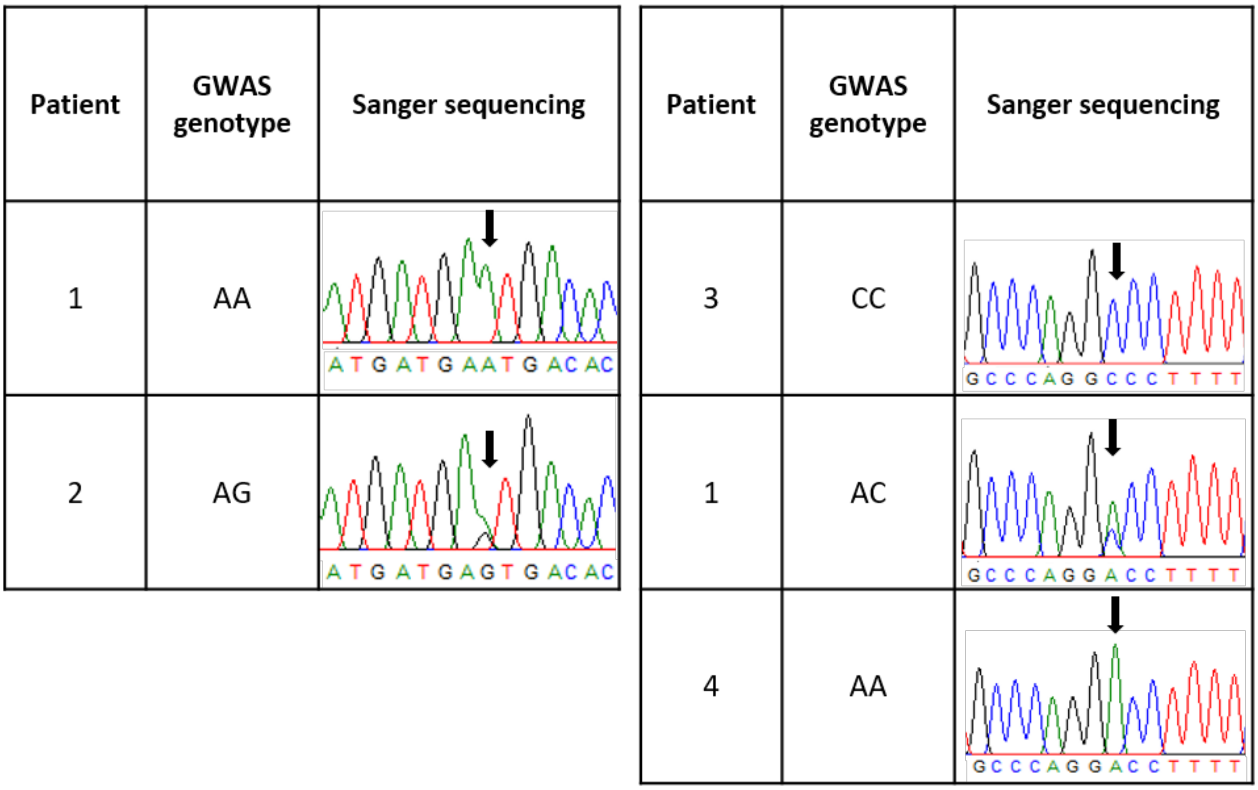

c

rs Identifier	Region	Successful reads	Concordance
rs736456	10q26.13	89/100	89/89 (100%)
rs3778076	6p	96/100	96/96 (100%)

Supplementary Figure 8: **Sanger validation genotyping for rs736456 and rs3778076.** Primer sequences and PCR conditions for validation of rs736456 and rs3778076 (a). Representative genotype results for rs736456 and rs3778076 (b). Concordance between GWAS genotyping and Sanger validation genotyping for rs736456 and rs3778076 (c).

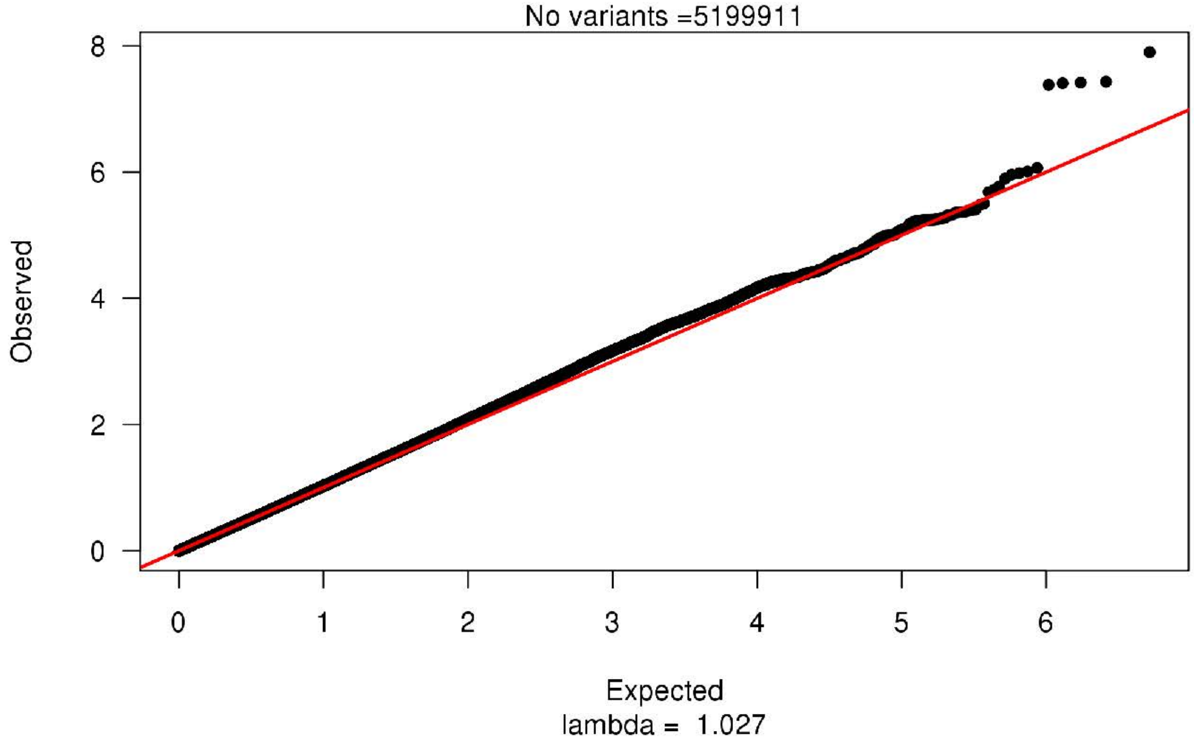

Supplementary Figure 9: **Quantile-Quantile plot of fixed-effect meta-analysis for time to first treatment (TTFT).** For each study, allelic dosage was estimated for the minor allele at each variant position and included in a cox proportional hazard model to estimate hazard ratio (HR) and 95% confidence interval (CI). Variants were included in the meta-analysis if they had results from all six studies. Study-specific single nucleotide polymorphism (SNP) effects were combined in a fixed effect model using an inverse-variance-weighted method. Expected (under the null hypothesis of no association) and observed distributions of -$\log_{10}(P)$ values are shown on the x-axis and y-axis, respectively. The red line corresponds to y = x. Inflation lambda (λ_{GC}= 1.027) is the observed median χ^2 test statistic divided by the median expected χ^2 test statistic under the null hypothesis.

a

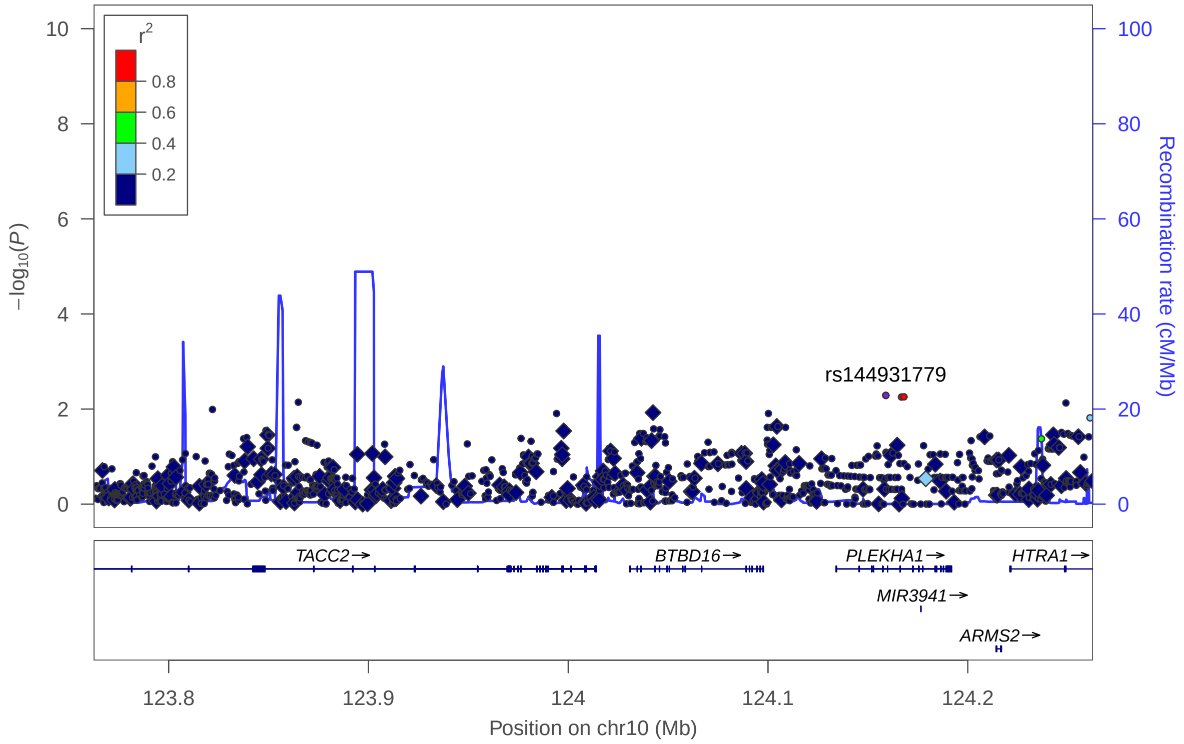

b

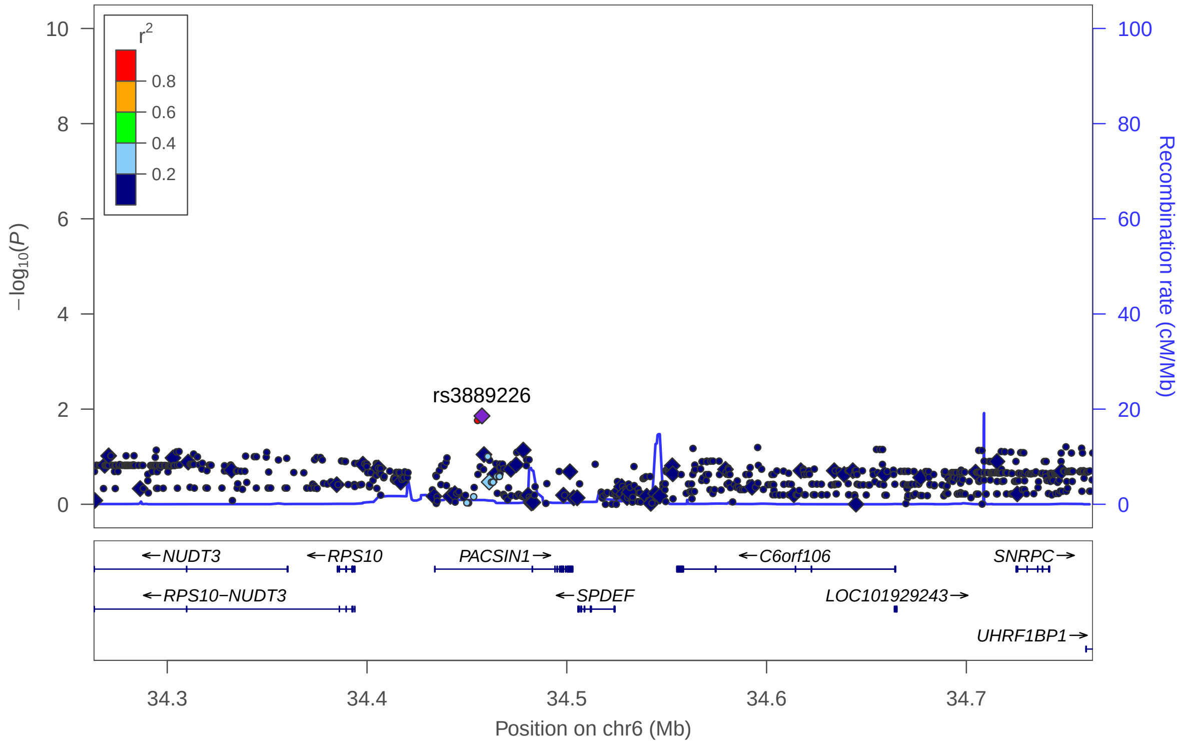

Supplementary Figure 10: **Regional association and linkage disequilibrium plots for association analysis conditioning on the top variant at each susceptibility locus for progressive CLL.** Regional association plots of time to first time treatment (TTFT) survival associations for the chromosome 10 (a) and chromosome 6 (b) loci conditioning on rs736456 and rs3778076, respectively. For each study, Cox proportional hazard models were used to estimate the conditional results for each variant at the region by including the top variant. The resulting effect sizes were combined using an inverse-variance-weighted approach. All statistical tests were two-sided. SNP coordinates based on genomic build b37/hg19 are shown on the x-axis and -$\log_{10}$ (P values) on the y-axis. Genotyped and imputed SNPs are represented by diamonds and circles, respectively. SNPs are coloured according to their linkage disequilibrium (pairwise r^2) with the lead SNP (annotated) based on the 1000 Genomes European panel. Reference genes in the region are shown in the lower panel, with arrows indicating transcript direction, dense blocks representing exons and horizontal lines representing introns.

a

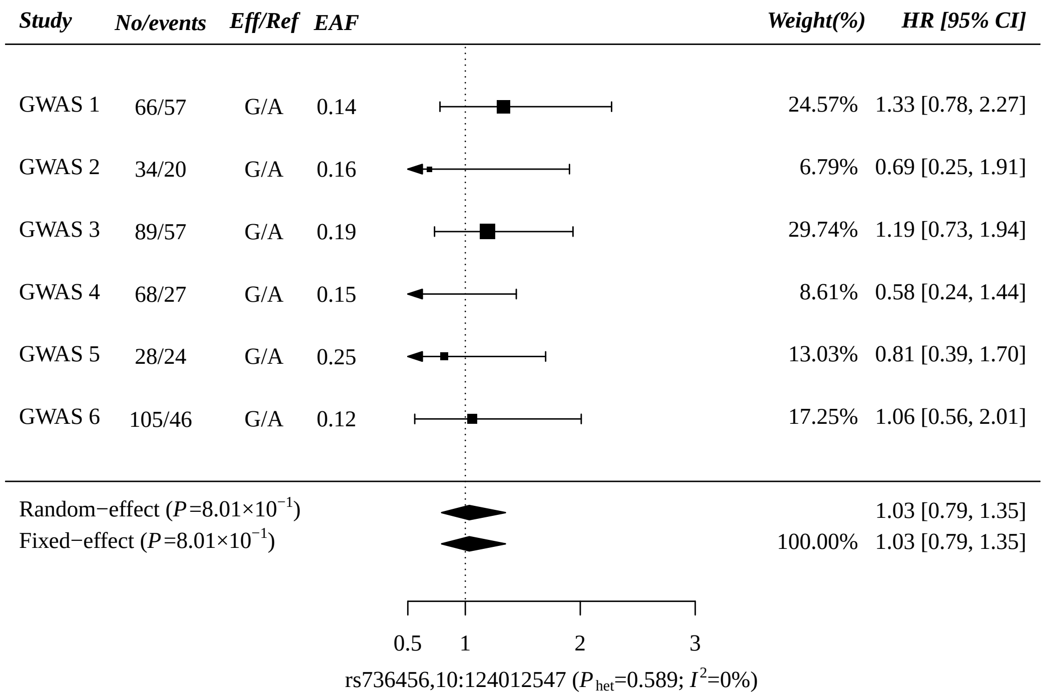

b

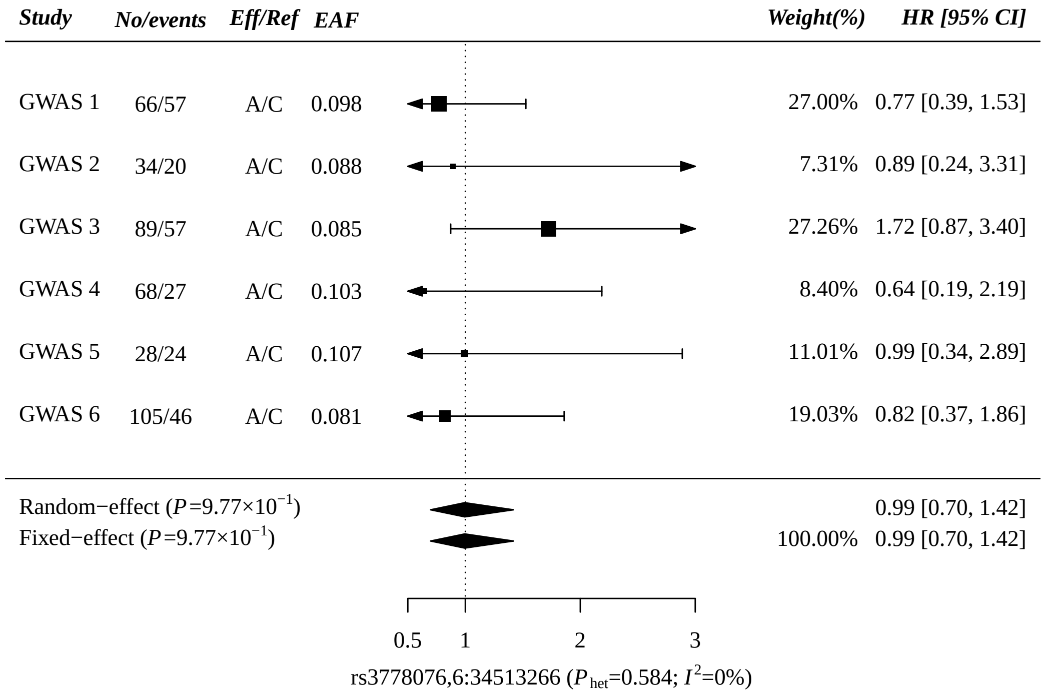

Supplementary Figure 11: **Forest plots showing associations between post-treatment survival and risk variants for progressive CLL.** Forest plots for rs736456 (a) and rs3778076 (b) and their age-adjusted association with post-treatment survival stratified by GWAS. Post-treatment survival is defined as the time from first treatment for CLL-related symptoms to death or last follow-up. No/events: Number of CLL patients/Number of patients receiving treatment; Eff/Ref: effect/reference allele; EAF: effect allele frequency; HR: hazard ratio; CI: confidence interval; Squares denote the per-allele HR, with size proportional to the weight of the study. Pooled HRs derived from both the fixed and random-effects models are indicated by diamonds with their corresponding meta P values shown in the left parentheses. X-axis label formats include reference sequence (rs) identifier and chromosome:position (b37). P values for Cochran's Q test (P_{het}) and I^2 for heterogeneity are shown in parentheses. All statistical tests were two-sided.

a

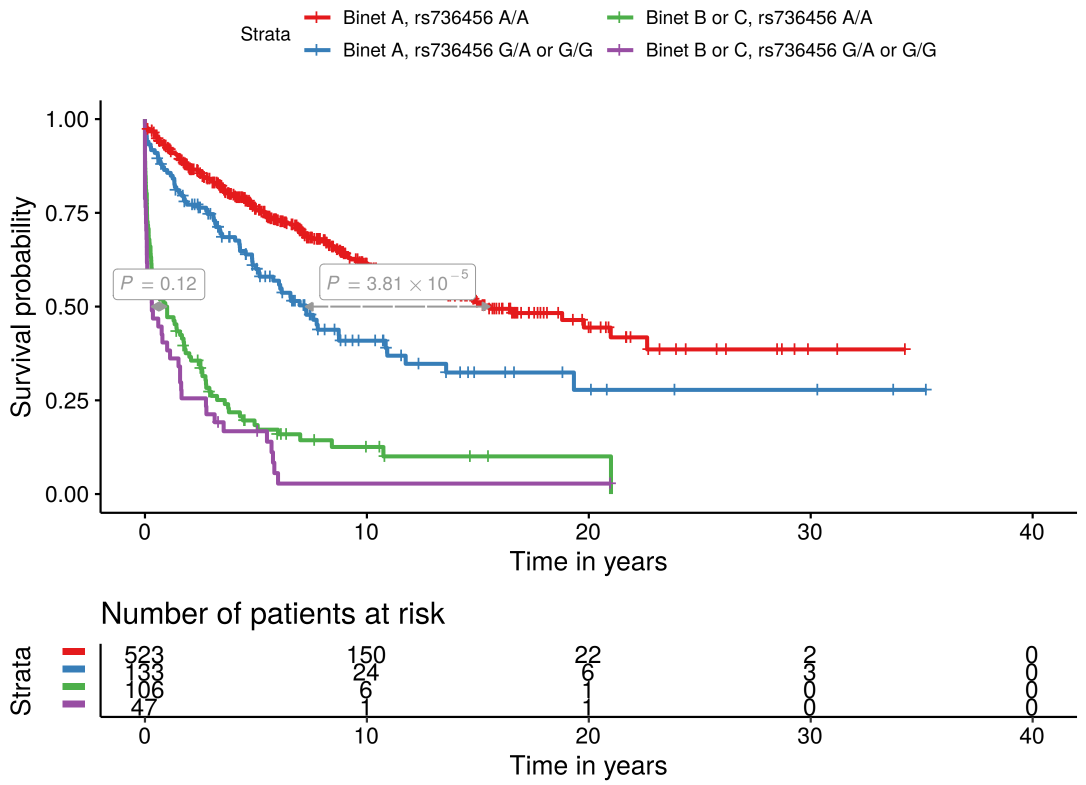

b

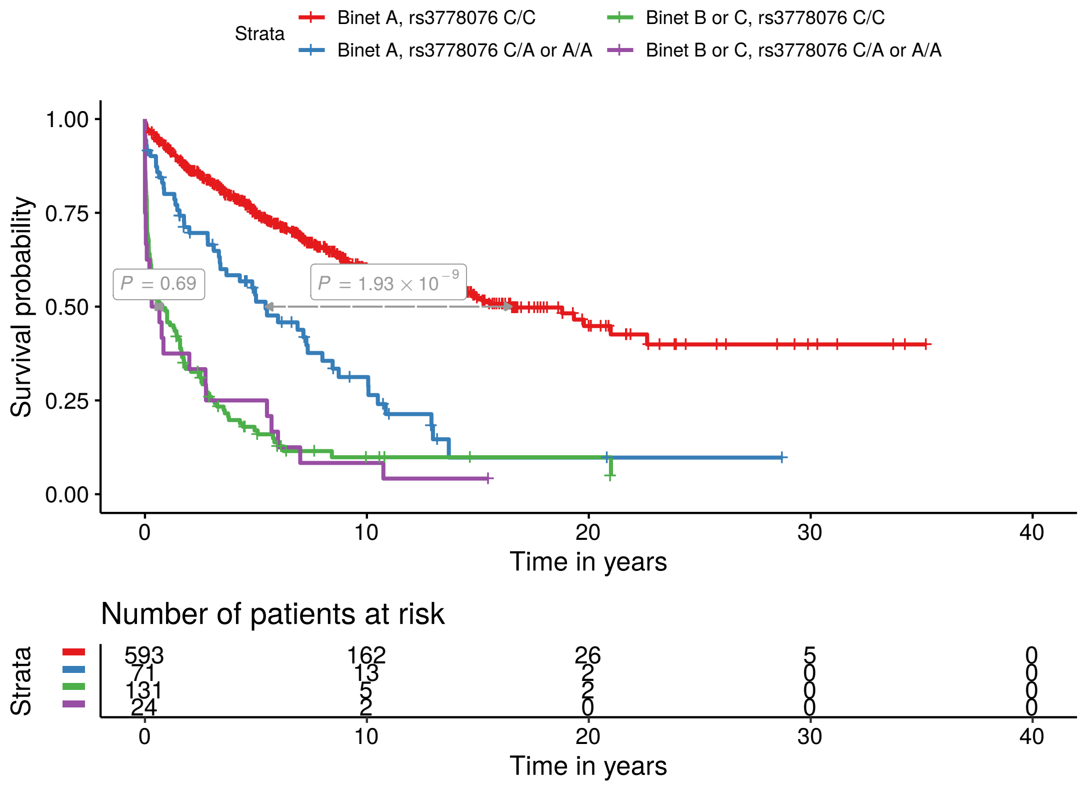

Supplementary Figure 12: **Kaplan-Meier plot showing time to first treatment (TTFT) for CLL patients stratified by Binet stage of disease and SNP genotype for rs736456 (a) and rs3778076 (b).** P values are obtained from pairwise log-rank tests for survival curves, with false discovery rate (FDR) corrections for multiple testing. For simplicity, p values are only shown for the comparisions of SNP genotypes within the same prognostic factor stratum. TTFT is defined as the time from diagnosis of CLL to treatment or last follow-up without treatment. Patients censored at last follow-up are indicated by a cross. The number of patients in each group at each timepoint are indicated in the table. All statistical tests were two-sided.

a

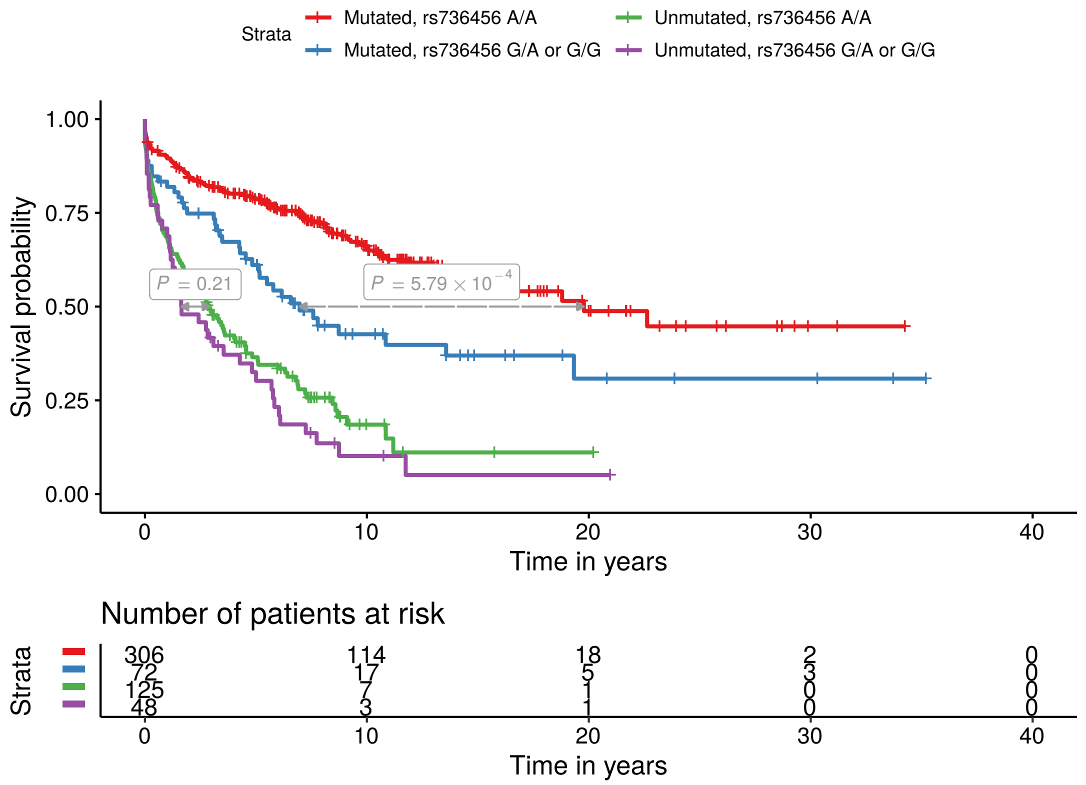

b

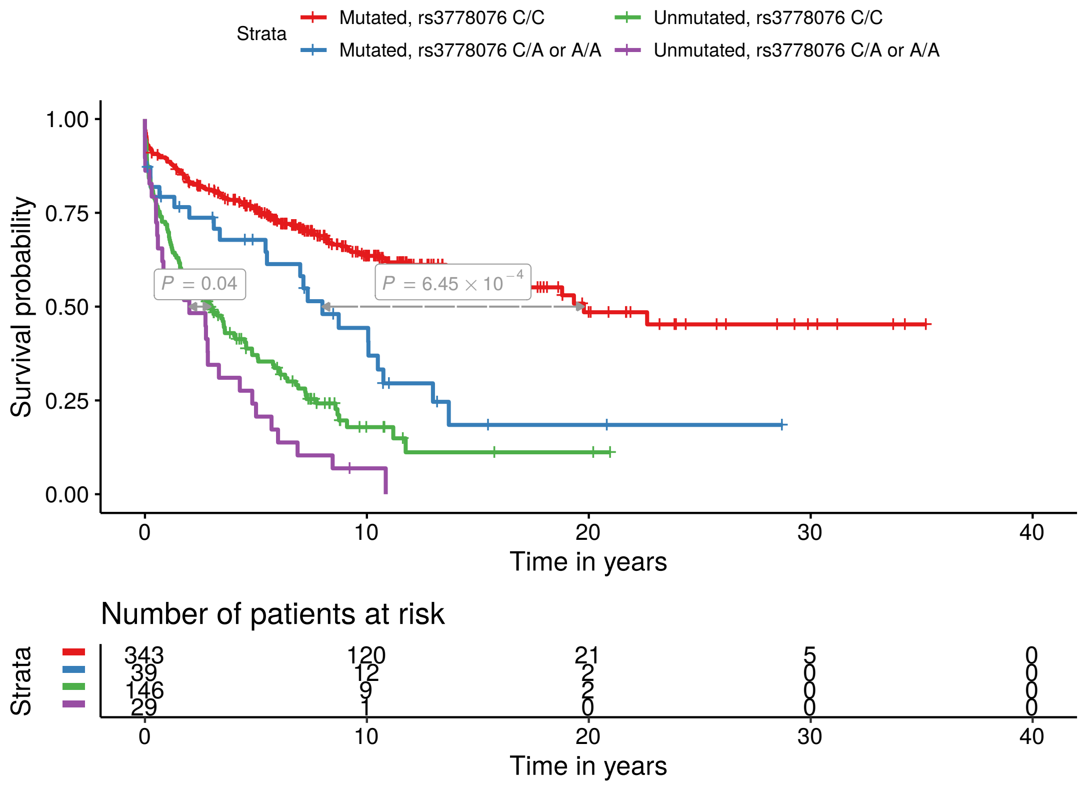

Supplementary Figure 13: **Kaplan-Meier plot showing time to first treatment (TTFT) for CLL patients stratified by IGHV status and SNP genotype for rs736456 (a) and rs3778076 (b).** P values are obtained from pairwise log-rank tests for survival curves, with false discovery rate (FDR) corrections for multiple testing. For simplicity, p values are only shown for the comparisions of SNP genotypes within the same prognostic factor stratum. TTFT is defined as the time from diagnosis of CLL to treatment or last follow-up without treatment. Patients censored at last follow-up are indicated by a cross. The number of patients in each group at each timepoint are indicated in the table. All statistical tests were two-sided.

a

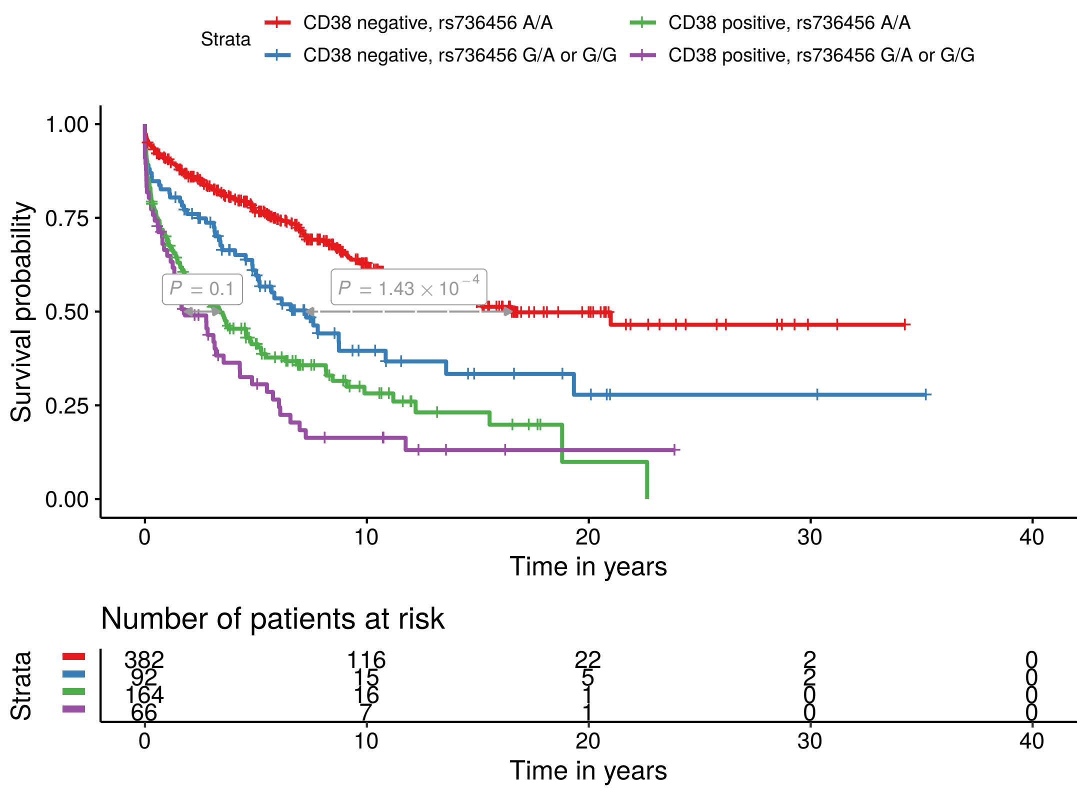

b

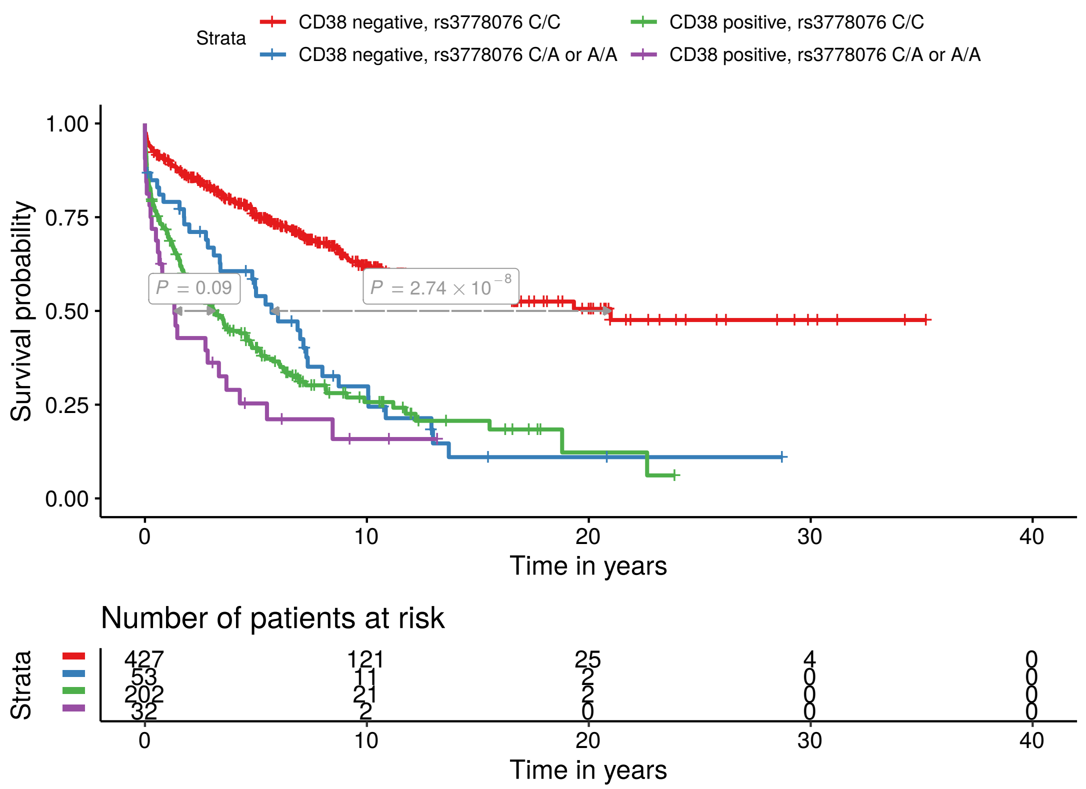

Supplementary Figure 14: **Kaplan-Meier plot showing time to first treatment (TTFT) for CLL patients stratified by CD38 status and SNP genotype for rs736456 (a) and rs3778076 (b).** P values are obtained from pairwise log-rank tests for survival curves, with false discovery rate (FDR) corrections for multiple testing. For simplicity, p values are only shown for the comparisions of SNP genotypes within the same prognostic factor stratum. TTFT is defined as the time from diagnosis of CLL to treatment or last follow-up without treatment. Patients censored at last follow-up are indicated by a cross. The number of patients in each group at each timepoint are indicated in the table. All statistical tests were two-sided.

a

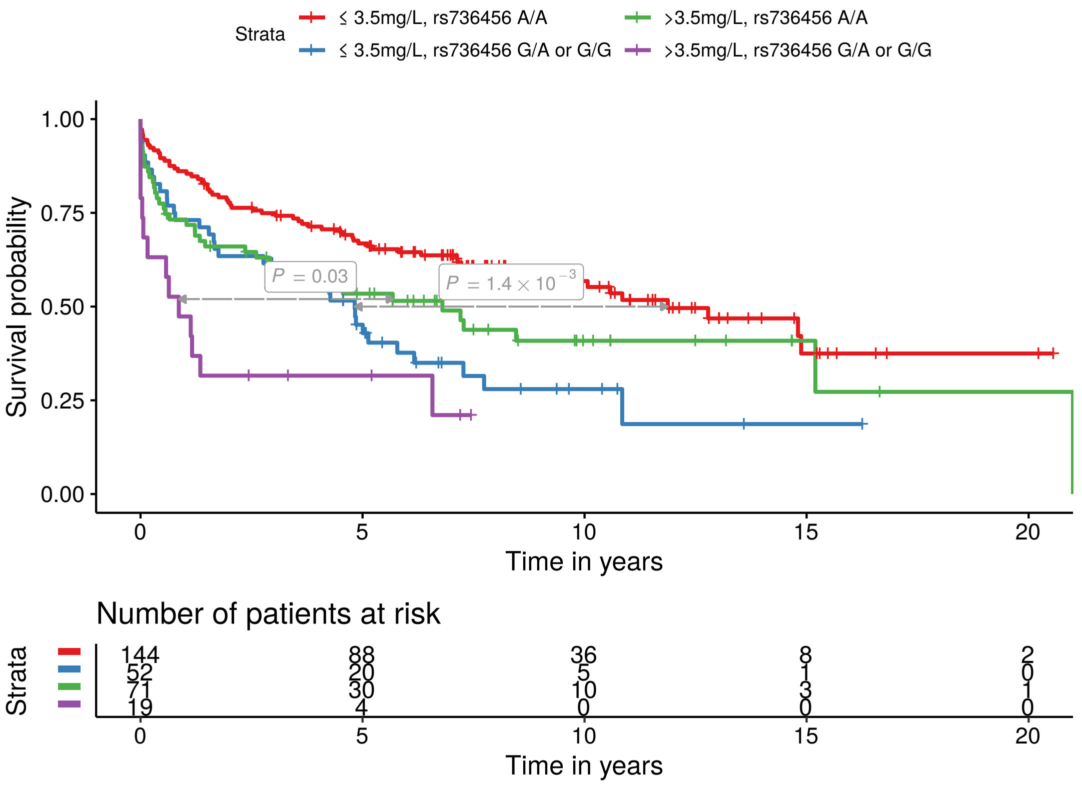

b

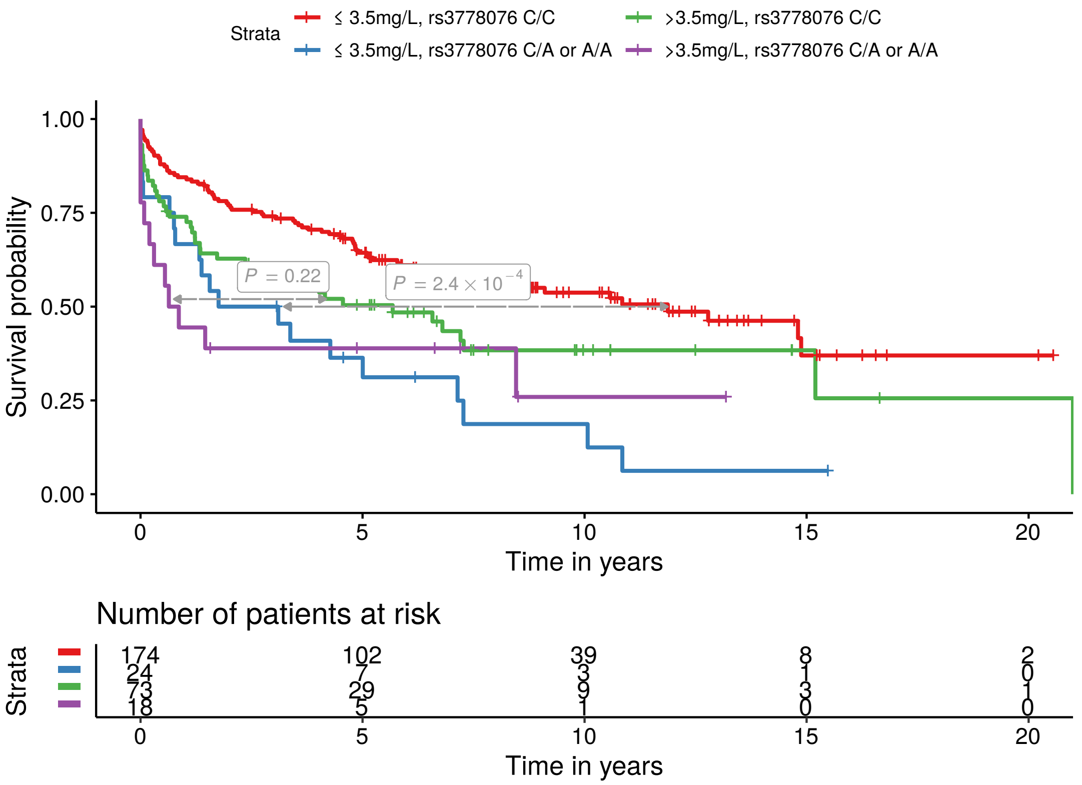

Supplementary Figure 15: **Kaplan-Meier plot showing time to first treatment (TTFT) for CLL patients stratified by $\beta 2$ Microglobulin and SNP genotype for rs736456 (a) and rs3778076 (b).** P values are obtained from pairwise log-rank tests for survival curves, with false discovery rate (FDR) corrections for multiple testing. For simplicity, p values are only shown for the comparisions of SNP genotypes within the same prognostic factor stratum. TTFT is defined as the time from diagnosis of CLL to treatment or last follow-up without treatment. Patients censored at last follow-up are indicated by a cross. The number of patients in each group at each timepoint are indicated in the table. High serum $\beta 2$ microglobulin is defined as > 3.5 mg/L. All statistical tests were two-sided.

a

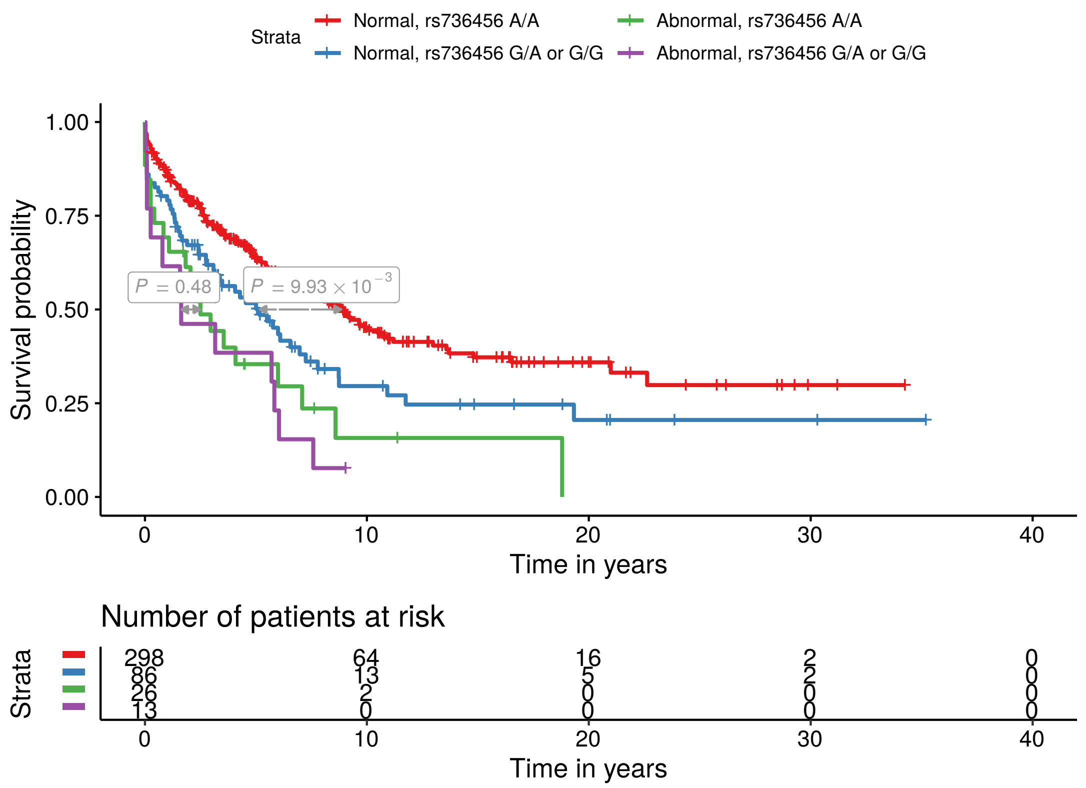

b

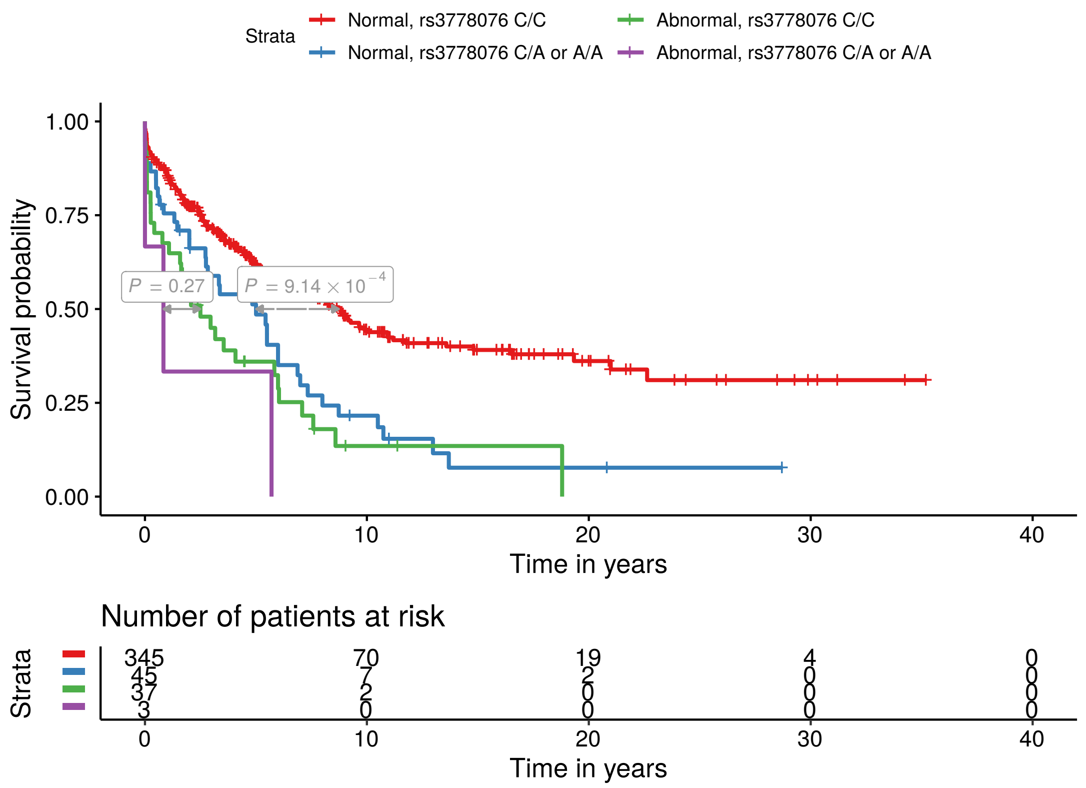

Supplementary Figure 16: **Kaplan-Meier plot showing time to first treatment (TTFT) for CLL patients stratified by TP53 status and SNP genotype for rs736456 (a) and rs3778076 (b).** P values are obtained from pairwise log-rank tests for survival curves, with false discovery rate (FDR) corrections for multiple testing. For simplicity, p values are only shown for the comparisions of SNP genotypes within the same prognostic factor stratum. TTFT is defined as the time from diagnosis of CLL to treatment or last follow-up without treatment. Patients censored at last follow-up are indicated by a cross. The number of patients in each group at each timepoint are indicated in the table. Abnormal is defined as a 17p deletion by FISH or a TP53 mutation by Sanger sequencing. It should be noted that TP53 status is not routinely determined in patients with early stage CLL. As such, the cohort tested for TP53 has an over-representation of patients with progressive CLL. All statistical tests were two-sided.

a

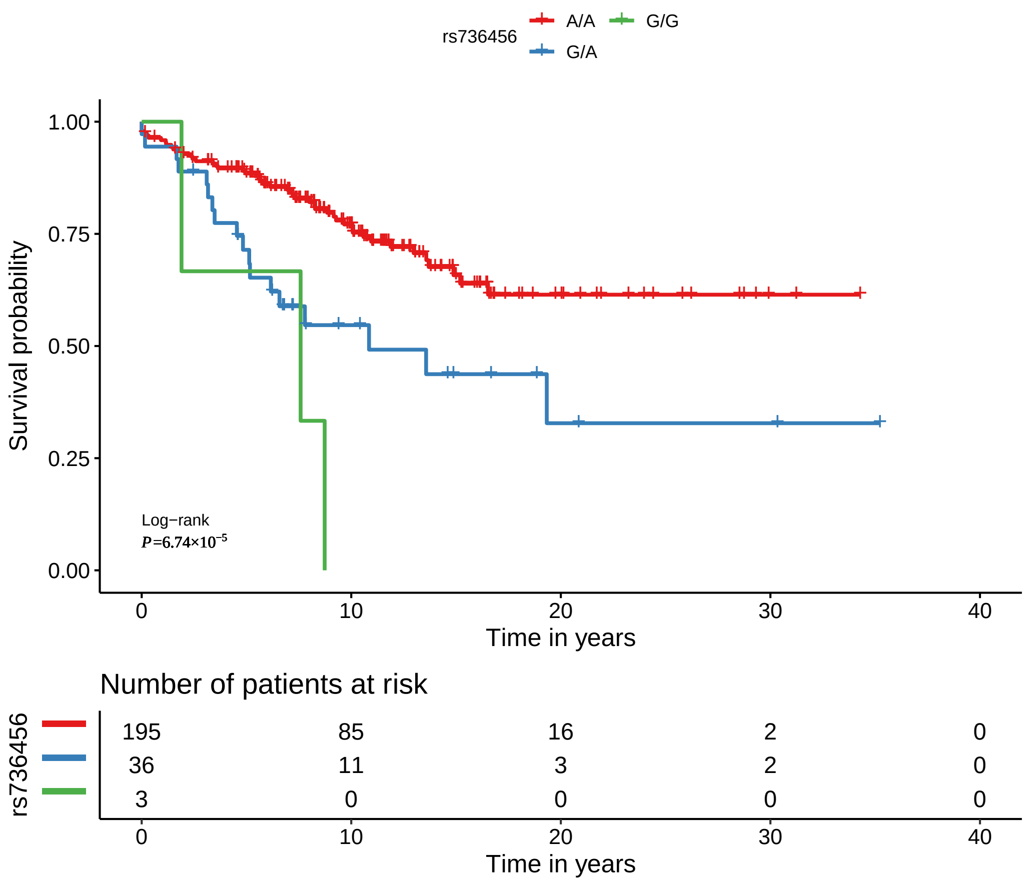
rs736456
A/A
G/G
G/A
Survival probability
1.00
0.75
0.50
0.25
0.00
Log−rank
P=6.74×10⁻⁵
0
10
20
30
40
Time in years
Number of patients at risk
rs736456
195 85 16 2 0
36 11 3 2 0
3 0 0 0 0
0
10
20
30
40
Time in years

b

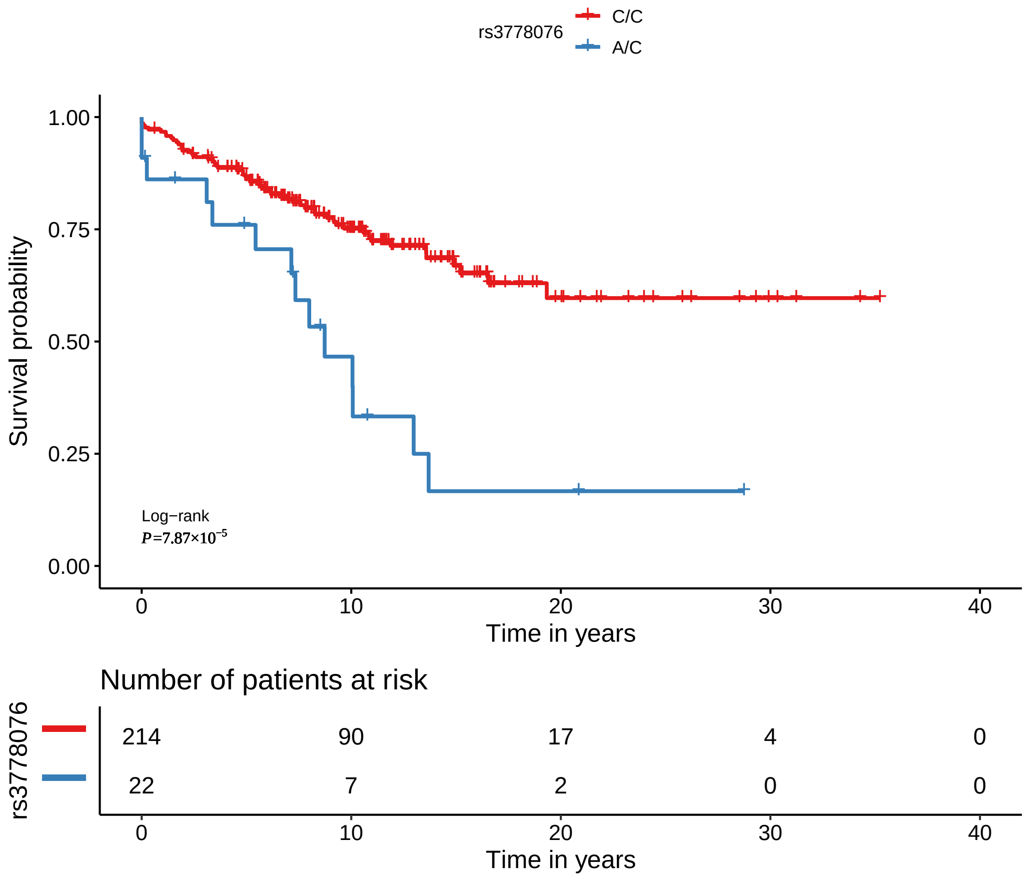
rs3778076
C/C
A/C
Survival probability
1.00
0.75
0.50
0.25
0.00
Log−rank
P=7.87×10⁻⁵
0
10
20
30
40
Time in years
Number of patients at risk
rs3778076
214 90 17 4 0
22 7 2 0 0
0
10
20
30
40
Time in years

c

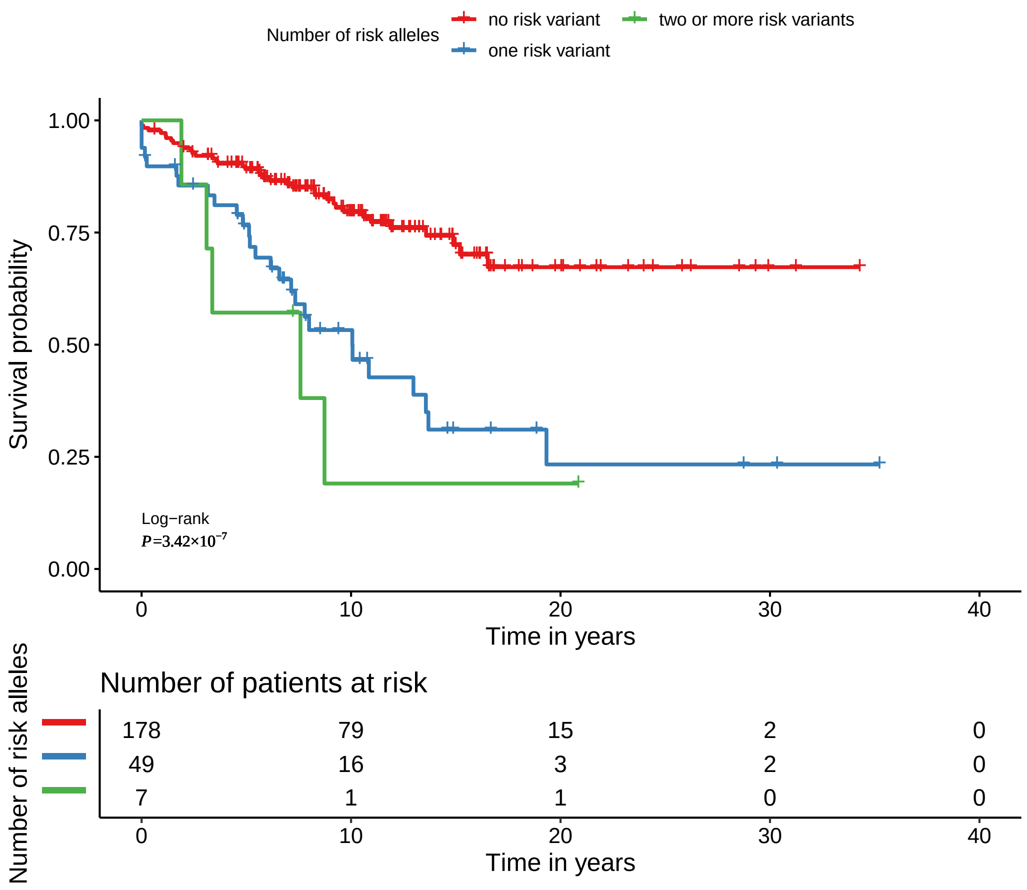

Supplementary Figure 17: **Survival curves in low-risk CLLs by rs736456 (a) rs3778076 (b) and risk allele groups of rs736456 and rs3778076 (c).** Low-risk CLL is defined as Binet stage A patients with mutated IGHV and CD38 negativity (N=236). Numbers do not match 236 because rs736456 genotypes are missing for 2 patients. Log-rank test is used to examine if survival curves differ by genotypes and risk allele group. All statistical tests were two-sided.

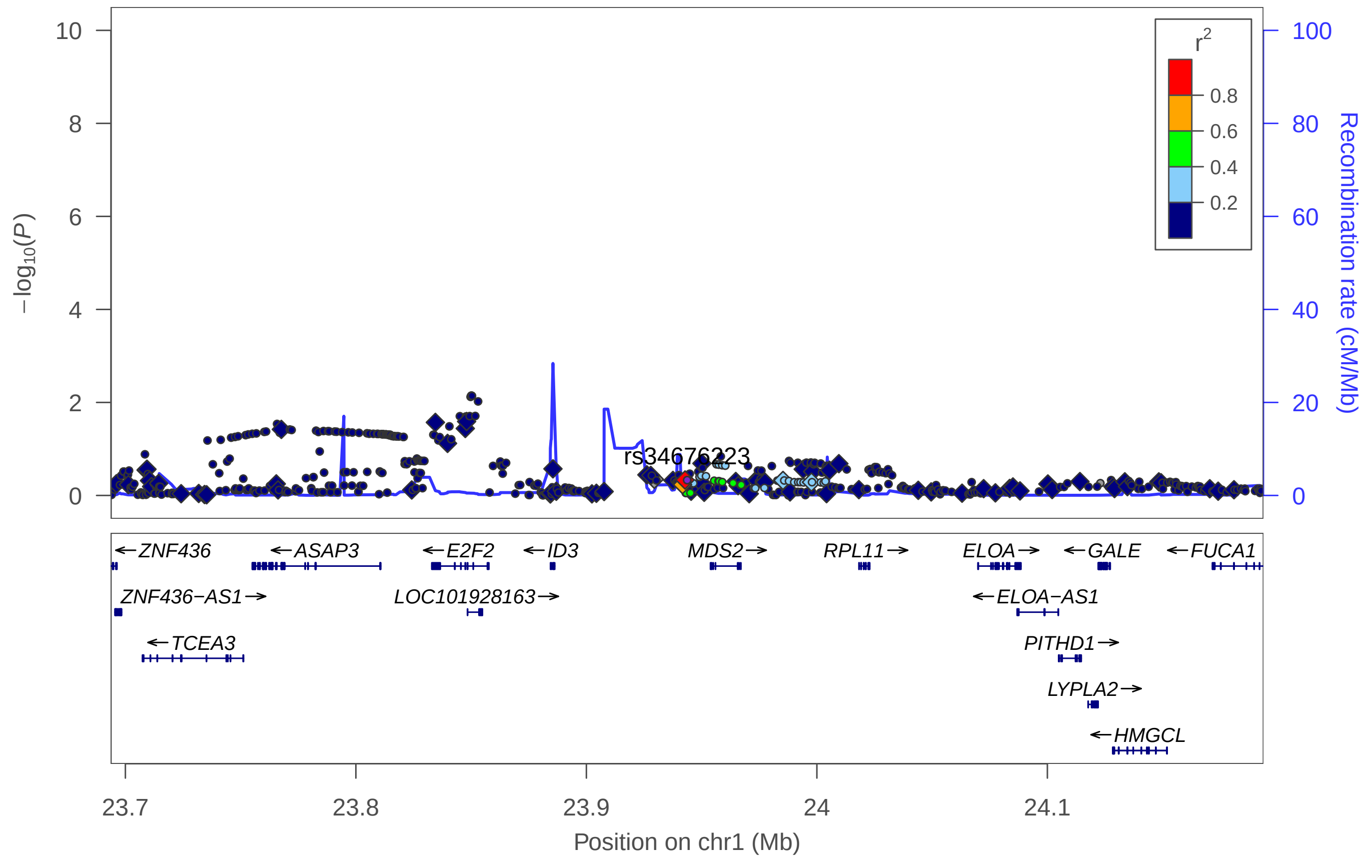

Supplementary Figure 18: **Regional association plot of TTFT (time to first time treatment) for known CLL etiological risk variant rs34676223**. For each study, allelic dosage was estimated for the minor allele at each variant position and included in a Cox proportional hazard model to estimate hazard ratio (HR) and 95% confidence interval (CI). Variants were included in the meta-analysis if they had results from all six studies. Study-specific single nucleotide polymorphism (SNP) effects were combined in a fixed effect model using an inverse-variance-weighted method. The upper panel shows SNP coordinates based on genomic build b37/hg19 on the x-axis, and -$\log_{10}$ (P values) on the y-axis. Genotyped and imputed SNPs are represented by diamonds and circles, respectively, and are coloured according to their linkage disequilibrium (pairwise r^2) with the CLL etiological risk variant based on the 1000 Genomes European panel. Reference genes in the region are shown in the lower panel, with arrows indicating transcript direction, dense blocks representing exons and horizontal lines representing introns.

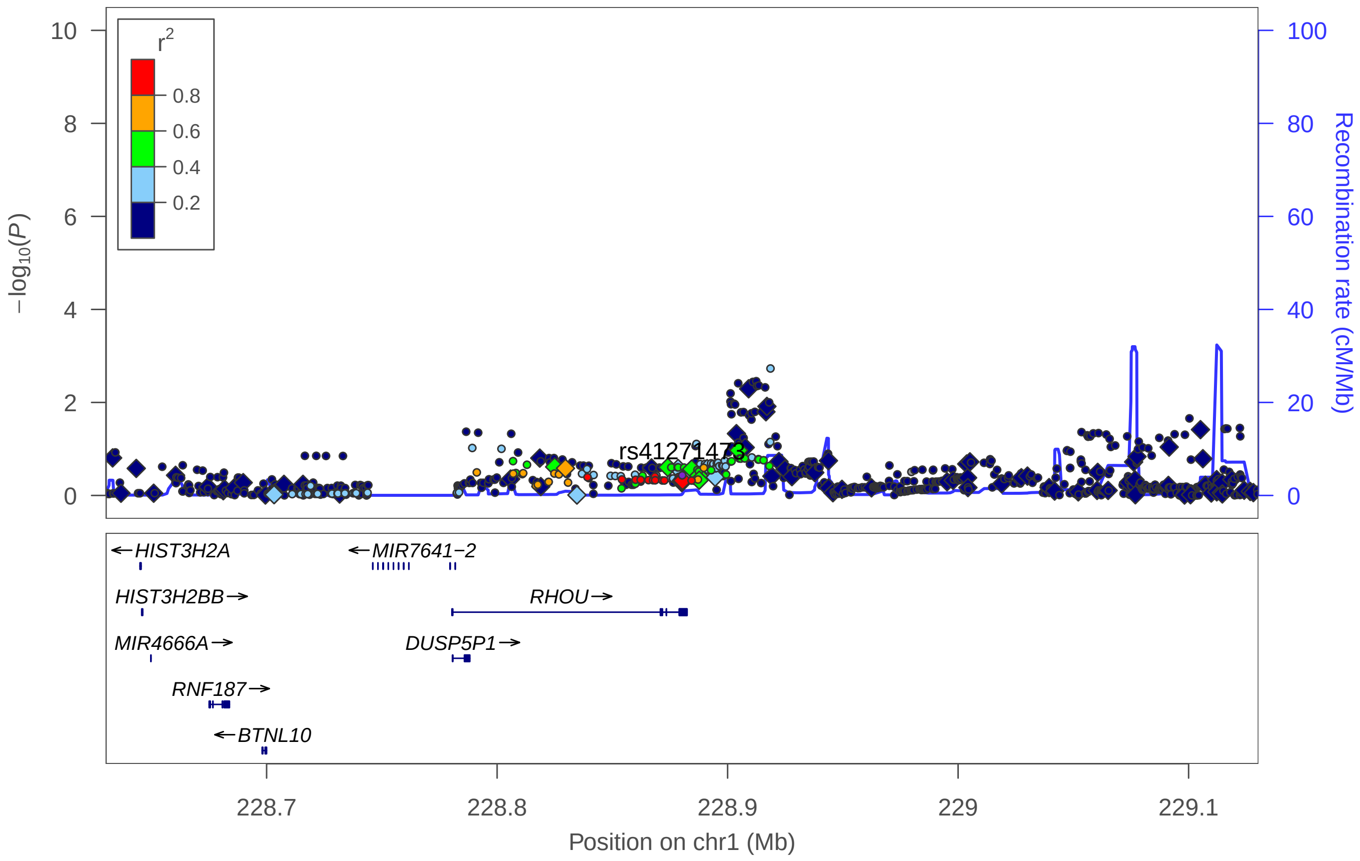

Supplementary Figure 19: **Regional association plot of TTFT (time to first time treatment) for known CLL etiological risk variant rs41271473**. For each study, allelic dosage was estimated for the minor allele at each variant position and included in a Cox proportional hazard model to estimate hazard ratio (HR) and 95% confidence interval (CI). Variants were included in the meta-analysis if they had results from all six studies. Study-specific single nucleotide polymorphism (SNP) effects were combined in a fixed effect model using an inverse-variance-weighted method. The upper panel shows SNP coordinates based on genomic build b37/hg19 on the x-axis, and -$\log_{10}$ (P values) on the y-axis. Genotyped and imputed SNPs are represented by diamonds and circles, respectively, and are coloured according to their linkage disequilibrium (pairwise r^2) with the CLL etiological risk variant based on the 1000 Genomes European panel. Reference genes in the region are shown in the lower panel, with arrows indicating transcript direction, dense blocks representing exons and horizontal lines representing introns.

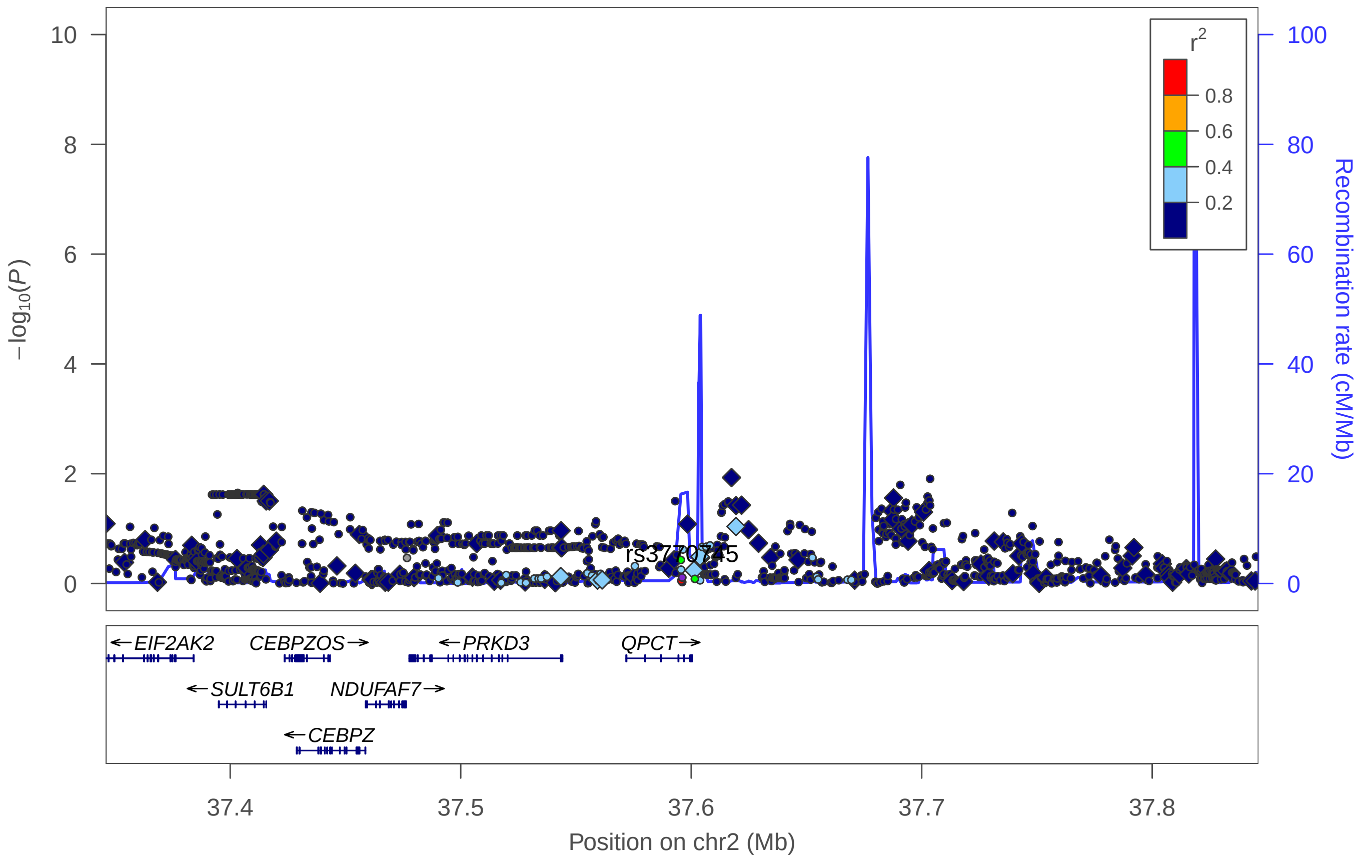

Supplementary Figure 20: **Regional association plot of TTFT (time to first time treatment) for known CLL etiological risk variant rs3770745**. For each study, allelic dosage was estimated for the minor allele at each variant position and included in a Cox proportional hazard model to estimate hazard ratio (HR) and 95% confidence interval (CI). Variants were included in the meta-analysis if they had results from all six studies. Study-specific single nucleotide polymorphism (SNP) effects were combined in a fixed effect model using an inverse-variance-weighted method. The upper panel shows SNP coordinates based on genomic build b37/hg19 on the x-axis, and -$\log_{10}$ (P values) on the y-axis. Genotyped and imputed SNPs are represented by diamonds and circles, respectively, and are coloured according to their linkage disequilibrium (pairwise r^2) with the CLL etiological risk variant based on the 1000 Genomes European panel. Reference genes in the region are shown in the lower panel, with arrows indicating transcript direction, dense blocks representing exons and horizontal lines representing introns.

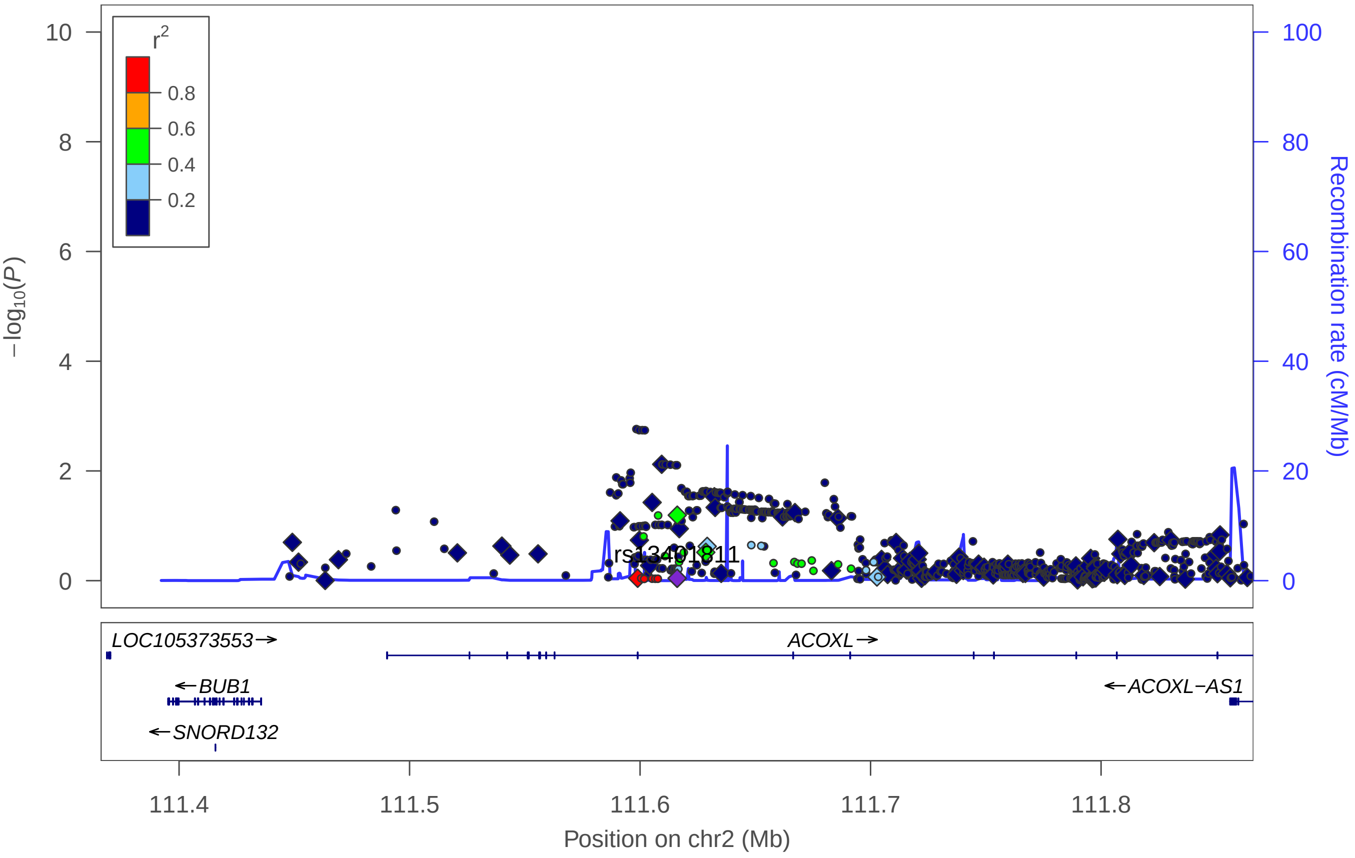

Supplementary Figure 21: **Regional association plot of TTFT (time to first time treatment) for known CLL etiological risk variant rs13401811**. For each study, allelic dosage was estimated for the minor allele at each variant position and included in a Cox proportional hazard model to estimate hazard ratio (HR) and 95% confidence interval (CI). Variants were included in the meta-analysis if they had results from all six studies. Study-specific single nucleotide polymorphism (SNP) effects were combined in a fixed effect model using an inverse-variance-weighted method. The upper panel shows SNP coordinates based on genomic build b37/hg19 on the x-axis, and -$\log_{10}$ (P values) on the y-axis. Genotyped and imputed SNPs are represented by diamonds and circles, respectively, and are coloured according to their linkage disequilibrium (pairwise r^2) with the CLL etiological risk variant based on the 1000 Genomes European panel. Reference genes in the region are shown in the lower panel, with arrows indicating transcript direction, dense blocks representing exons and horizontal lines representing introns.

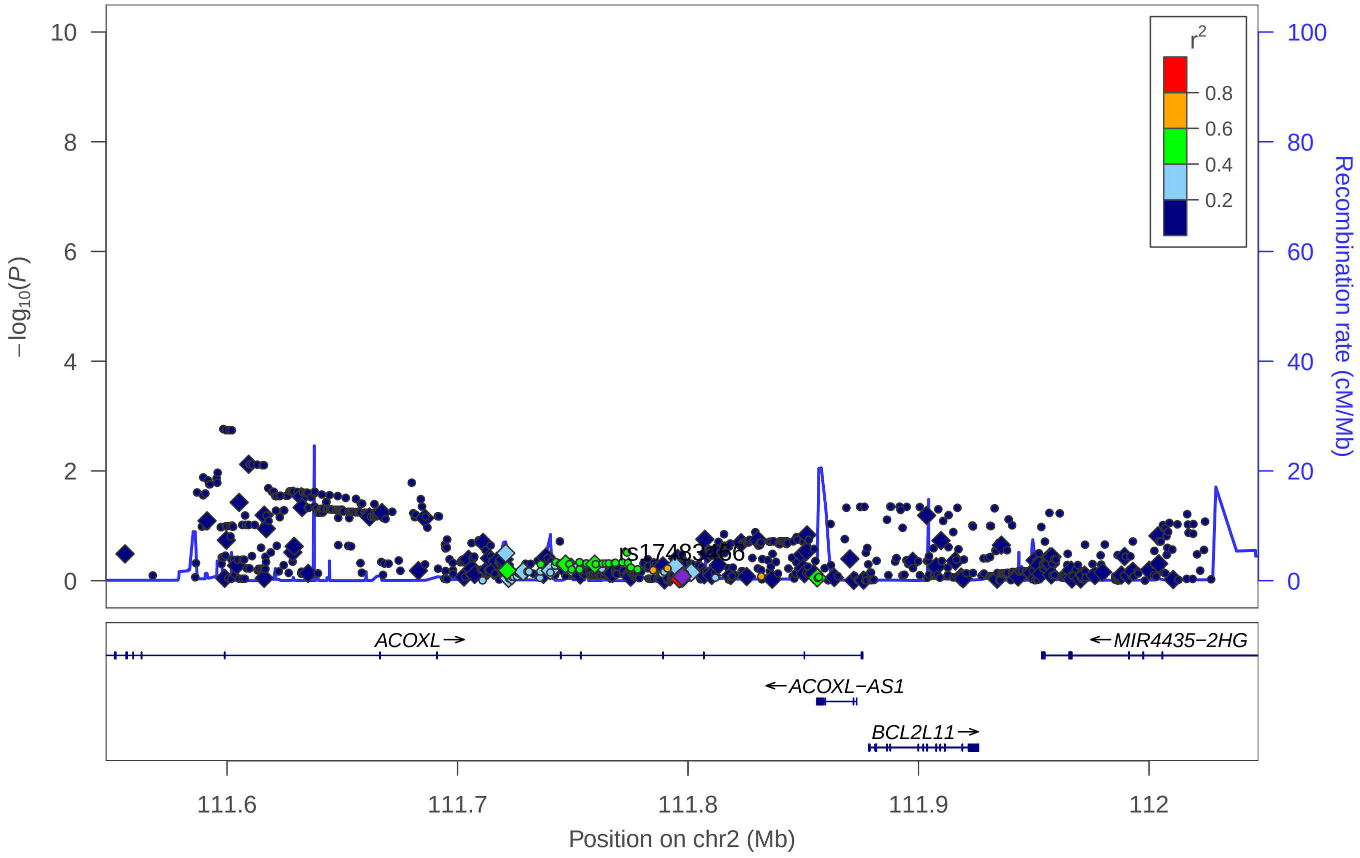

Supplementary Figure 22: **Regional association plot of TTFT (time to first time treatment) for known CLL etiological risk variant rs17483466**. For each study, allelic dosage was estimated for the minor allele at each variant position and included in a Cox proportional hazard model to estimate hazard ratio (HR) and 95% confidence interval (CI). Variants were included in the meta-analysis if they had results from all six studies. Study-specific single nucleotide polymorphism (SNP) effects were combined in a fixed effect model using an inverse-variance-weighted method. The upper panel shows SNP coordinates based on genomic build b37/hg19 on the x-axis, and -$\log_{10}$ (P values) on the y-axis. Genotyped and imputed SNPs are represented by diamonds and circles, respectively, and are coloured according to their linkage disequilibrium (pairwise r^2) with the CLL etiological risk variant based on the 1000 Genomes European panel. Reference genes in the region are shown in the lower panel, with arrows indicating transcript direction, dense blocks representing exons and horizontal lines representing introns.

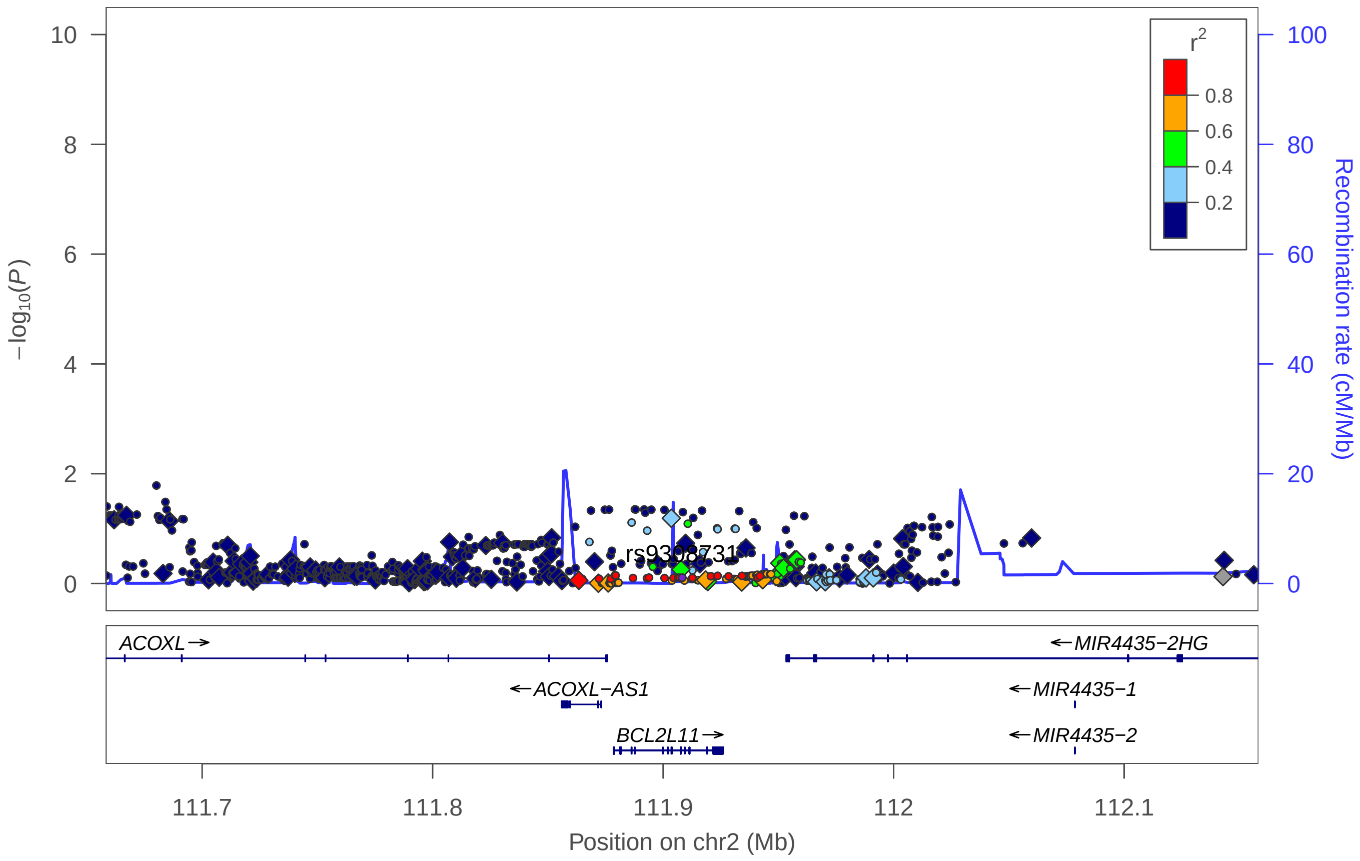

Supplementary Figure 23: **Regional association plot of TTFT (time to first time treatment) for known CLL etiological risk variant rs9308731**. For each study, allelic dosage was estimated for the minor allele at each variant position and included in a Cox proportional hazard model to estimate hazard ratio (HR) and 95% confidence interval (CI). Variants were included in the meta-analysis if they had results from all six studies. Study-specific single nucleotide polymorphism (SNP) effects were combined in a fixed effect model using an inverse-variance-weighted method. The upper panel shows SNP coordinates based on genomic build b37/hg19 on the x-axis, and -$\log_{10}$ (P values) on the y-axis. Genotyped and imputed SNPs are represented by diamonds and circles, respectively, and are coloured according to their linkage disequilibrium (pairwise r^2) with the CLL etiological risk variant based on the 1000 Genomes European panel. Reference genes in the region are shown in the lower panel, with arrows indicating transcript direction, dense blocks representing exons and horizontal lines representing introns.

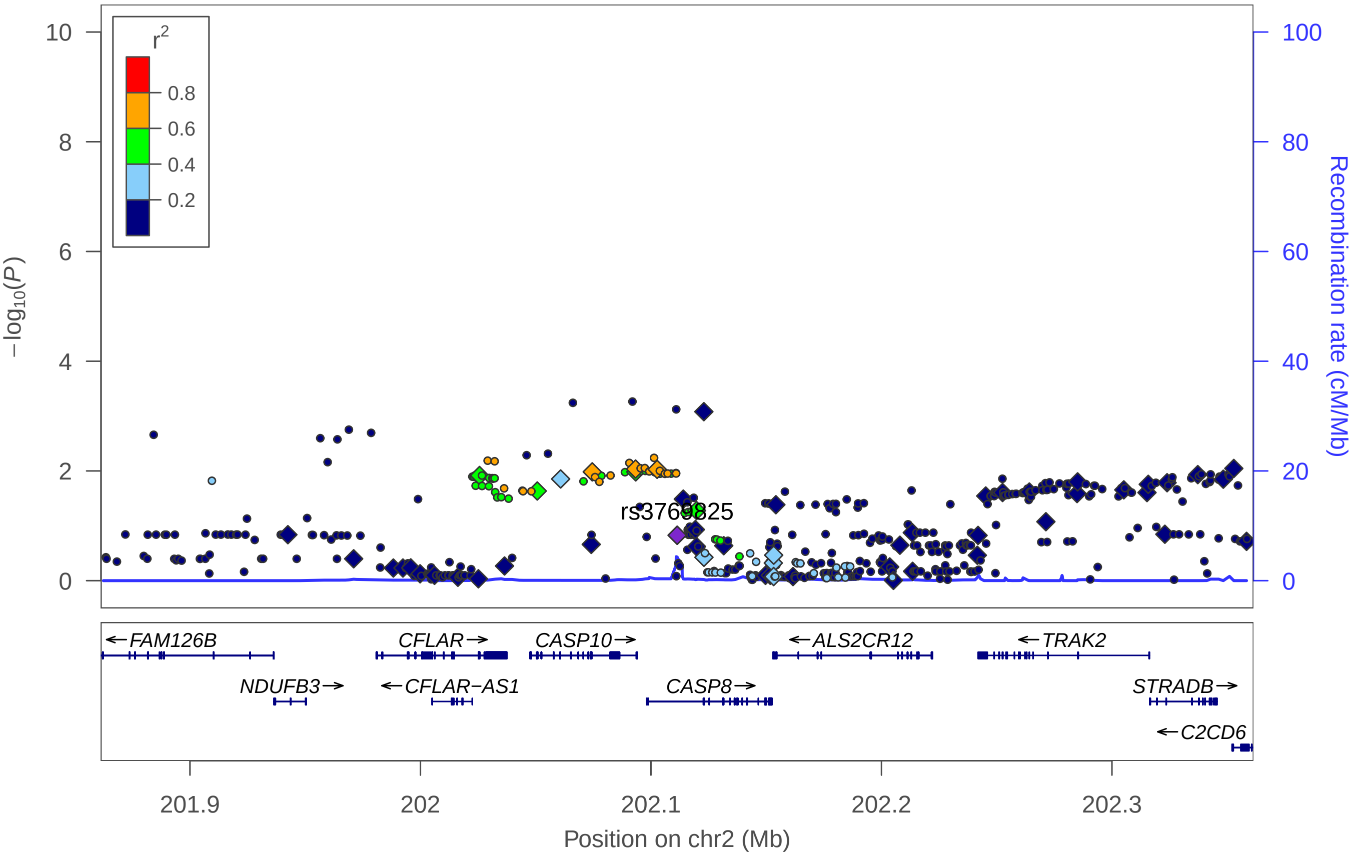

Supplementary Figure 24: **Regional association plot of TTFT (time to first time treatment) for known CLL etiological risk variant rs3769825**. For each study, allelic dosage was estimated for the minor allele at each variant position and included in a Cox proportional hazard model to estimate hazard ratio (HR) and 95% confidence interval (CI). Variants were included in the meta-analysis if they had results from all six studies. Study-specific single nucleotide polymorphism (SNP) effects were combined in a fixed effect model using an inverse-variance-weighted method. The upper panel shows SNP coordinates based on genomic build b37/hg19 on the x-axis, and -log$_{10}$ (P values) on the y-axis. Genotyped and imputed SNPs are represented by diamonds and circles, respectively, and are coloured according to their linkage disequilibrium (pairwise r^2) with the CLL etiological risk variant based on the 1000 Genomes European panel. Reference genes in the region are shown in the lower panel, with arrows indicating transcript direction, dense blocks representing exons and horizontal lines representing introns.

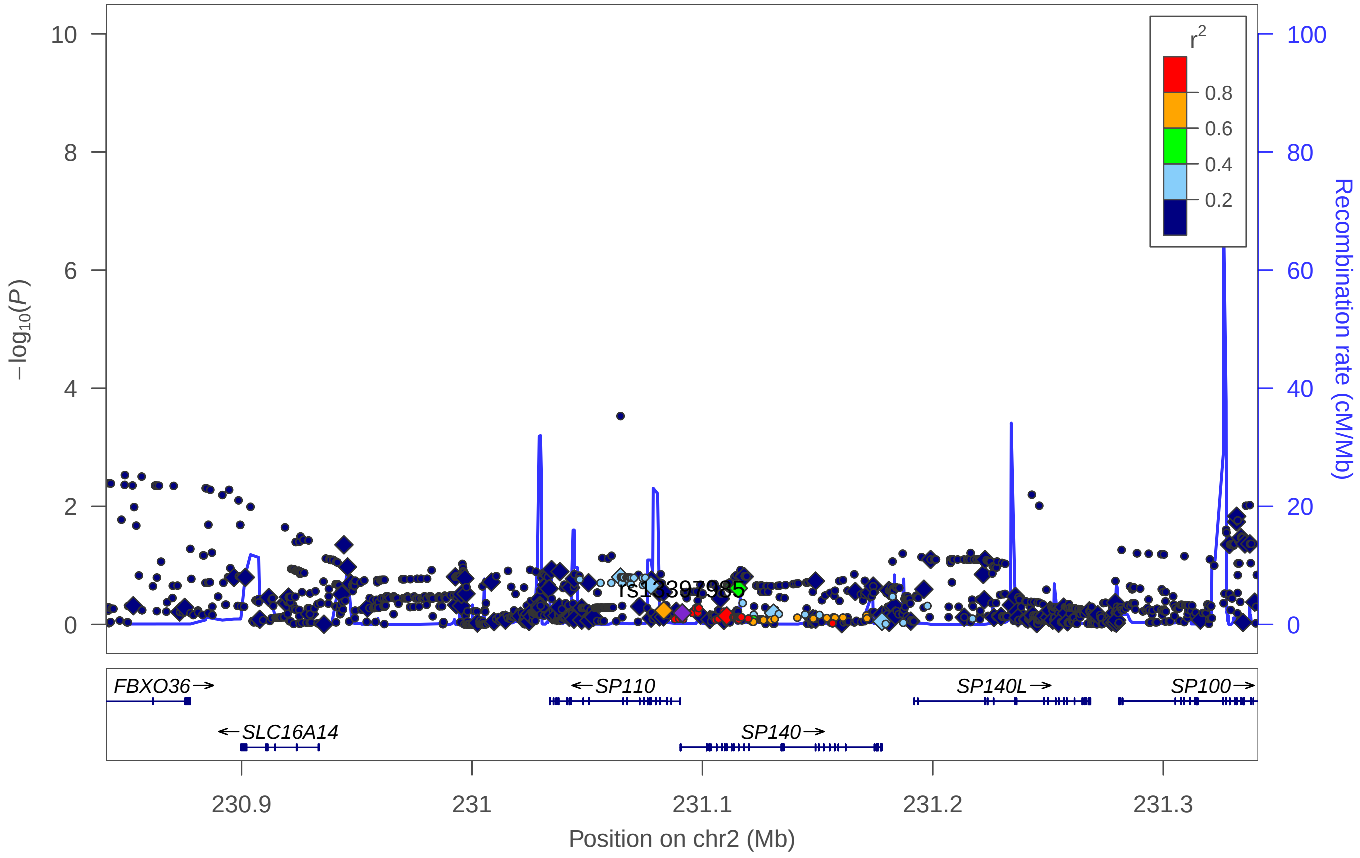

Supplementary Figure 25: **Regional association plot of TTFT (time to first time treatment) for known CLL etiological risk variant rs13397985**. For each study, allelic dosage was estimated for the minor allele at each variant position and included in a Cox proportional hazard model to estimate hazard ratio (HR) and 95% confidence interval (CI). Variants were included in the meta-analysis if they had results from all six studies. Study-specific single nucleotide polymorphism (SNP) effects were combined in a fixed effect model using an inverse-variance-weighted method. The upper panel shows SNP coordinates based on genomic build b37/hg19 on the x-axis, and -$\log_{10}$ (P values) on the y-axis. Genotyped and imputed SNPs are represented by diamonds and circles, respectively, and are coloured according to their linkage disequilibrium (pairwise r^2) with the CLL etiological risk variant based on the 1000 Genomes European panel. Reference genes in the region are shown in the lower panel, with arrows indicating transcript direction, dense blocks representing exons and horizontal lines representing introns.

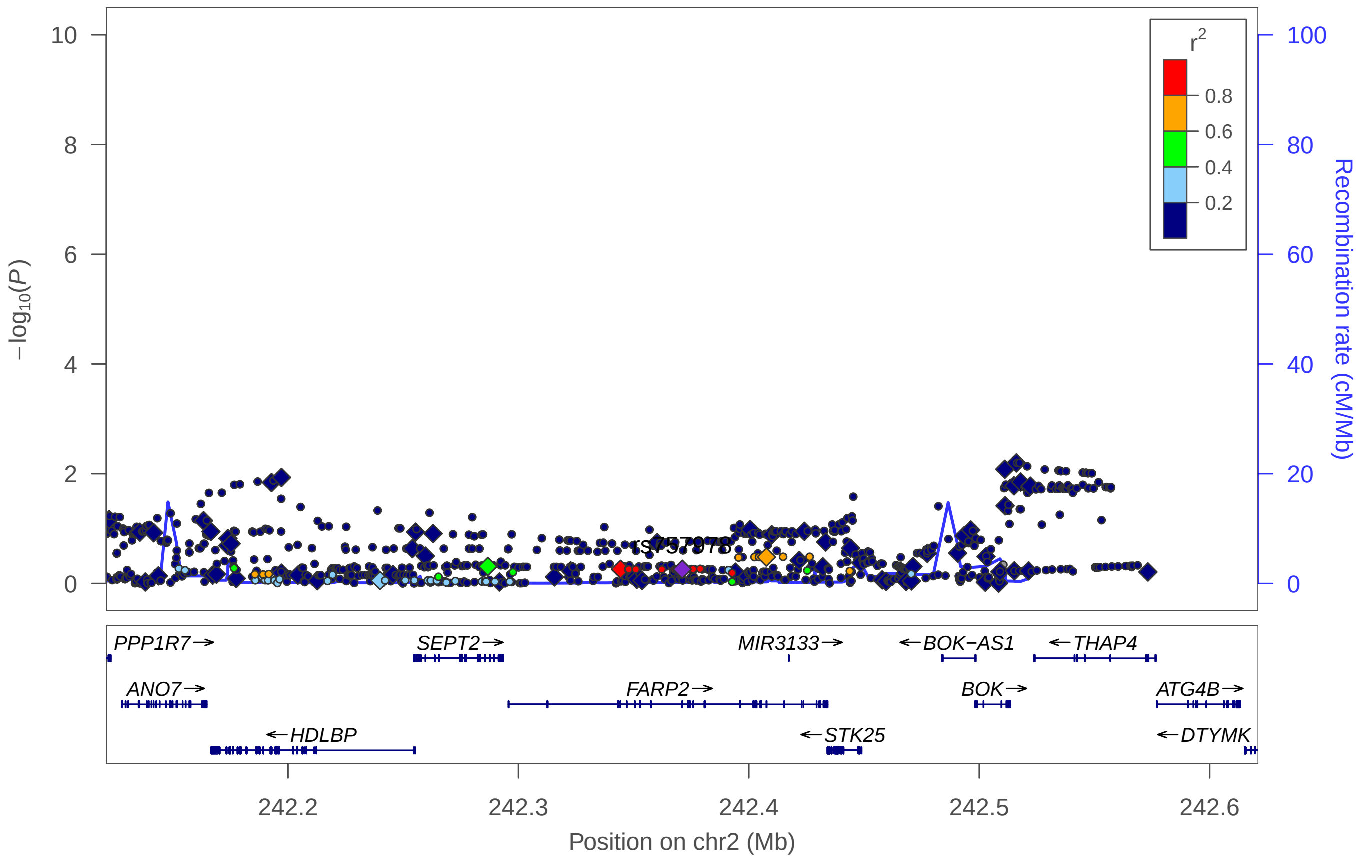

Supplementary Figure 26: **Regional association plot of TTFT (time to first time treatment) for known CLL etiological risk variant rs757978**. For each study, allelic dosage was estimated for the minor allele at each variant position and included in a Cox proportional hazard model to estimate hazard ratio (HR) and 95% confidence interval (CI). Variants were included in the meta-analysis if they had results from all six studies. Study-specific single nucleotide polymorphism (SNP) effects were combined in a fixed effect model using an inverse-variance-weighted method. The upper panel shows SNP coordinates based on genomic build b37/hg19 on the x-axis, and -$\log_{10}$ (P values) on the y-axis. Genotyped and imputed SNPs are represented by diamonds and circles, respectively, and are coloured according to their linkage disequilibrium (pairwise r^2) with the CLL etiological risk variant based on the 1000 Genomes European panel. Reference genes in the region are shown in the lower panel, with arrows indicating transcript direction, dense blocks representing exons and horizontal lines representing introns.

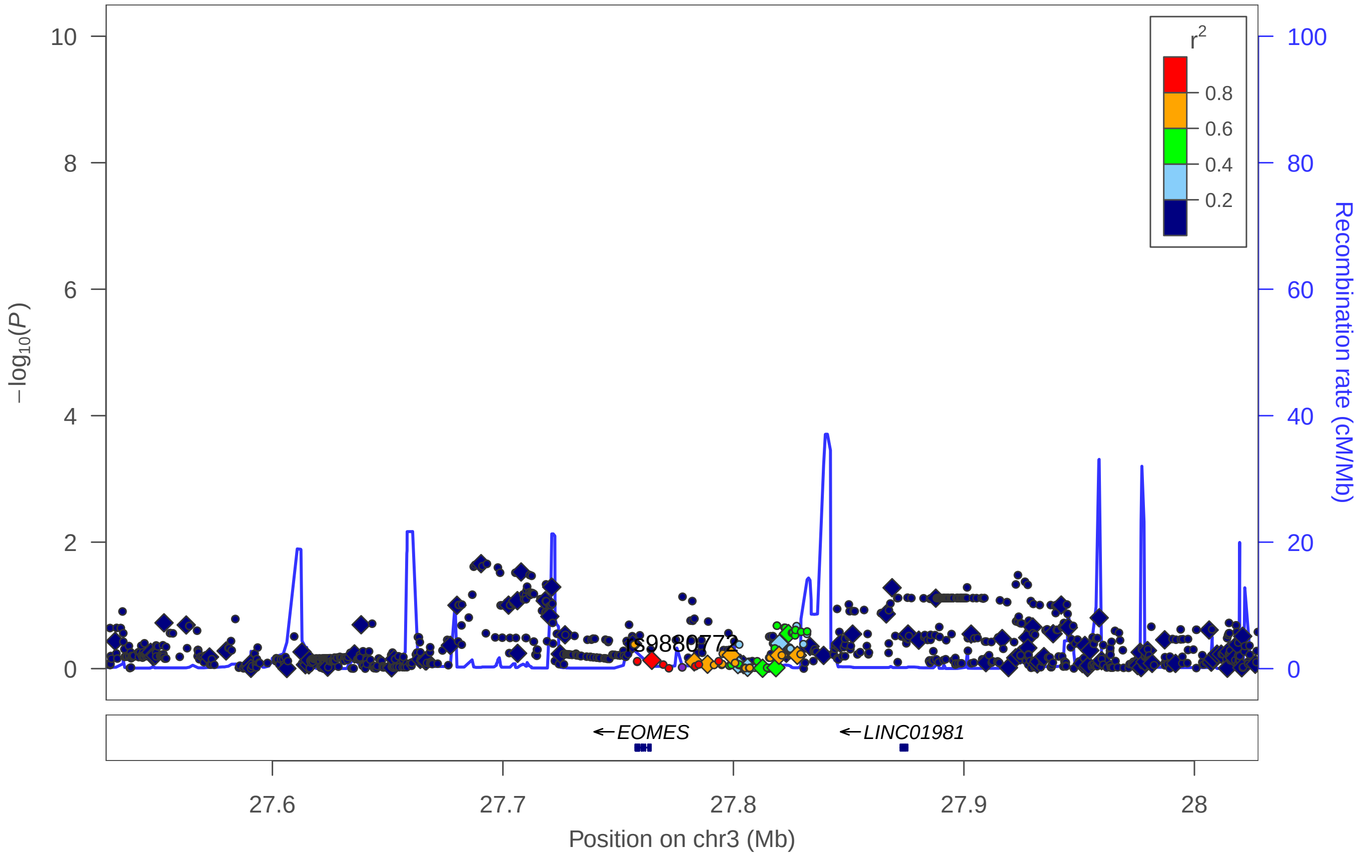

Supplementary Figure 27: **Regional association plot of TTFT (time to first time treatment) for known CLL etiological risk variant rs9880772**. For each study, allelic dosage was estimated for the minor allele at each variant position and included in a Cox proportional hazard model to estimate hazard ratio (HR) and 95% confidence interval (CI). Variants were included in the meta-analysis if they had results from all six studies. Study-specific single nucleotide polymorphism (SNP) effects were combined in a fixed effect model using an inverse-variance-weighted method. The upper panel shows SNP coordinates based on genomic build b37/hg19 on the x-axis, and -$\log_{10}$ (P values) on the y-axis. Genotyped and imputed SNPs are represented by diamonds and circles, respectively, and are coloured according to their linkage disequilibrium (pairwise r^2) with the CLL etiological risk variant based on the 1000 Genomes European panel. Reference genes in the region are shown in the lower panel, with arrows indicating transcript direction, dense blocks representing exons and horizontal lines representing introns.

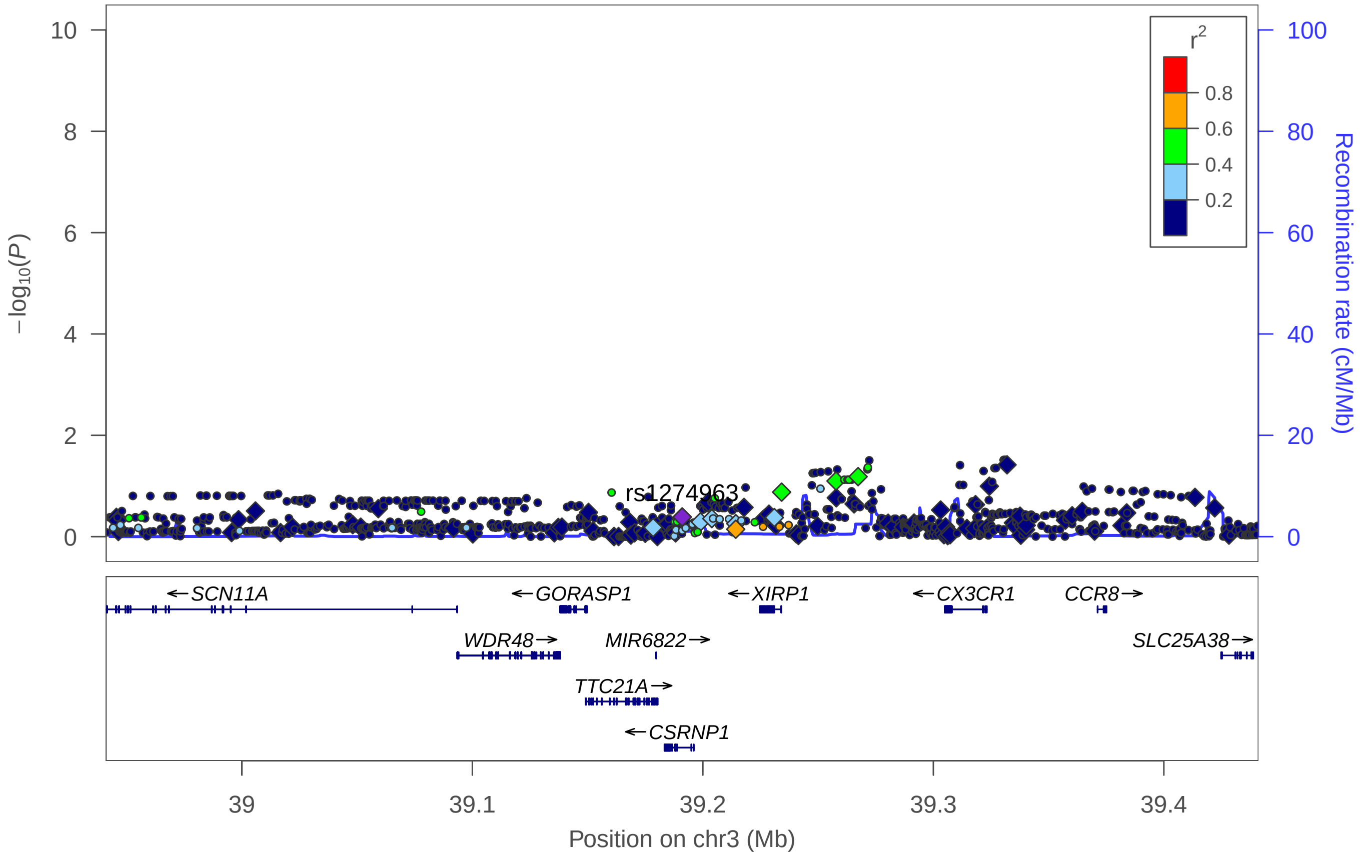

Supplementary Figure 28: **Regional association plot of TTFT (time to first time treatment) for known CLL etiological risk variant rs1274963**. For each study, allelic dosage was estimated for the minor allele at each variant position and included in a Cox proportional hazard model to estimate hazard ratio (HR) and 95% confidence interval (CI). Variants were included in the meta-analysis if they had results from all six studies. Study-specific single nucleotide polymorphism (SNP) effects were combined in a fixed effect model using an inverse-variance-weighted method. The upper panel shows SNP coordinates based on genomic build b37/hg19 on the x-axis, and -$\log_{10}$ (P values) on the y-axis. Genotyped and imputed SNPs are represented by diamonds and circles, respectively, and are coloured according to their linkage disequilibrium (pairwise r^2) with the CLL etiological risk variant based on the 1000 Genomes European panel. Reference genes in the region are shown in the lower panel, with arrows indicating transcript direction, dense blocks representing exons and horizontal lines representing introns.

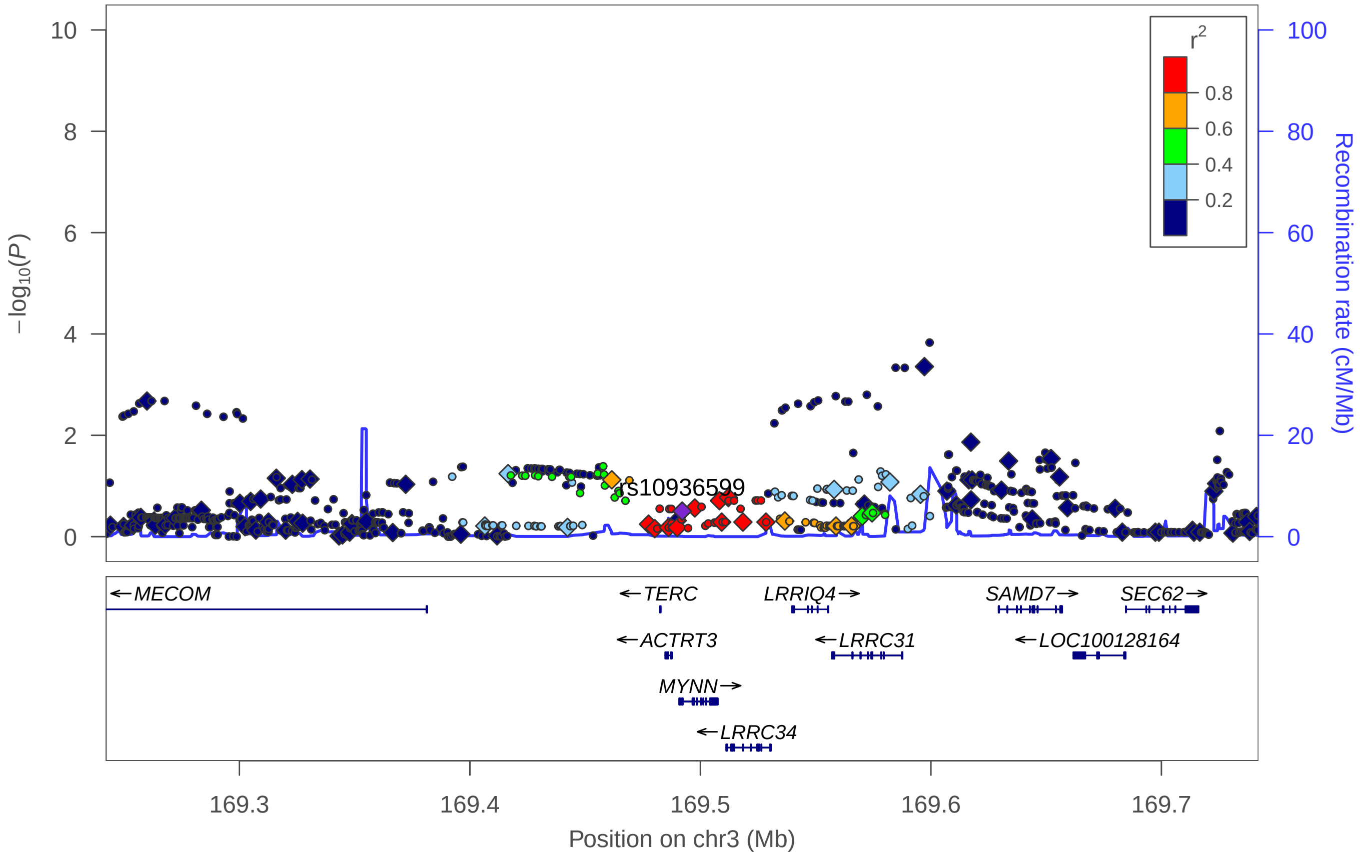

Supplementary Figure 29: **Regional association plot of TTFT (time to first time treatment) for known CLL etiological risk variant rs10936599**. For each study, allelic dosage was estimated for the minor allele at each variant position and included in a Cox proportional hazard model to estimate hazard ratio (HR) and 95% confidence interval (CI). Variants were included in the meta-analysis if they had results from all six studies. Study-specific single nucleotide polymorphism (SNP) effects were combined in a fixed effect model using an inverse-variance-weighted method. The upper panel shows SNP coordinates based on genomic build b37/hg19 on the x-axis, and -$\log_{10}$ (P values) on the y-axis. Genotyped and imputed SNPs are represented by diamonds and circles, respectively, and are coloured according to their linkage disequilibrium (pairwise r^2) with the CLL etiological risk variant based on the 1000 Genomes European panel. Reference genes in the region are shown in the lower panel, with arrows indicating transcript direction, dense blocks representing exons and horizontal lines representing introns.

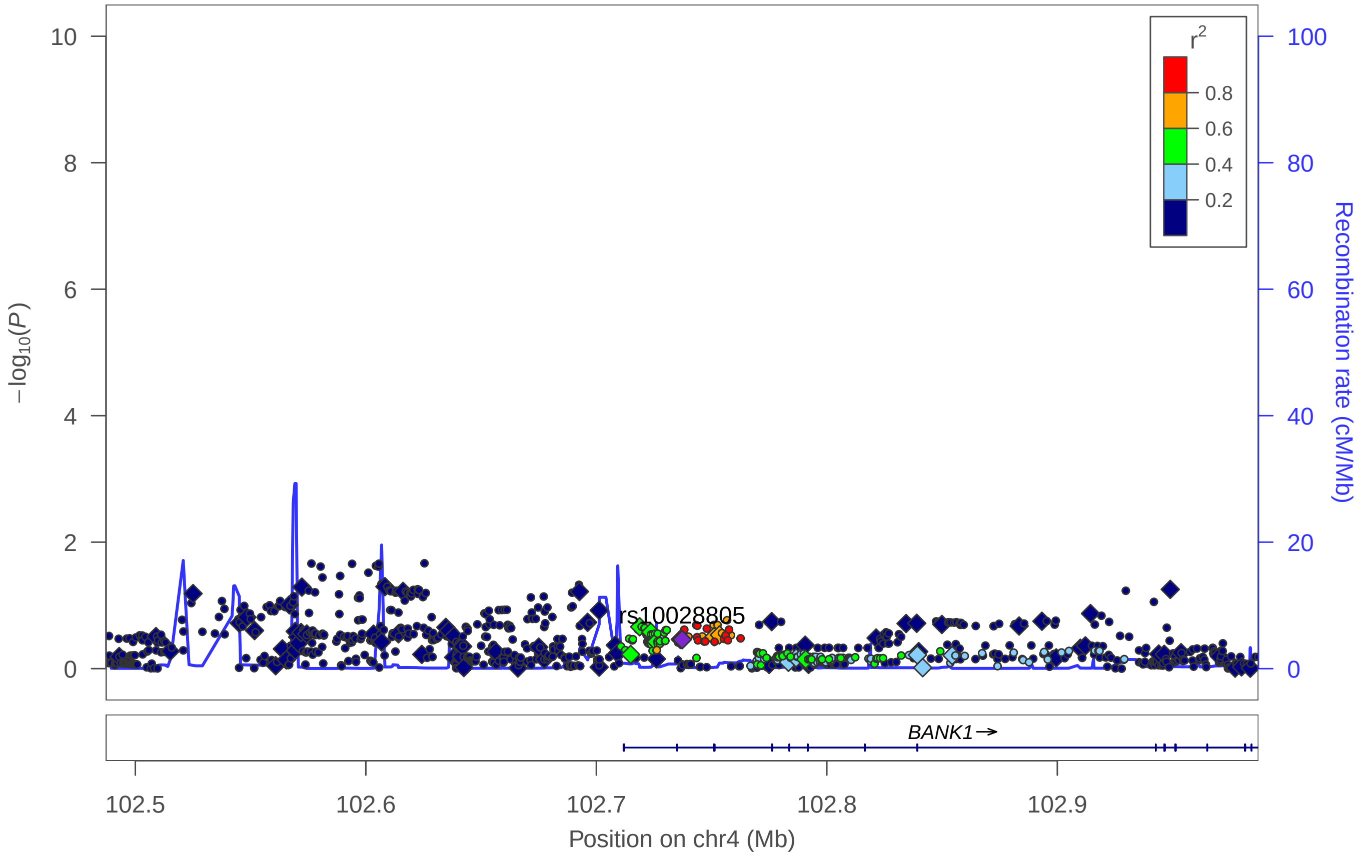

Supplementary Figure 30: **Regional association plot of TTFT (time to first time treatment) for known CLL etiological risk variant rs10028805**. For each study, allelic dosage was estimated for the minor allele at each variant position and included in a Cox proportional hazard model to estimate hazard ratio (HR) and 95% confidence interval (CI). Variants were included in the meta-analysis if they had results from all six studies. Study-specific single nucleotide polymorphism (SNP) effects were combined in a fixed effect model using an inverse-variance-weighted method. The upper panel shows SNP coordinates based on genomic build b37/hg19 on the x-axis, and -$\log_{10}$ (P values) on the y-axis. Genotyped and imputed SNPs are represented by diamonds and circles, respectively, and are coloured according to their linkage disequilibrium (pairwise r^2) with the CLL etiological risk variant based on the 1000 Genomes European panel. Reference genes in the region are shown in the lower panel, with arrows indicating transcript direction, dense blocks representing exons and horizontal lines representing introns.

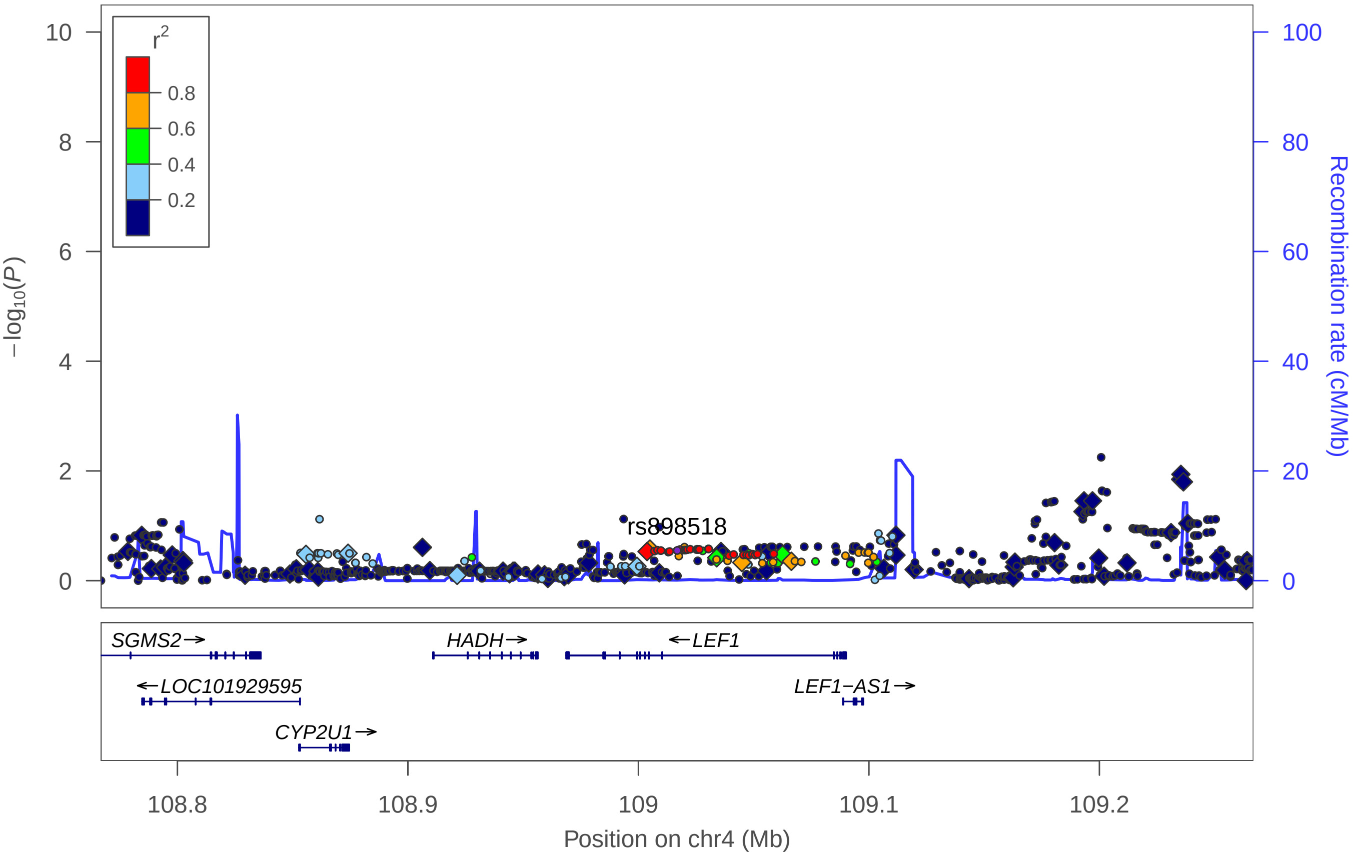

Supplementary Figure 31: **Regional association plot of TTFT (time to first time treatment) for known CLL etiological risk variant rs898518**. For each study, allelic dosage was estimated for the minor allele at each variant position and included in a Cox proportional hazard model to estimate hazard ratio (HR) and 95% confidence interval (CI). Variants were included in the meta-analysis if they had results from all six studies. Study-specific single nucleotide polymorphism (SNP) effects were combined in a fixed effect model using an inverse-variance-weighted method. The upper panel shows SNP coordinates based on genomic build b37/hg19 on the x-axis, and -$\log_{10}$ (P values) on the y-axis. Genotyped and imputed SNPs are represented by diamonds and circles, respectively, and are coloured according to their linkage disequilibrium (pairwise r^2) with the CLL etiological risk variant based on the 1000 Genomes European panel. Reference genes in the region are shown in the lower panel, with arrows indicating transcript direction, dense blocks representing exons and horizontal lines representing introns.

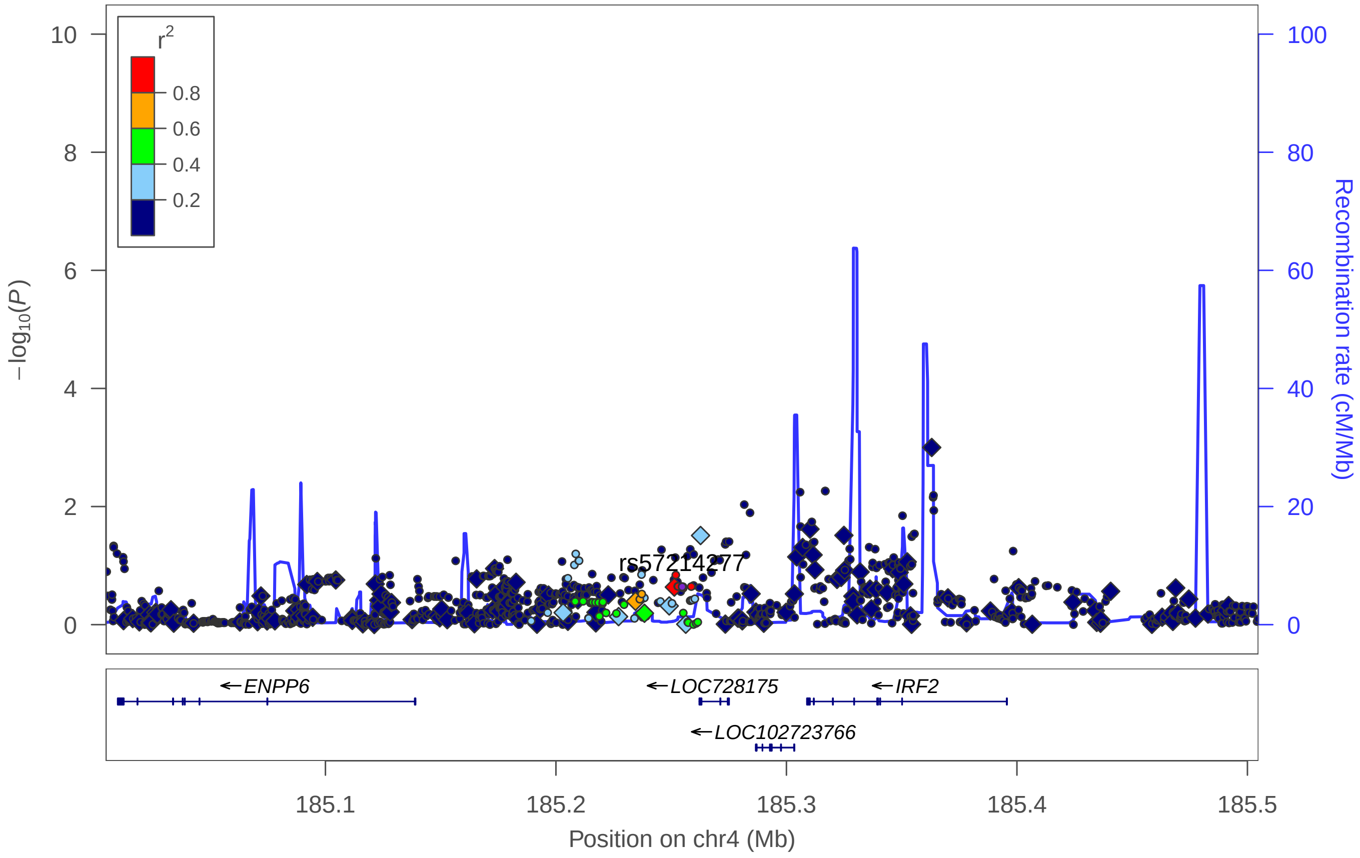

Supplementary Figure 32: **Regional association plot of TTFT (time to first time treatment) for known CLL etiological risk variant rs57214277**. For each study, allelic dosage was estimated for the minor allele at each variant position and included in a Cox proportional hazard model to estimate hazard ratio (HR) and 95% confidence interval (CI). Variants were included in the meta-analysis if they had results from all six studies. Study-specific single nucleotide polymorphism (SNP) effects were combined in a fixed effect model using an inverse-variance-weighted method. The upper panel shows SNP coordinates based on genomic build b37/hg19 on the x-axis, and -$\log_{10}$ (P values) on the y-axis. Genotyped and imputed SNPs are represented by diamonds and circles, respectively, and are coloured according to their linkage disequilibrium (pairwise r^2) with the CLL etiological risk variant based on the 1000 Genomes European panel. Reference genes in the region are shown in the lower panel, with arrows indicating transcript direction, dense blocks representing exons and horizontal lines representing introns.

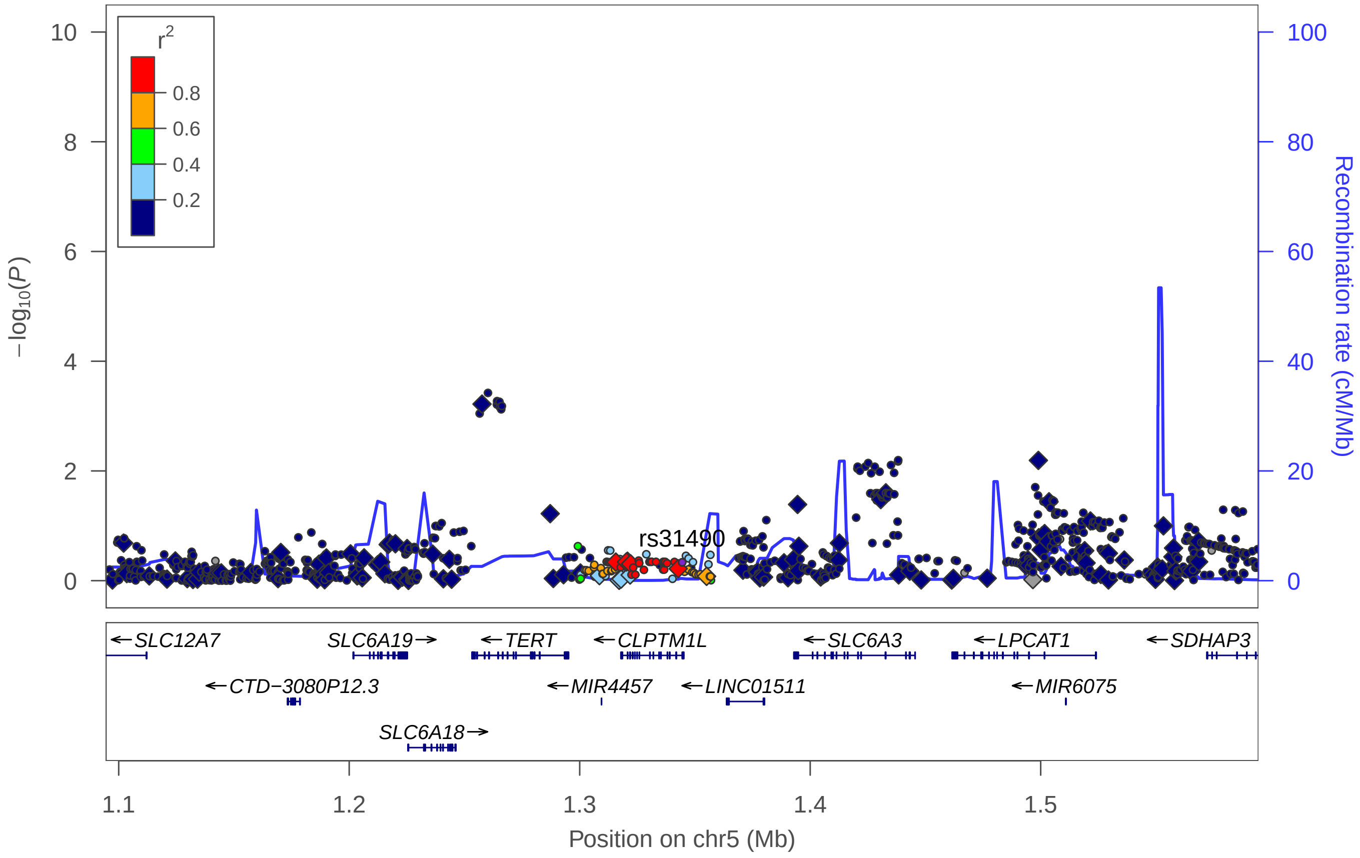

Supplementary Figure 33: **Regional association plot of TTFT (time to first time treatment) for known CLL etiological risk variant rs31490**. For each study, allelic dosage was estimated for the minor allele at each variant position and included in a Cox proportional hazard model to estimate hazard ratio (HR) and 95% confidence interval (CI). Variants were included in the meta-analysis if they had results from all six studies. Study-specific single nucleotide polymorphism (SNP) effects were combined in a fixed effect model using an inverse-variance-weighted method. The upper panel shows SNP coordinates based on genomic build b37/hg19 on the x-axis, and -$\log_{10}$ (P values) on the y-axis. Genotyped and imputed SNPs are represented by diamonds and circles, respectively, and are coloured according to their linkage disequilibrium (pairwise r^2) with the CLL etiological risk variant based on the 1000 Genomes European panel. Reference genes in the region are shown in the lower panel, with arrows indicating transcript direction, dense blocks representing exons and horizontal lines representing introns.

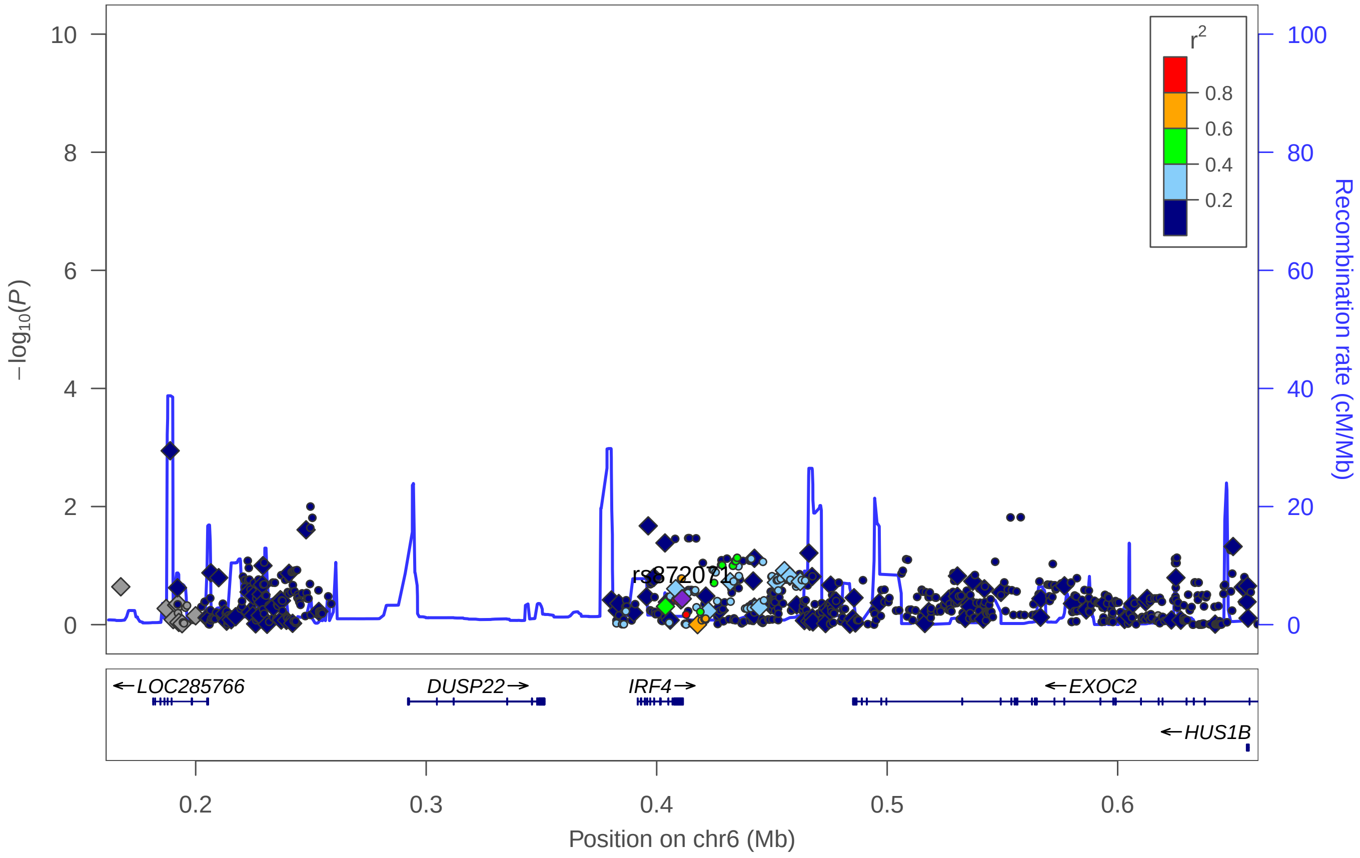

Supplementary Figure 34: **Regional association plot of TTFT (time to first time treatment) for known CLL etiological risk variant rs872071**. For each study, allelic dosage was estimated for the minor allele at each variant position and included in a Cox proportional hazard model to estimate hazard ratio (HR) and 95% confidence interval (CI). Variants were included in the meta-analysis if they had results from all six studies. Study-specific single nucleotide polymorphism (SNP) effects were combined in a fixed effect model using an inverse-variance-weighted method. The upper panel shows SNP coordinates based on genomic build b37/hg19 on the x-axis, and -$\log_{10}$ (P values) on the y-axis. Genotyped and imputed SNPs are represented by diamonds and circles, respectively, and are coloured according to their linkage disequilibrium (pairwise r^2) with the CLL etiological risk variant based on the 1000 Genomes European panel. Reference genes in the region are shown in the lower panel, with arrows indicating transcript direction, dense blocks representing exons and horizontal lines representing introns.

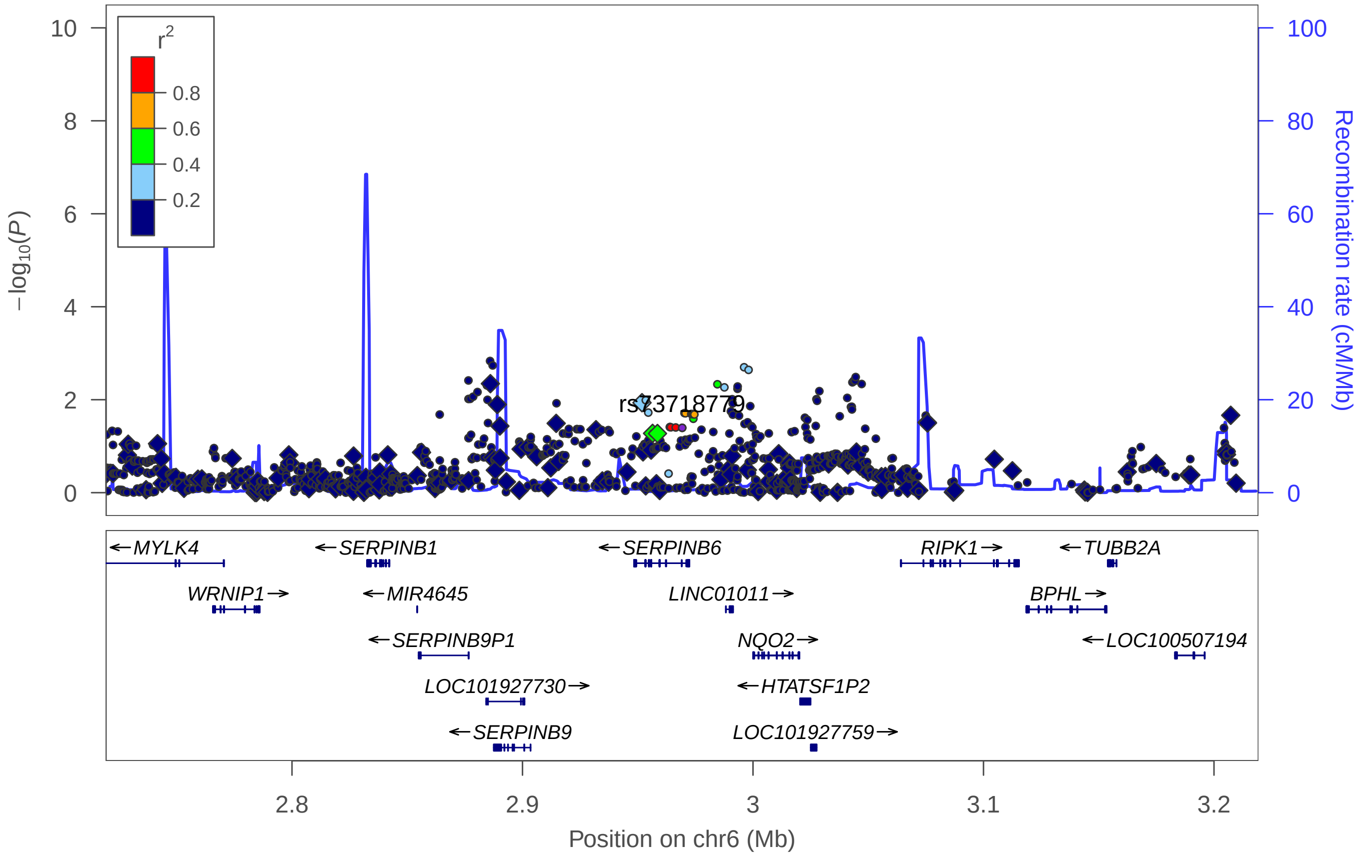

Supplementary Figure 35: **Regional association plot of TTFT (time to first time treatment) for known CLL etiological risk variant rs73718779**. For each study, allelic dosage was estimated for the minor allele at each variant position and included in a Cox proportional hazard model to estimate hazard ratio (HR) and 95% confidence interval (CI). Variants were included in the meta-analysis if they had results from all six studies. Study-specific single nucleotide polymorphism (SNP) effects were combined in a fixed effect model using an inverse-variance-weighted method. The upper panel shows SNP coordinates based on genomic build b37/hg19 on the x-axis, and -$\log_{10}$ (P values) on the y-axis. Genotyped and imputed SNPs are represented by diamonds and circles, respectively, and are coloured according to their linkage disequilibrium (pairwise r^2) with the CLL etiological risk variant based on the 1000 Genomes European panel. Reference genes in the region are shown in the lower panel, with arrows indicating transcript direction, dense blocks representing exons and horizontal lines representing introns.

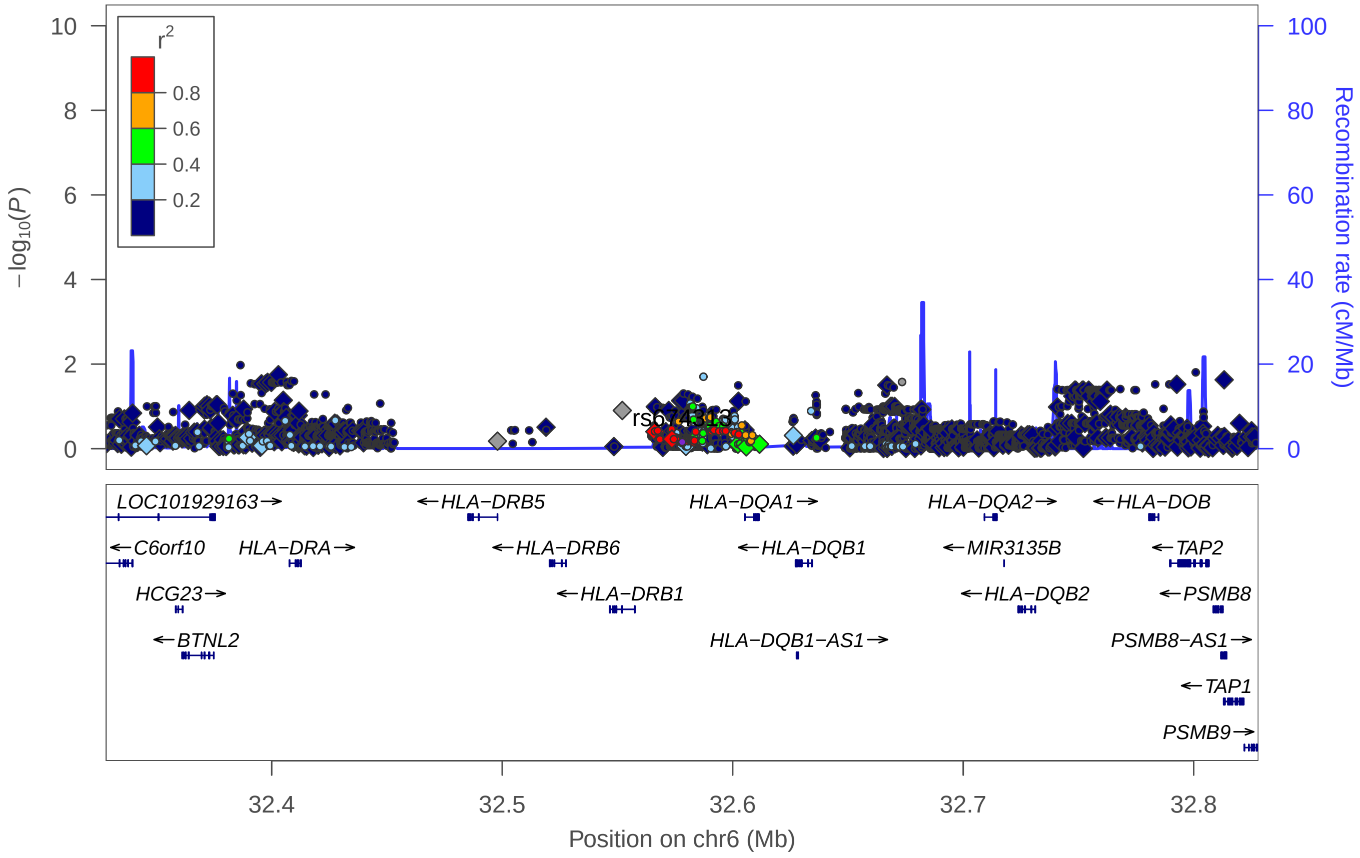

Supplementary Figure 36: **Regional association plot of TTFT (time to first time treatment) for known CLL etiological risk variant rs674313**. For each study, allelic dosage was estimated for the minor allele at each variant position and included in a Cox proportional hazard model to estimate hazard ratio (HR) and 95% confidence interval (CI). Variants were included in the meta-analysis if they had results from all six studies. Study-specific single nucleotide polymorphism (SNP) effects were combined in a fixed effect model using an inverse-variance-weighted method. The upper panel shows SNP coordinates based on genomic build b37/hg19 on the x-axis, and -$\log_{10}$ (P values) on the y-axis. Genotyped and imputed SNPs are represented by diamonds and circles, respectively, and are coloured according to their linkage disequilibrium (pairwise r^2) with the CLL etiological risk variant based on the 1000 Genomes European panel. Reference genes in the region are shown in the lower panel, with arrows indicating transcript direction, dense blocks representing exons and horizontal lines representing introns.

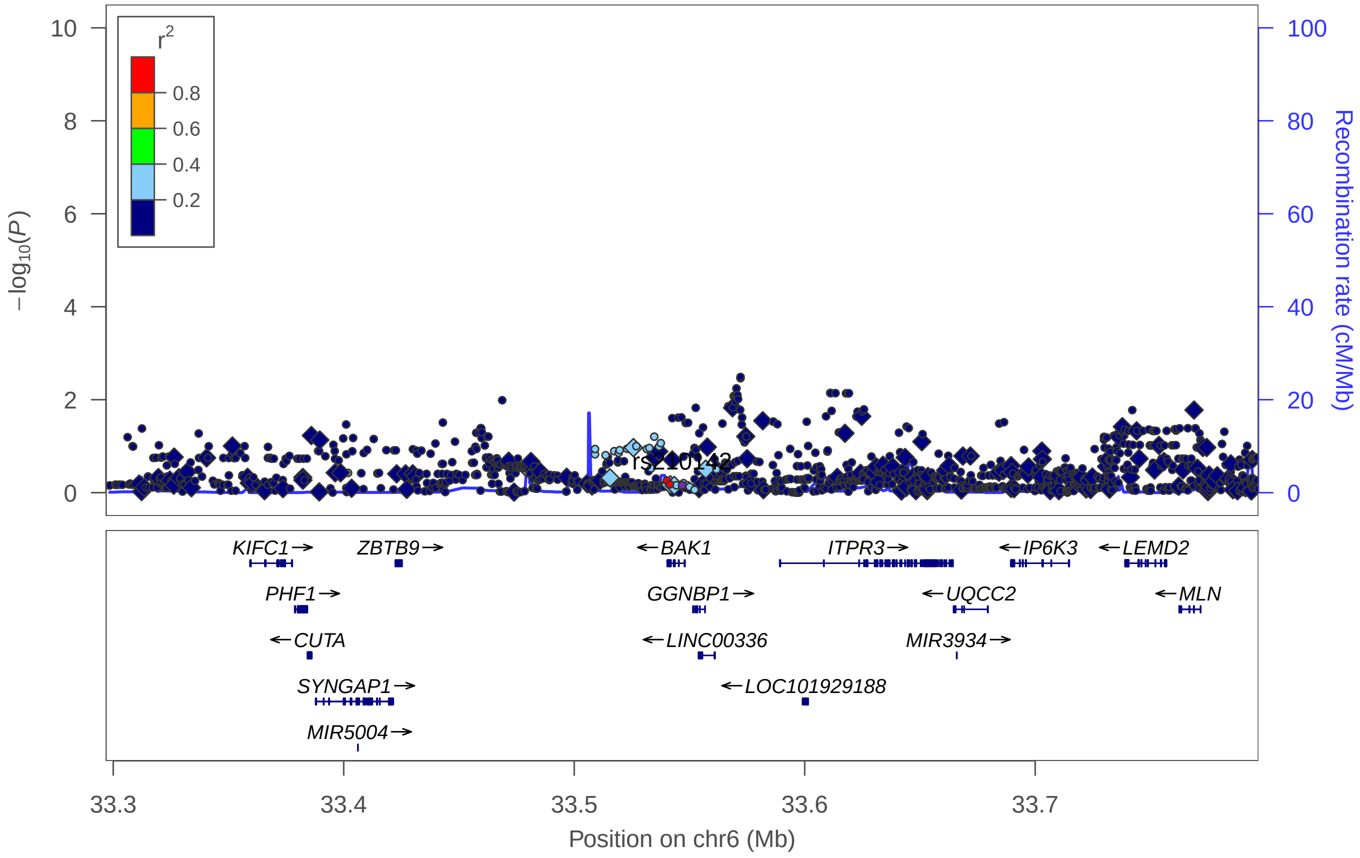

Supplementary Figure 37: **Regional association plot of TTFT (time to first time treatment) for known CLL etiological risk variant rs210142**. For each study, allelic dosage was estimated for the minor allele at each variant position and included in a Cox proportional hazard model to estimate hazard ratio (HR) and 95% confidence interval (CI). Variants were included in the meta-analysis if they had results from all six studies. Study-specific single nucleotide polymorphism (SNP) effects were combined in a fixed effect model using an inverse-variance-weighted method. The upper panel shows SNP coordinates based on genomic build b37/hg19 on the x-axis, and -$\log_{10}$ (P values) on the y-axis. Genotyped and imputed SNPs are represented by diamonds and circles, respectively, and are coloured according to their linkage disequilibrium (pairwise r^2) with the CLL etiological risk variant based on the 1000 Genomes European panel. Reference genes in the region are shown in the lower panel, with arrows indicating transcript direction, dense blocks representing exons and horizontal lines representing introns.

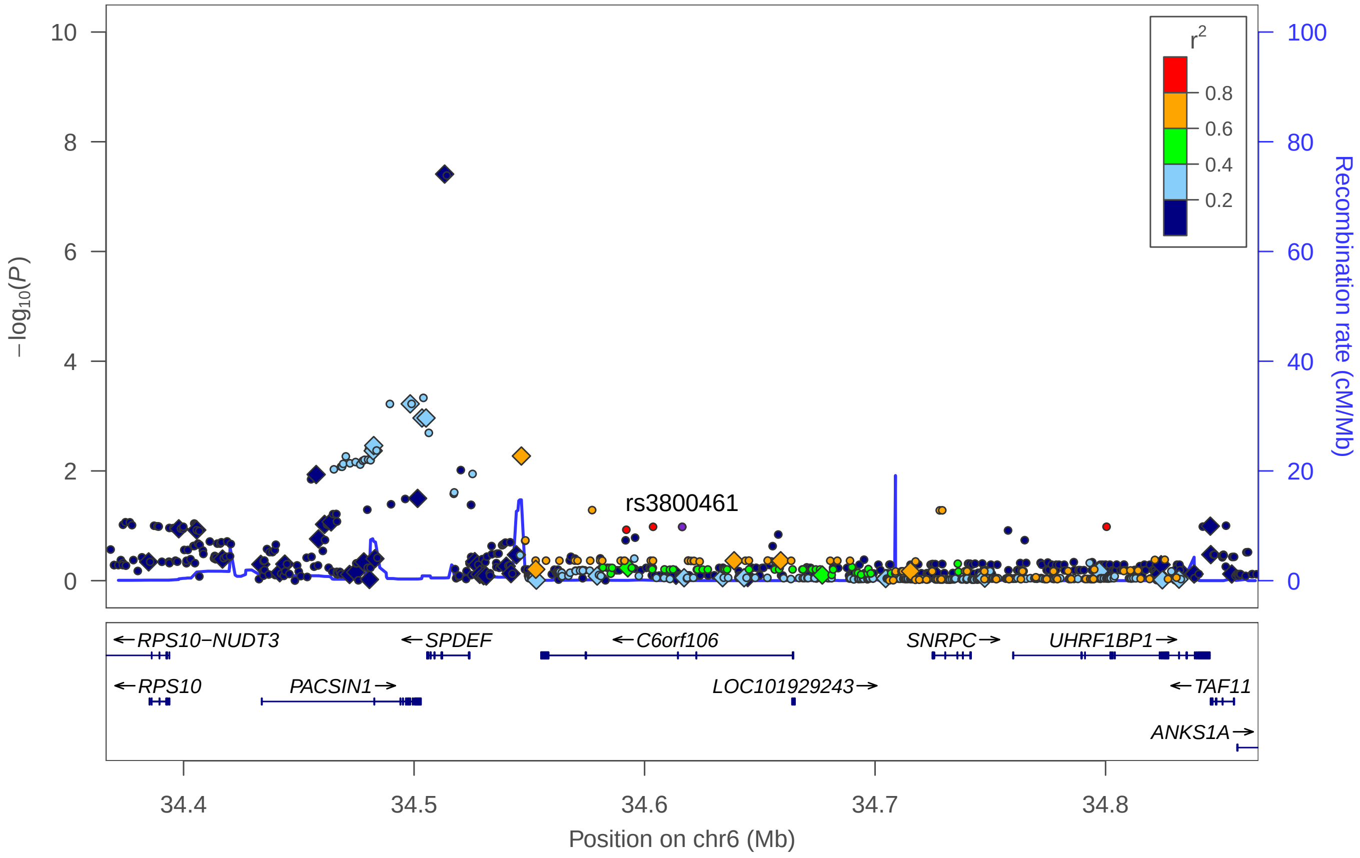

Supplementary Figure 38: **Regional association plot of TTFT (time to first time treatment) for known CLL etiological risk variant rs3800461**. For each study, allelic dosage was estimated for the minor allele at each variant position and included in a Cox proportional hazard model to estimate hazard ratio (HR) and 95% confidence interval (CI). Variants were included in the meta-analysis if they had results from all six studies. Study-specific single nucleotide polymorphism (SNP) effects were combined in a fixed effect model using an inverse-variance-weighted method. The upper panel shows SNP coordinates based on genomic build b37/hg19 on the x-axis, and -$\log_{10}$ (P values) on the y-axis. Genotyped and imputed SNPs are represented by diamonds and circles, respectively, and are coloured according to their linkage disequilibrium (pairwise r^2) with the CLL etiological risk variant based on the 1000 Genomes European panel. Reference genes in the region are shown in the lower panel, with arrows indicating transcript direction, dense blocks representing exons and horizontal lines representing introns.

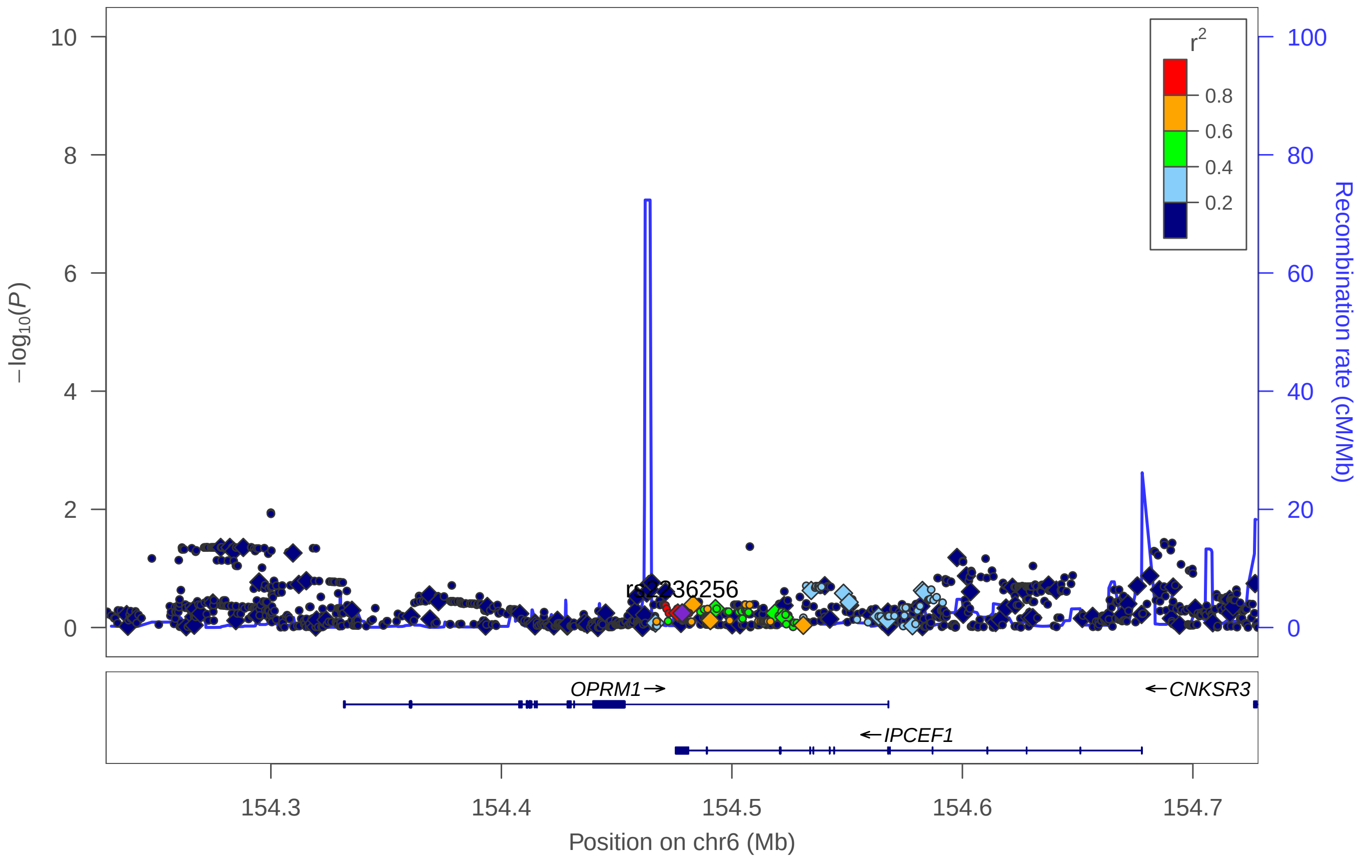

Supplementary Figure 39: **Regional association plot of TTFT (time to first time treatment) for known CLL etiological risk variant rs2236256**. For each study, allelic dosage was estimated for the minor allele at each variant position and included in a Cox proportional hazard model to estimate hazard ratio (HR) and 95% confidence interval (CI). Variants were included in the meta-analysis if they had results from all six studies. Study-specific single nucleotide polymorphism (SNP) effects were combined in a fixed effect model using an inverse-variance-weighted method. The upper panel shows SNP coordinates based on genomic build b37/hg19 on the x-axis, and -$\log_{10}$ (P values) on the y-axis. Genotyped and imputed SNPs are represented by diamonds and circles, respectively, and are coloured according to their linkage disequilibrium (pairwise r^2) with the CLL etiological risk variant based on the 1000 Genomes European panel. Reference genes in the region are shown in the lower panel, with arrows indicating transcript direction, dense blocks representing exons and horizontal lines representing introns.

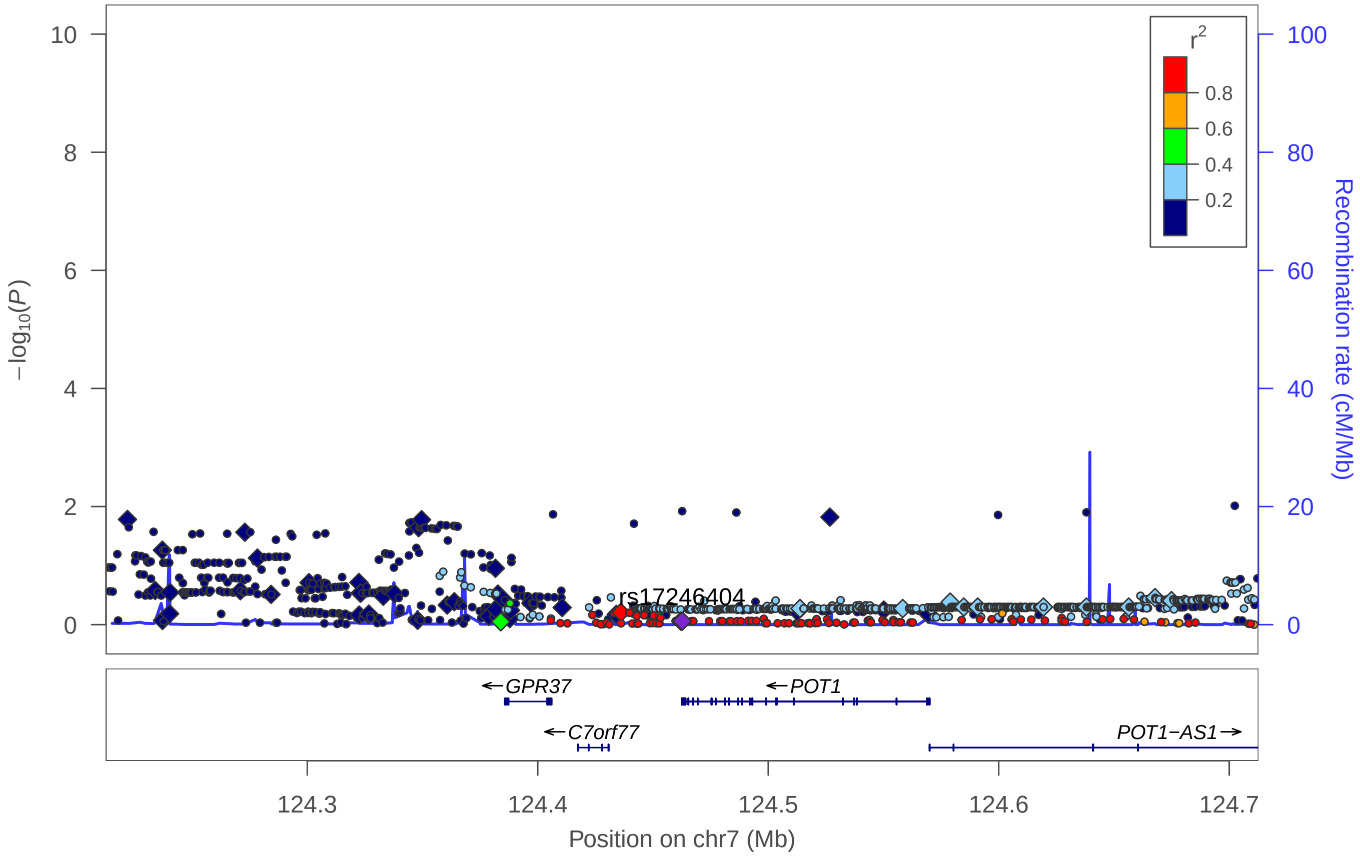

Supplementary Figure 40: **Regional association plot of TTFT (time to first time treatment) for known CLL etiological risk variant rs17246404**. For each study, allelic dosage was estimated for the minor allele at each variant position and included in a Cox proportional hazard model to estimate hazard ratio (HR) and 95% confidence interval (CI). Variants were included in the meta-analysis if they had results from all six studies. Study-specific single nucleotide polymorphism (SNP) effects were combined in a fixed effect model using an inverse-variance-weighted method. The upper panel shows SNP coordinates based on genomic build b37/hg19 on the x-axis, and -log$_{10}$ (P values) on the y-axis. Genotyped and imputed SNPs are represented by diamonds and circles, respectively, and are coloured according to their linkage disequilibrium (pairwise r^2) with the CLL etiological risk variant based on the 1000 Genomes European panel. Reference genes in the region are shown in the lower panel, with arrows indicating transcript direction, dense blocks representing exons and horizontal lines representing introns.

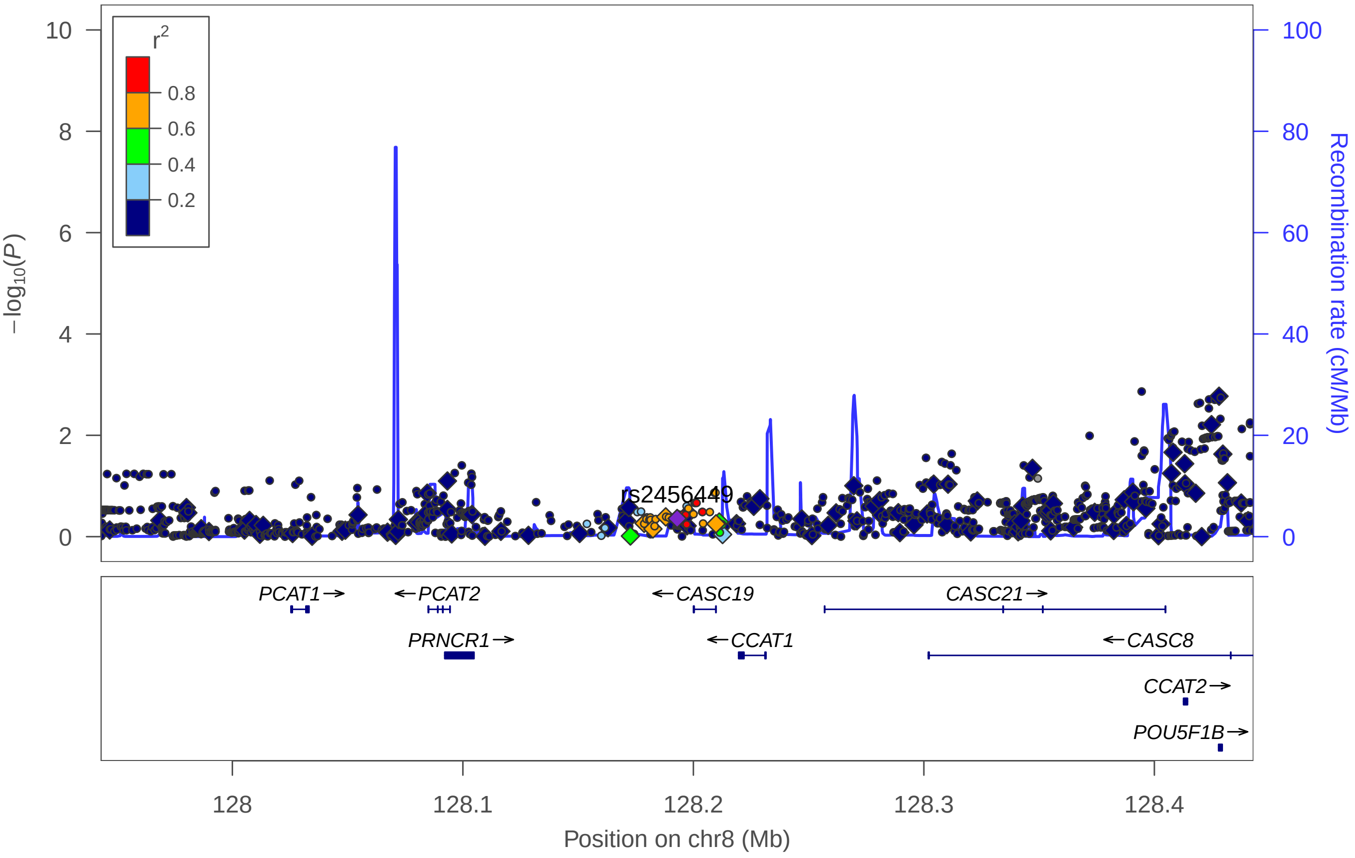

Supplementary Figure 41: **Regional association plot of TTFT (time to first time treatment) for known CLL etiological risk variant rs2456449**. For each study, allelic dosage was estimated for the minor allele at each variant position and included in a Cox proportional hazard model to estimate hazard ratio (HR) and 95% confidence interval (CI). Variants were included in the meta-analysis if they had results from all six studies. Study-specific single nucleotide polymorphism (SNP) effects were combined in a fixed effect model using an inverse-variance-weighted method. The upper panel shows SNP coordinates based on genomic build b37/hg19 on the x-axis, and -$\log_{10}$ (P values) on the y-axis. Genotyped and imputed SNPs are represented by diamonds and circles, respectively, and are coloured according to their linkage disequilibrium (pairwise r^2) with the CLL etiological risk variant based on the 1000 Genomes European panel. Reference genes in the region are shown in the lower panel, with arrows indicating transcript direction, dense blocks representing exons and horizontal lines representing introns.

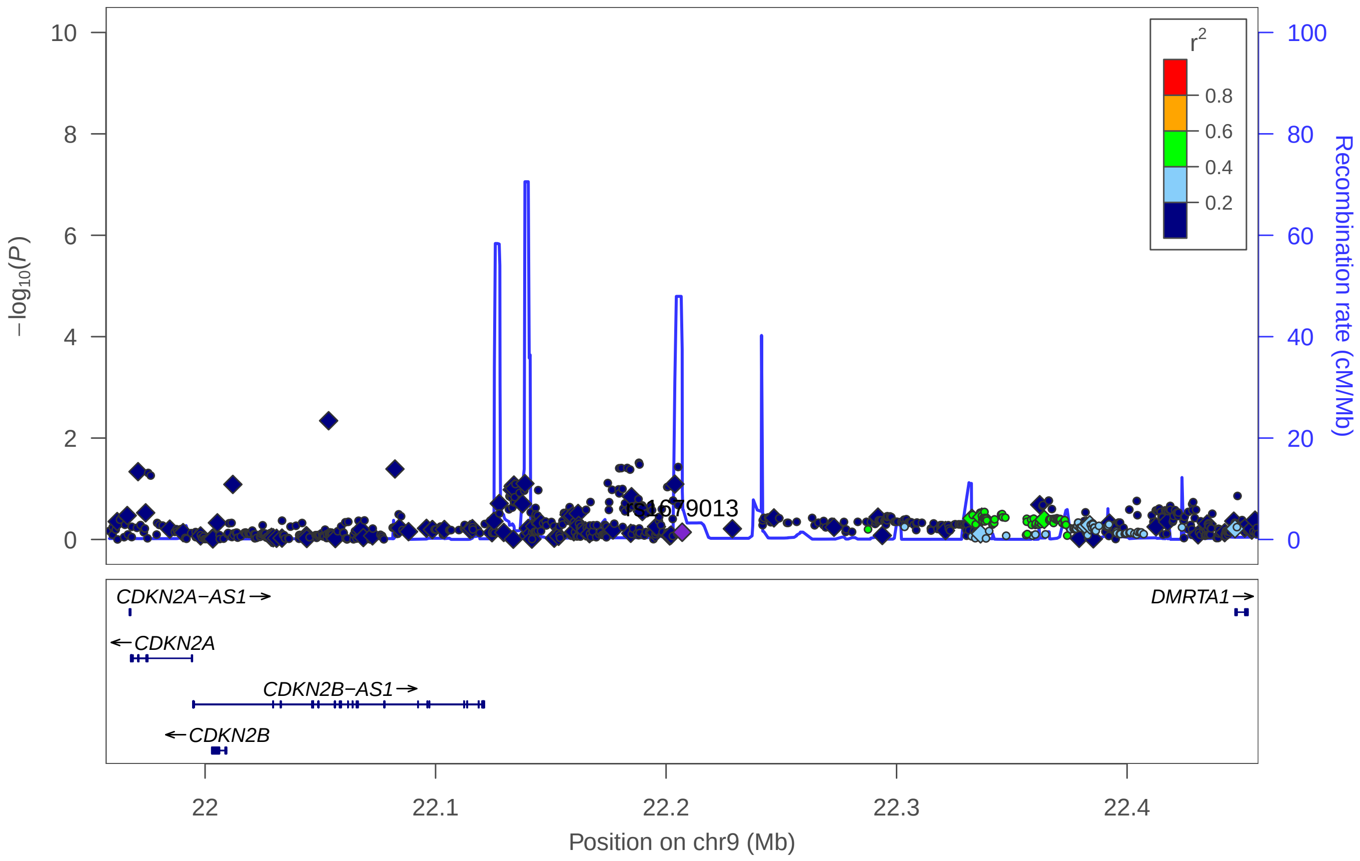

Supplementary Figure 42: **Regional association plot of TTFT (time to first time treatment) for known CLL etiological risk variant rs1679013**. For each study, allelic dosage was estimated for the minor allele at each variant position and included in a Cox proportional hazard model to estimate hazard ratio (HR) and 95% confidence interval (CI). Variants were included in the meta-analysis if they had results from all six studies. Study-specific single nucleotide polymorphism (SNP) effects were combined in a fixed effect model using an inverse-variance-weighted method. The upper panel shows SNP coordinates based on genomic build b37/hg19 on the x-axis, and -$\log_{10}$ (P values) on the y-axis. Genotyped and imputed SNPs are represented by diamonds and circles, respectively, and are coloured according to their linkage disequilibrium (pairwise r^2) with the CLL etiological risk variant based on the 1000 Genomes European panel. Reference genes in the region are shown in the lower panel, with arrows indicating transcript direction, dense blocks representing exons and horizontal lines representing introns.

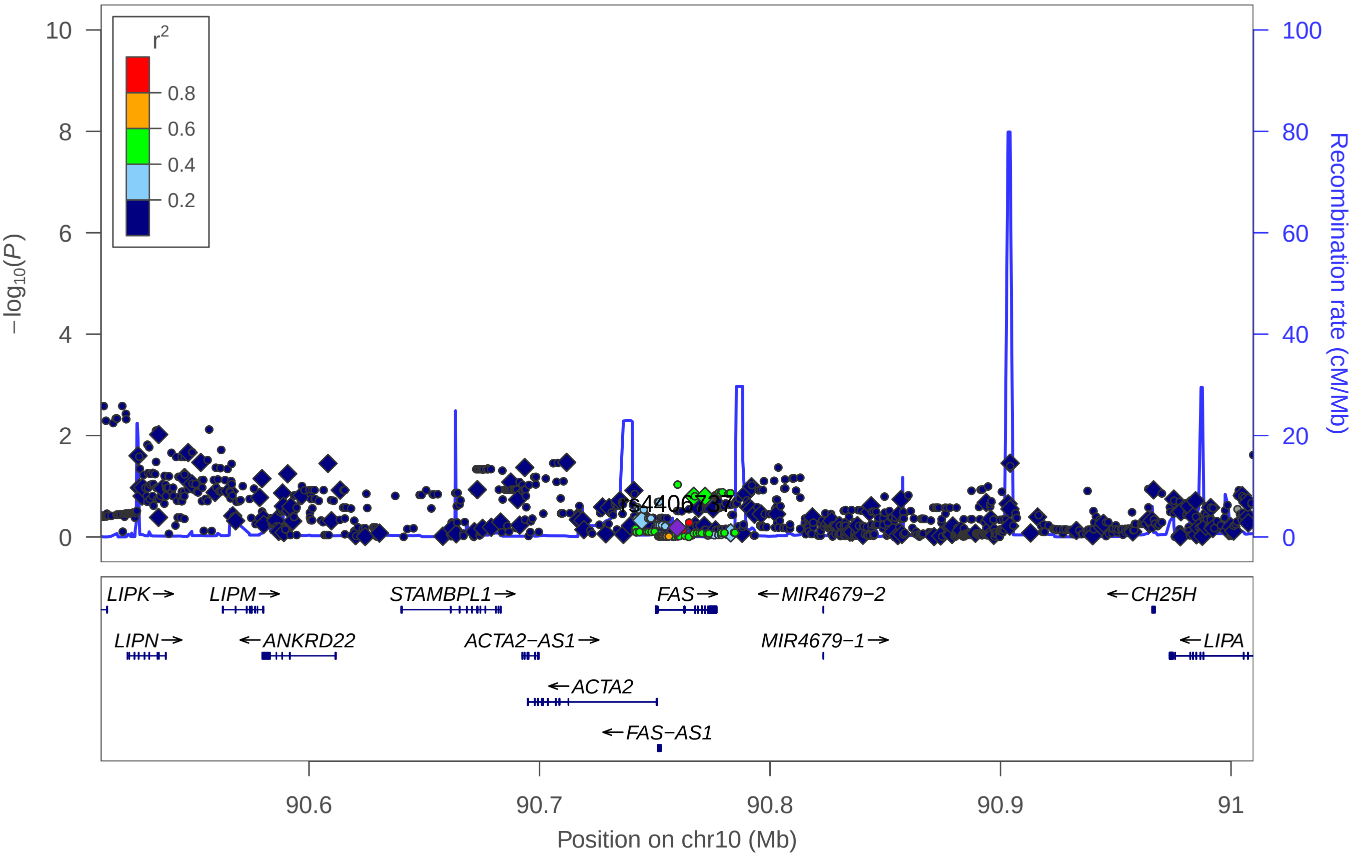

Supplementary Figure 43: **Regional association plot of TTFT (time to first time treatment) for known CLL etiological risk variant rs4406737**. For each study, allelic dosage was estimated for the minor allele at each variant position and included in a Cox proportional hazard model to estimate hazard ratio (HR) and 95% confidence interval (CI). Variants were included in the meta-analysis if they had results from all six studies. Study-specific single nucleotide polymorphism (SNP) effects were combined in a fixed effect model using an inverse-variance-weighted method. The upper panel shows SNP coordinates based on genomic build b37/hg19 on the x-axis, and -$\log_{10}$ (P values) on the y-axis. Genotyped and imputed SNPs are represented by diamonds and circles, respectively, and are coloured according to their linkage disequilibrium (pairwise r^2) with the CLL etiological risk variant based on the 1000 Genomes European panel. Reference genes in the region are shown in the lower panel, with arrows indicating transcript direction, dense blocks representing exons and horizontal lines representing introns.

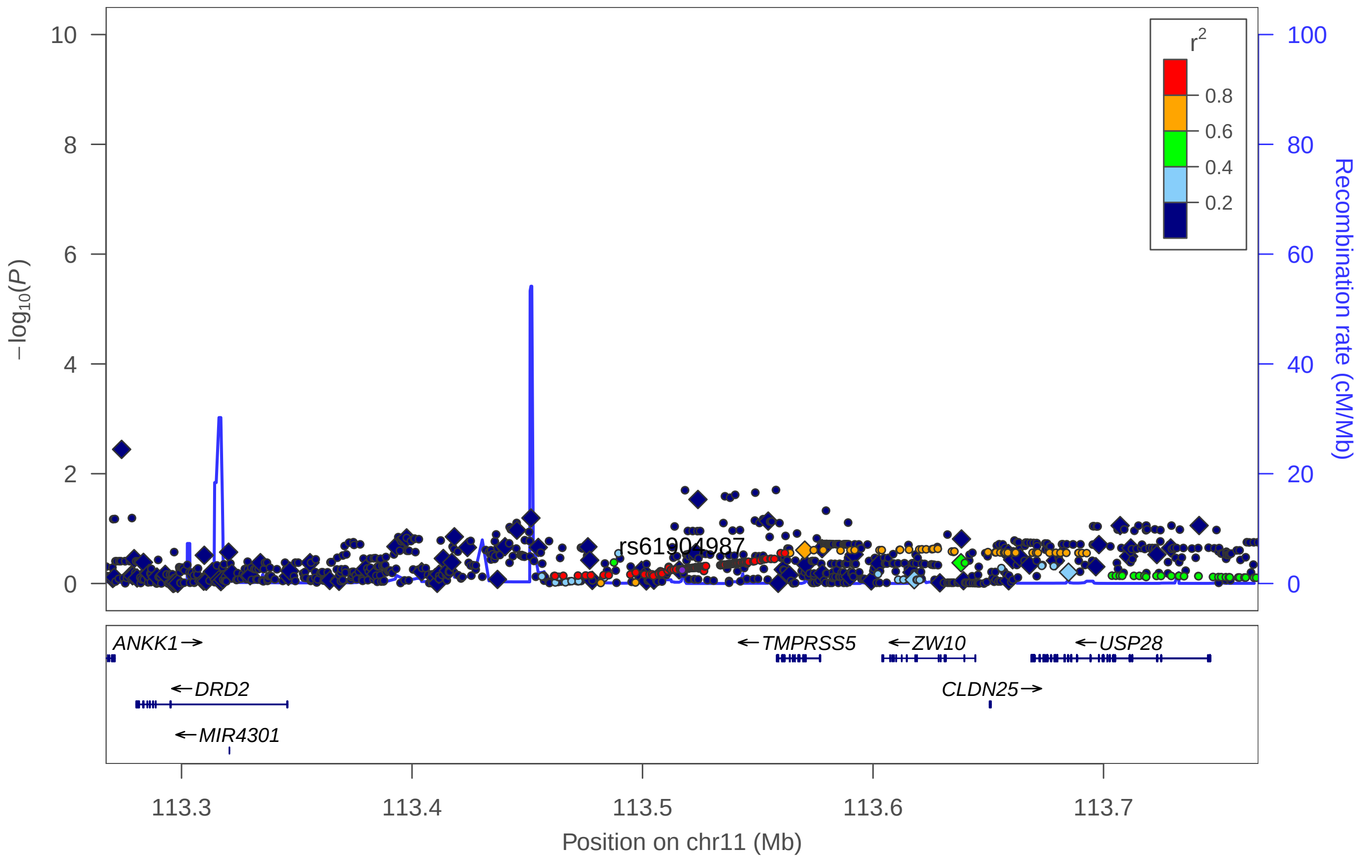

Supplementary Figure 44: **Regional association plot of TTFT (time to first time treatment) for known CLL etiological risk variant rs61904987**. For each study, allelic dosage was estimated for the minor allele at each variant position and included in a Cox proportional hazard model to estimate hazard ratio (HR) and 95% confidence interval (CI). Variants were included in the meta-analysis if they had results from all six studies. Study-specific single nucleotide polymorphism (SNP) effects were combined in a fixed effect model using an inverse-variance-weighted method. The upper panel shows SNP coordinates based on genomic build b37/hg19 on the x-axis, and -$\log_{10}$ (P values) on the y-axis. Genotyped and imputed SNPs are represented by diamonds and circles, respectively, and are coloured according to their linkage disequilibrium (pairwise r^2) with the CLL etiological risk variant based on the 1000 Genomes European panel. Reference genes in the region are shown in the lower panel, with arrows indicating transcript direction, dense blocks representing exons and horizontal lines representing introns.

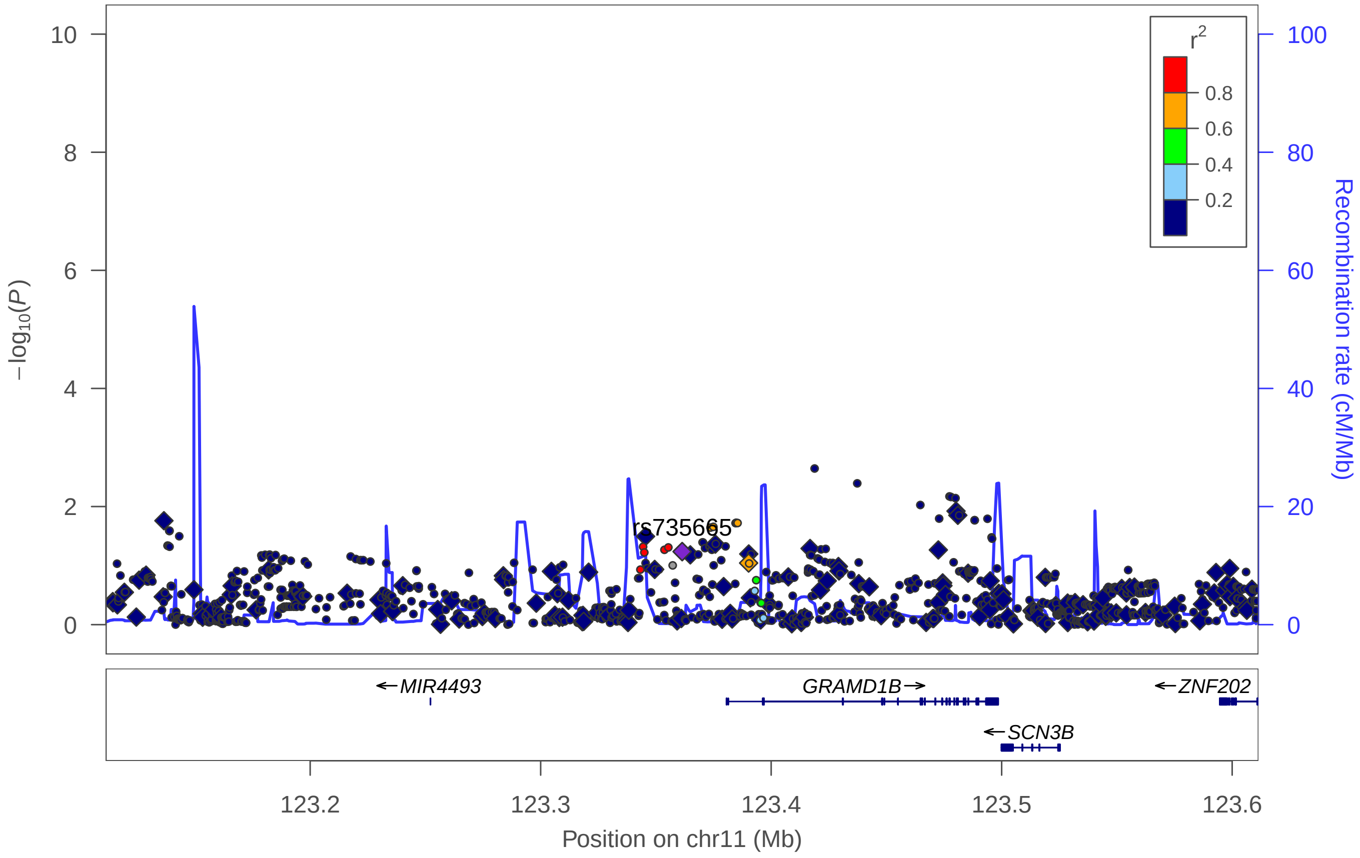

Supplementary Figure 45: **Regional association plot of TTFT (time to first time treatment) for known CLL etiological risk variant rs735665**. For each study, allelic dosage was estimated for the minor allele at each variant position and included in a Cox proportional hazard model to estimate hazard ratio (HR) and 95% confidence interval (CI). Variants were included in the meta-analysis if they had results from all six studies. Study-specific single nucleotide polymorphism (SNP) effects were combined in a fixed effect model using an inverse-variance-weighted method. The upper panel shows SNP coordinates based on genomic build b37/hg19 on the x-axis, and -$\log_{10}$ (P values) on the y-axis. Genotyped and imputed SNPs are represented by diamonds and circles, respectively, and are coloured according to their linkage disequilibrium (pairwise r^2) with the CLL etiological risk variant based on the 1000 Genomes European panel. Reference genes in the region are shown in the lower panel, with arrows indicating transcript direction, dense blocks representing exons and horizontal lines representing introns.

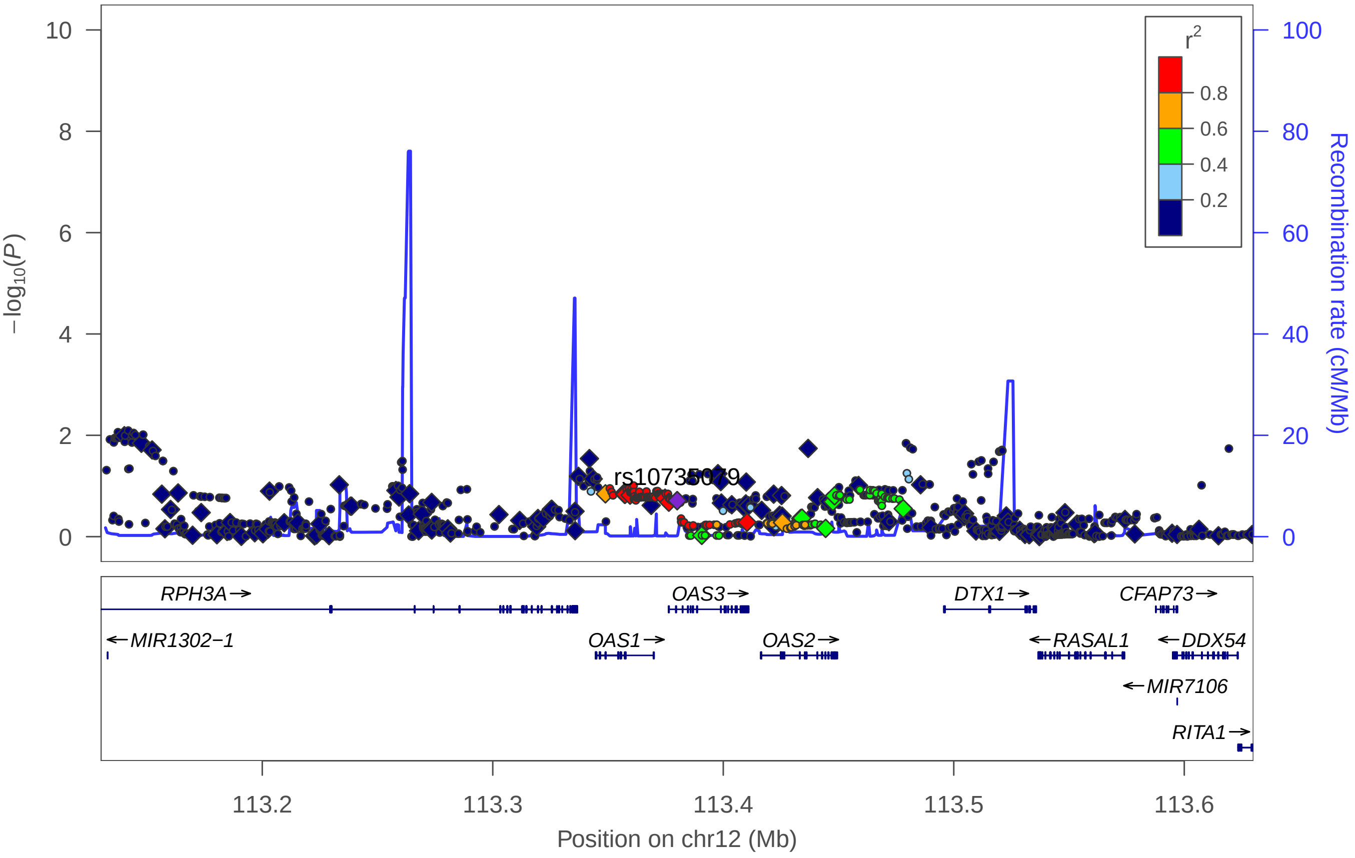

Supplementary Figure 46: **Regional association plot of TTFT (time to first time treatment) for known CLL etiological risk variant rs10735079**. For each study, allelic dosage was estimated for the minor allele at each variant position and included in a Cox proportional hazard model to estimate hazard ratio (HR) and 95% confidence interval (CI). Variants were included in the meta-analysis if they had results from all six studies. Study-specific single nucleotide polymorphism (SNP) effects were combined in a fixed effect model using an inverse-variance-weighted method. The upper panel shows SNP coordinates based on genomic build b37/hg19 on the x-axis, and -$\log_{10}$ (P values) on the y-axis. Genotyped and imputed SNPs are represented by diamonds and circles, respectively, and are coloured according to their linkage disequilibrium (pairwise r^2) with the CLL etiological risk variant based on the 1000 Genomes European panel. Reference genes in the region are shown in the lower panel, with arrows indicating transcript direction, dense blocks representing exons and horizontal lines representing introns.

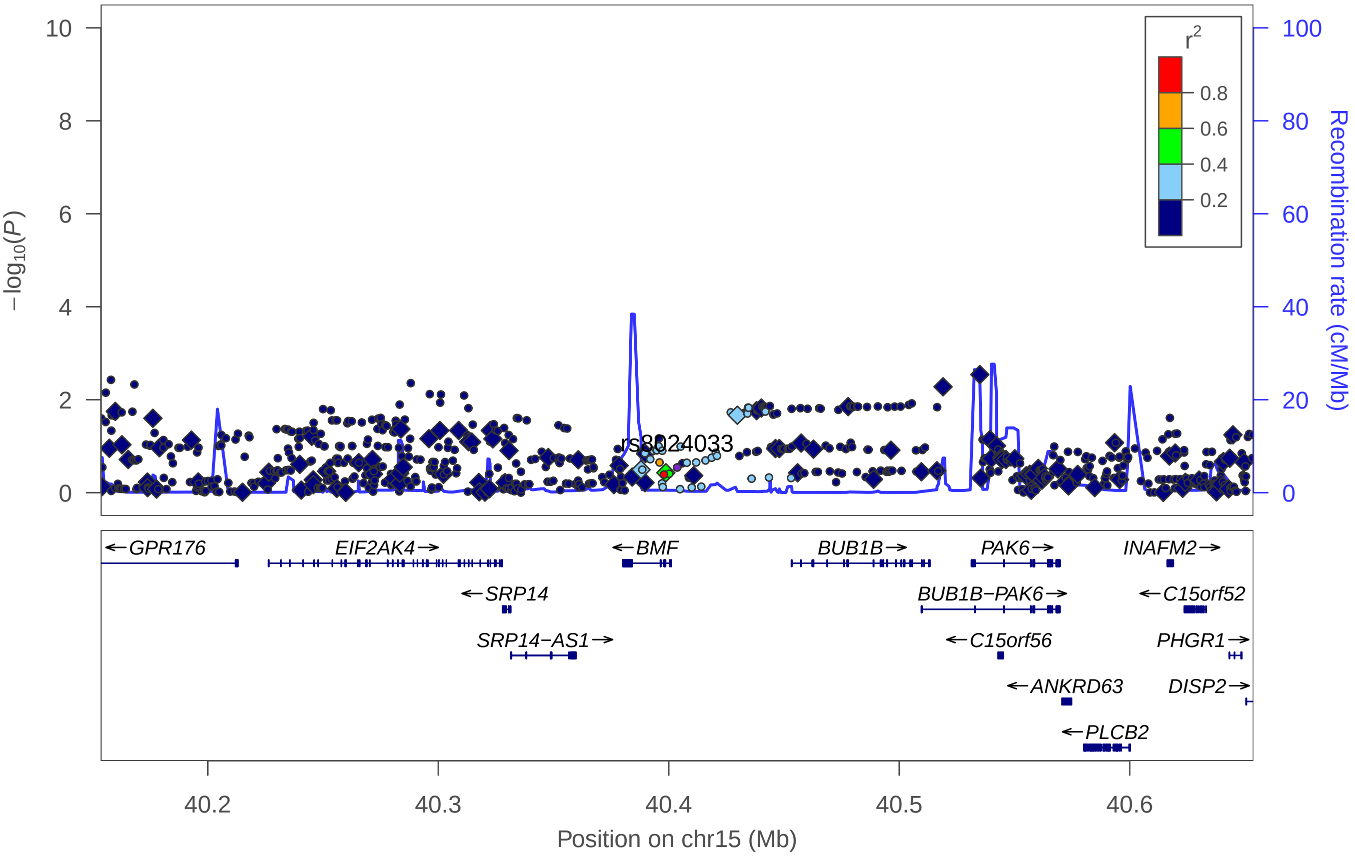

Supplementary Figure 47: **Regional association plot of TTFT (time to first time treatment) for known CLL etiological risk variant rs8024033**. For each study, allelic dosage was estimated for the minor allele at each variant position and included in a Cox proportional hazard model to estimate hazard ratio (HR) and 95% confidence interval (CI). Variants were included in the meta-analysis if they had results from all six studies. Study-specific single nucleotide polymorphism (SNP) effects were combined in a fixed effect model using an inverse-variance-weighted method. The upper panel shows SNP coordinates based on genomic build b37/hg19 on the x-axis, and -$\log_{10}$ (P values) on the y-axis. Genotyped and imputed SNPs are represented by diamonds and circles, respectively, and are coloured according to their linkage disequilibrium (pairwise r^2) with the CLL etiological risk variant based on the 1000 Genomes European panel. Reference genes in the region are shown in the lower panel, with arrows indicating transcript direction, dense blocks representing exons and horizontal lines representing introns.

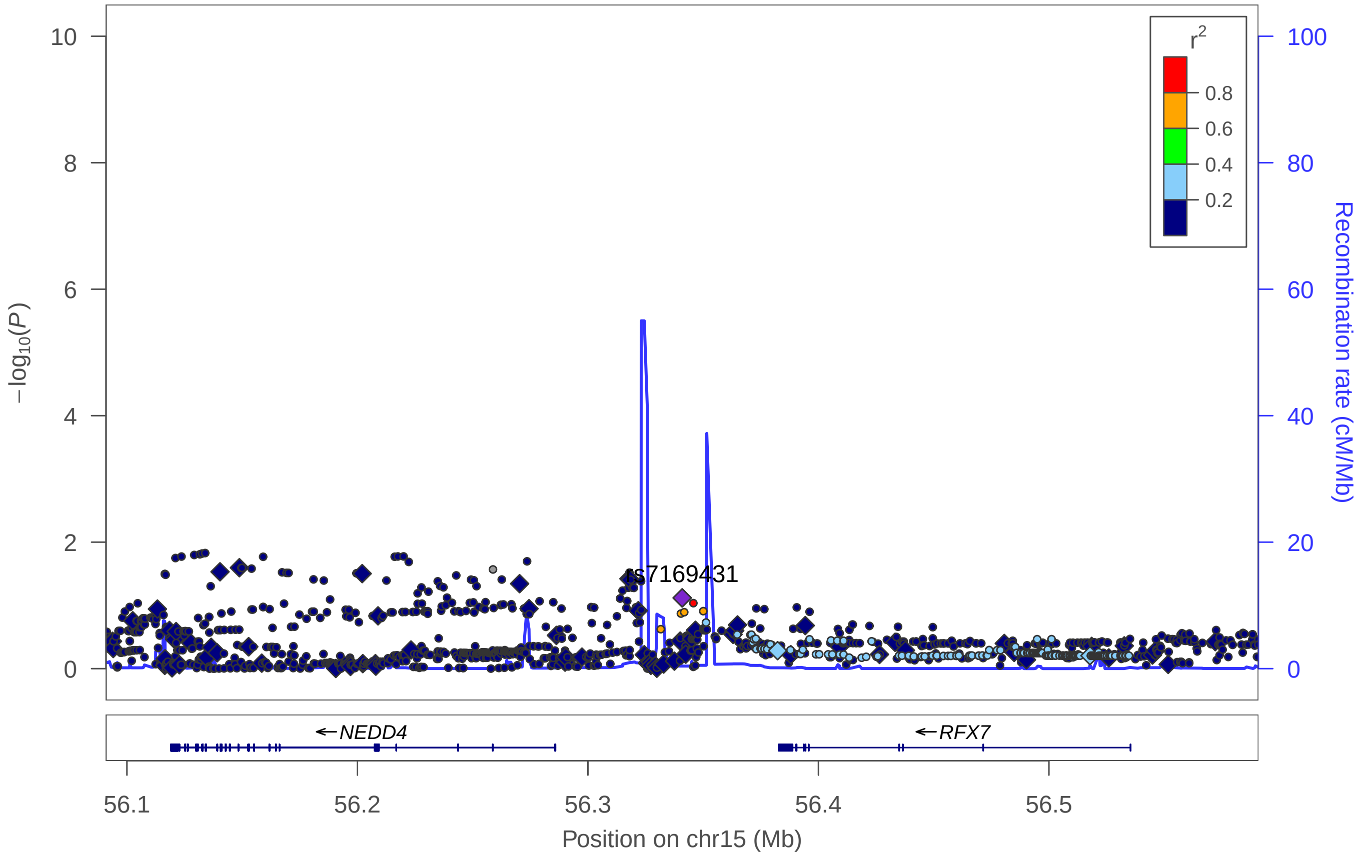

Supplementary Figure 48: **Regional association plot of TTFT (time to first time treatment) for known CLL etiological risk variant rs7169431**. For each study, allelic dosage was estimated for the minor allele at each variant position and included in a Cox proportional hazard model to estimate hazard ratio (HR) and 95% confidence interval (CI). Variants were included in the meta-analysis if they had results from all six studies. Study-specific single nucleotide polymorphism (SNP) effects were combined in a fixed effect model using an inverse-variance-weighted method. The upper panel shows SNP coordinates based on genomic build b37/hg19 on the x-axis, and -$\log_{10}$ (P values) on the y-axis. Genotyped and imputed SNPs are represented by diamonds and circles, respectively, and are coloured according to their linkage disequilibrium (pairwise r^2) with the CLL etiological risk variant based on the 1000 Genomes European panel. Reference genes in the region are shown in the lower panel, with arrows indicating transcript direction, dense blocks representing exons and horizontal lines representing introns.

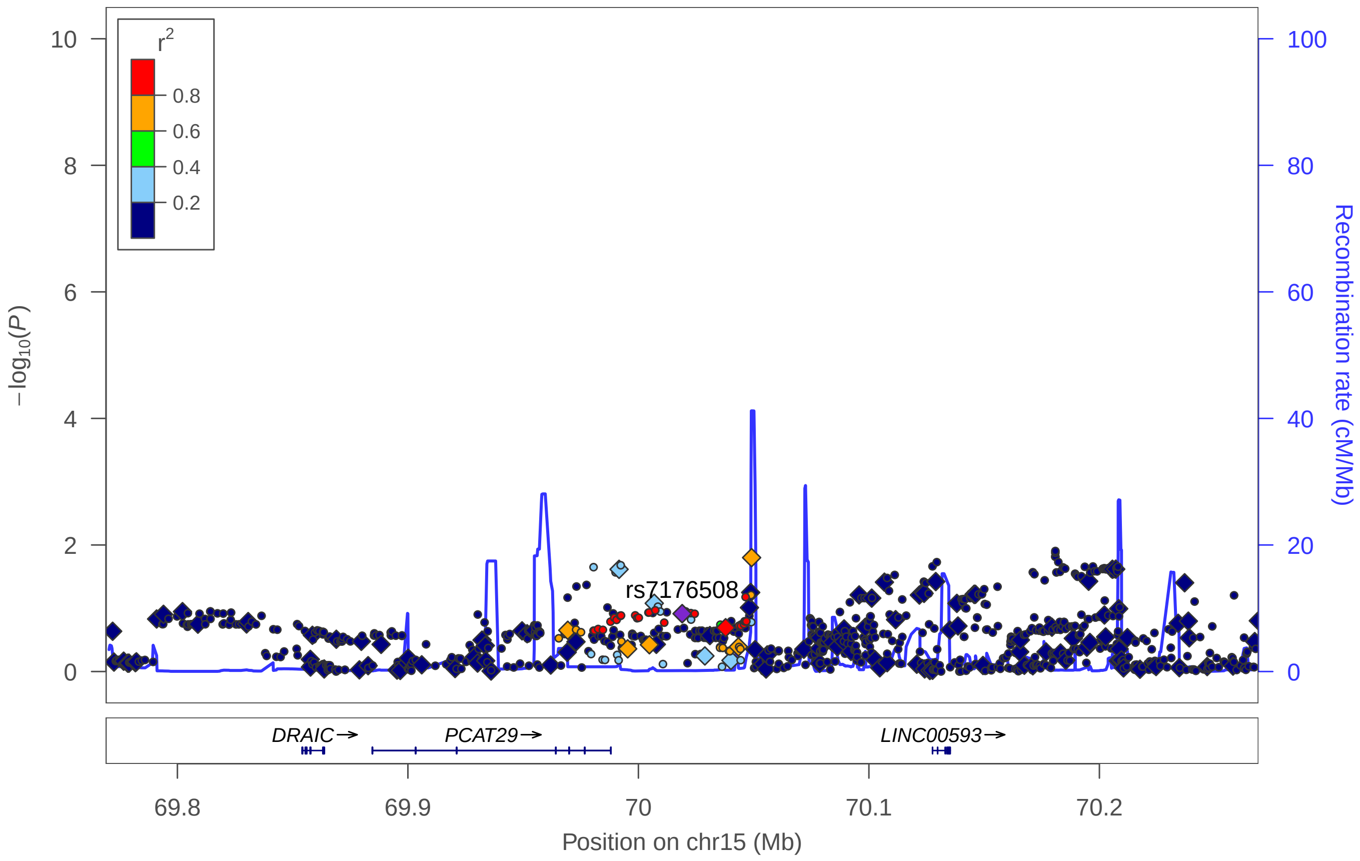

Supplementary Figure 49: **Regional association plot of TTFT (time to first time treatment) for known CLL etiological risk variant rs7176508**. For each study, allelic dosage was estimated for the minor allele at each variant position and included in a Cox proportional hazard model to estimate hazard ratio (HR) and 95% confidence interval (CI). Variants were included in the meta-analysis if they had results from all six studies. Study-specific single nucleotide polymorphism (SNP) effects were combined in a fixed effect model using an inverse-variance-weighted method. The upper panel shows SNP coordinates based on genomic build b37/hg19 on the x-axis, and -$\log_{10}$ (P values) on the y-axis. Genotyped and imputed SNPs are represented by diamonds and circles, respectively, and are coloured according to their linkage disequilibrium (pairwise r^2) with the CLL etiological risk variant based on the 1000 Genomes European panel. Reference genes in the region are shown in the lower panel, with arrows indicating transcript direction, dense blocks representing exons and horizontal lines representing introns.

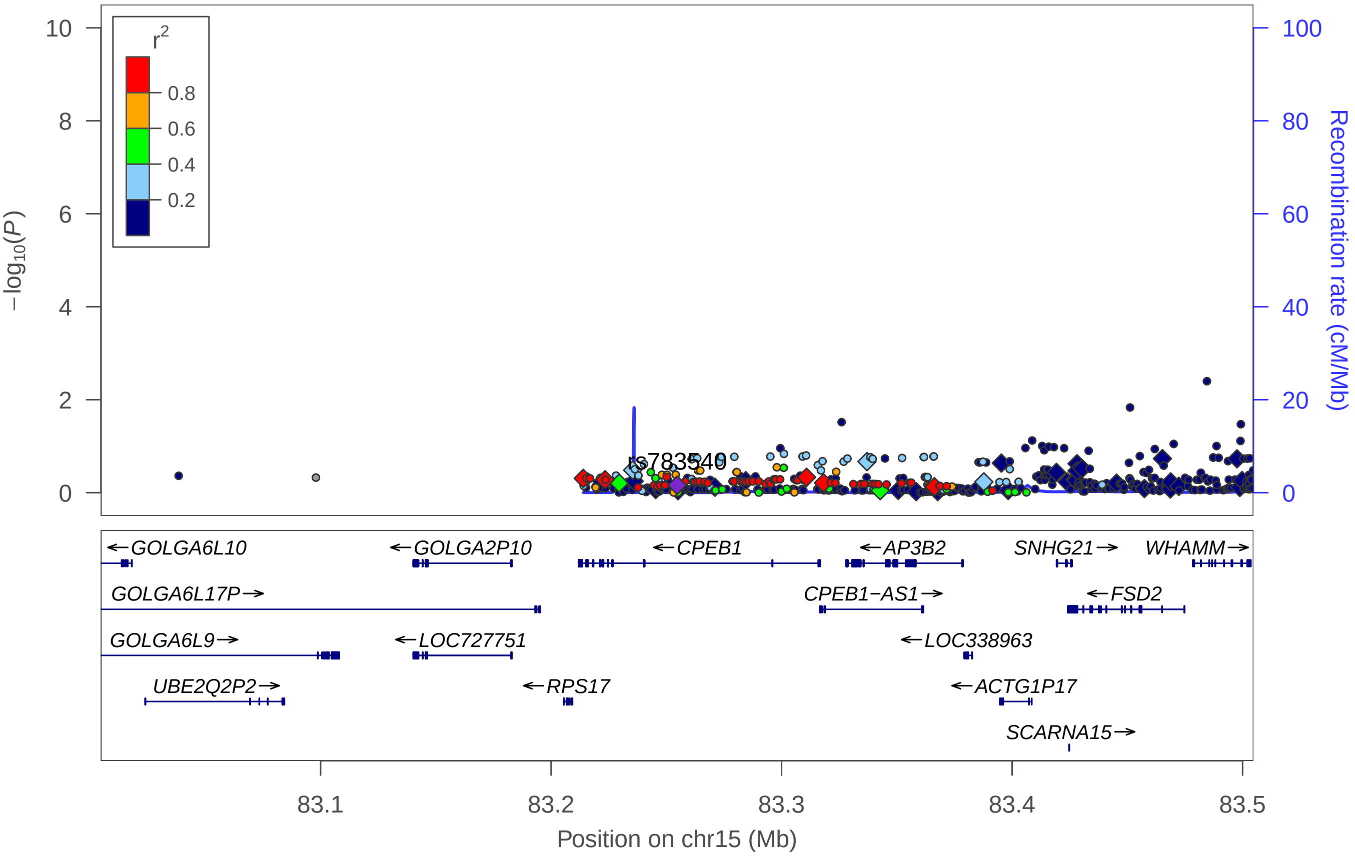

Supplementary Figure 50: **Regional association plot of TTFT (time to first time treatment) for known CLL etiological risk variant rs783540**. For each study, allelic dosage was estimated for the minor allele at each variant position and included in a Cox proportional hazard model to estimate hazard ratio (HR) and 95% confidence interval (CI). Variants were included in the meta-analysis if they had results from all six studies. Study-specific single nucleotide polymorphism (SNP) effects were combined in a fixed effect model using an inverse-variance-weighted method. The upper panel shows SNP coordinates based on genomic build b37/hg19 on the x-axis, and -$\log_{10}$ (P values) on the y-axis. Genotyped and imputed SNPs are represented by diamonds and circles, respectively, and are coloured according to their linkage disequilibrium (pairwise r^2) with the CLL etiological risk variant based on the 1000 Genomes European panel. Reference genes in the region are shown in the lower panel, with arrows indicating transcript direction, dense blocks representing exons and horizontal lines representing introns.

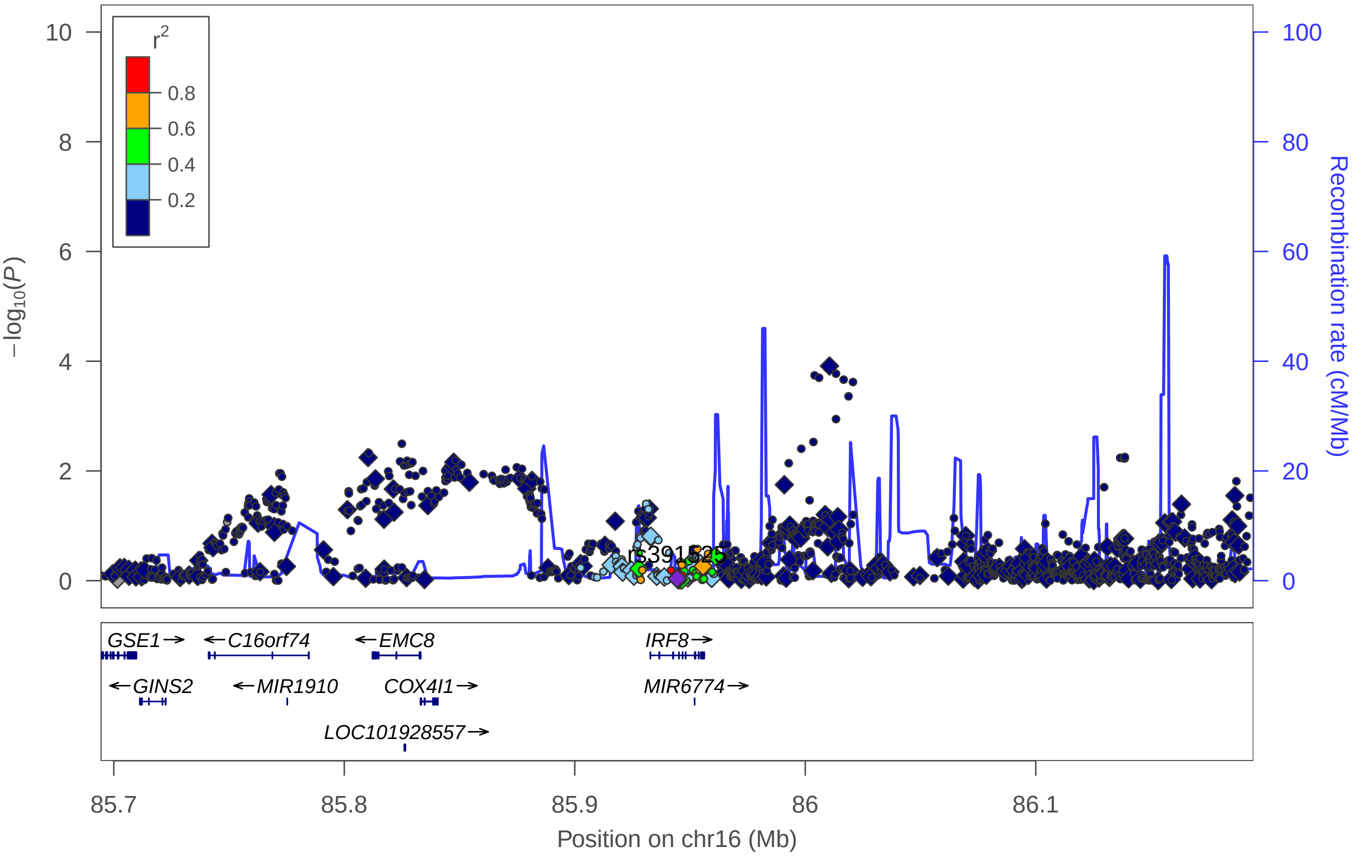

Supplementary Figure 51: **Regional association plot of TTFT (time to first time treatment) for known CLL etiological risk variant rs391525**. For each study, allelic dosage was estimated for the minor allele at each variant position and included in a Cox proportional hazard model to estimate hazard ratio (HR) and 95% confidence interval (CI). Variants were included in the meta-analysis if they had results from all six studies. Study-specific single nucleotide polymorphism (SNP) effects were combined in a fixed effect model using an inverse-variance-weighted method. The upper panel shows SNP coordinates based on genomic build b37/hg19 on the x-axis, and -$\log_{10}$ (P values) on the y-axis. Genotyped and imputed SNPs are represented by diamonds and circles, respectively, and are coloured according to their linkage disequilibrium (pairwise r^2) with the CLL etiological risk variant based on the 1000 Genomes European panel. Reference genes in the region are shown in the lower panel, with arrows indicating transcript direction, dense blocks representing exons and horizontal lines representing introns.

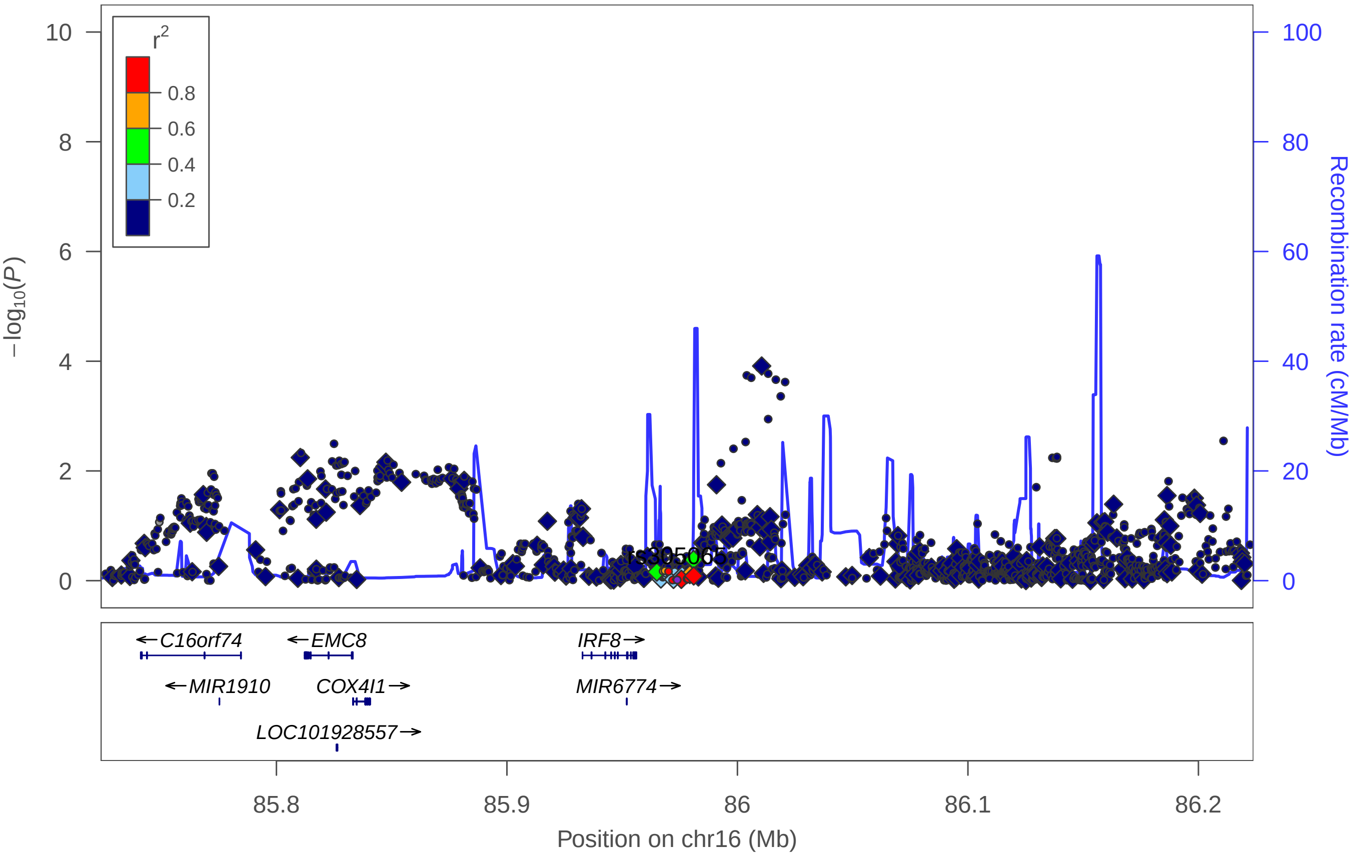

Supplementary Figure 52: **Regional association plot of TTFT (time to first time treatment) for known CLL etiological risk variant rs305065**. For each study, allelic dosage was estimated for the minor allele at each variant position and included in a Cox proportional hazard model to estimate hazard ratio (HR) and 95% confidence interval (CI). Variants were included in the meta-analysis if they had results from all six studies. Study-specific single nucleotide polymorphism (SNP) effects were combined in a fixed effect model using an inverse-variance-weighted method. The upper panel shows SNP coordinates based on genomic build b37/hg19 on the x-axis, and -$\log_{10}$ (P values) on the y-axis. Genotyped and imputed SNPs are represented by diamonds and circles, respectively, and are coloured according to their linkage disequilibrium (pairwise r^2) with the CLL etiological risk variant based on the 1000 Genomes European panel. Reference genes in the region are shown in the lower panel, with arrows indicating transcript direction, dense blocks representing exons and horizontal lines representing introns.

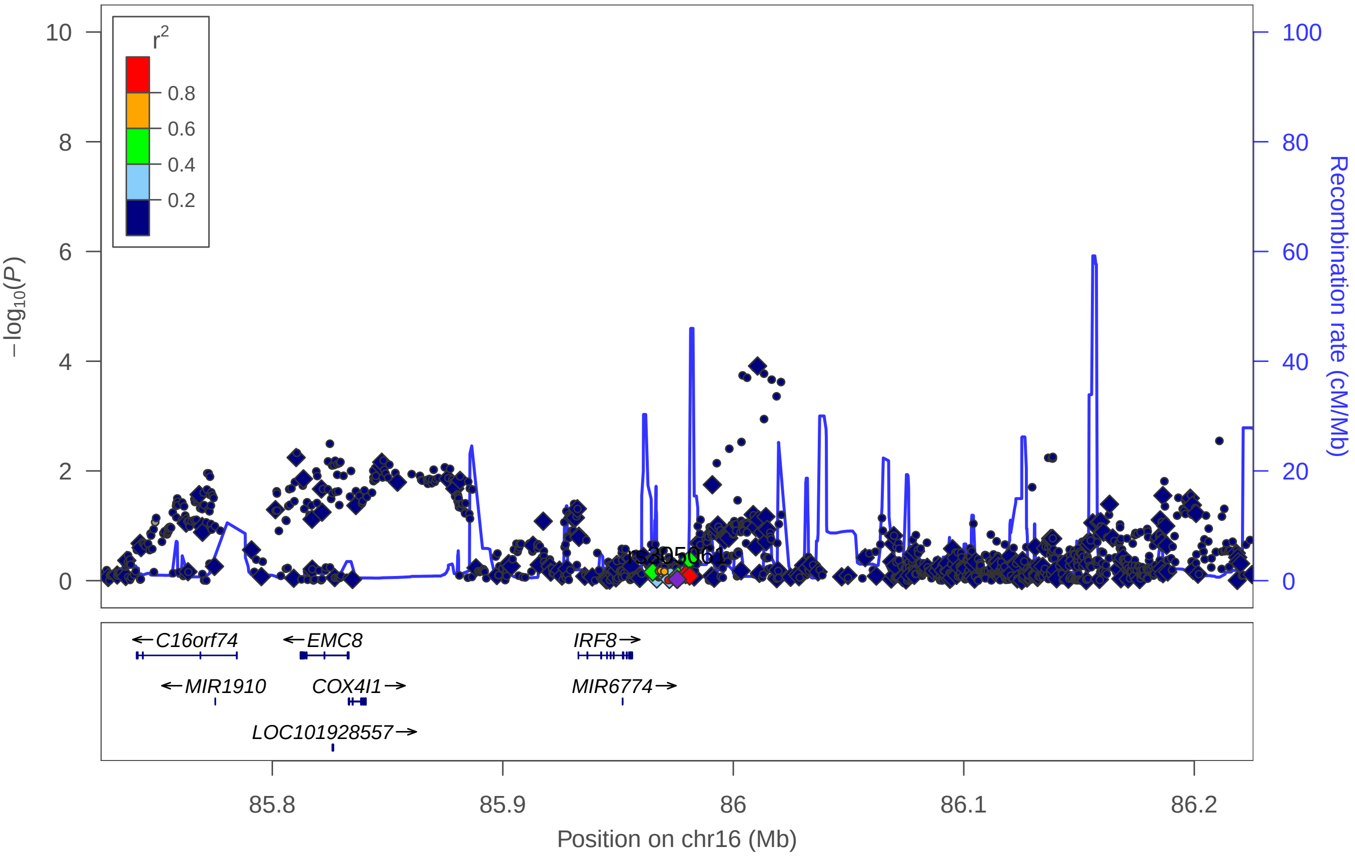

Supplementary Figure 53: **Regional association plot of TTFT (time to first time treatment) for known CLL etiological risk variant rs305061**. For each study, allelic dosage was estimated for the minor allele at each variant position and included in a Cox proportional hazard model to estimate hazard ratio (HR) and 95% confidence interval (CI). Variants were included in the meta-analysis if they had results from all six studies. Study-specific single nucleotide polymorphism (SNP) effects were combined in a fixed effect model using an inverse-variance-weighted method. The upper panel shows SNP coordinates based on genomic build b37/hg19 on the x-axis, and -log$_{10}$ (P values) on the y-axis. Genotyped and imputed SNPs are represented by diamonds and circles, respectively, and are coloured according to their linkage disequilibrium (pairwise r^2) with the CLL etiological risk variant based on the 1000 Genomes European panel. Reference genes in the region are shown in the lower panel, with arrows indicating transcript direction, dense blocks representing exons and horizontal lines representing introns.

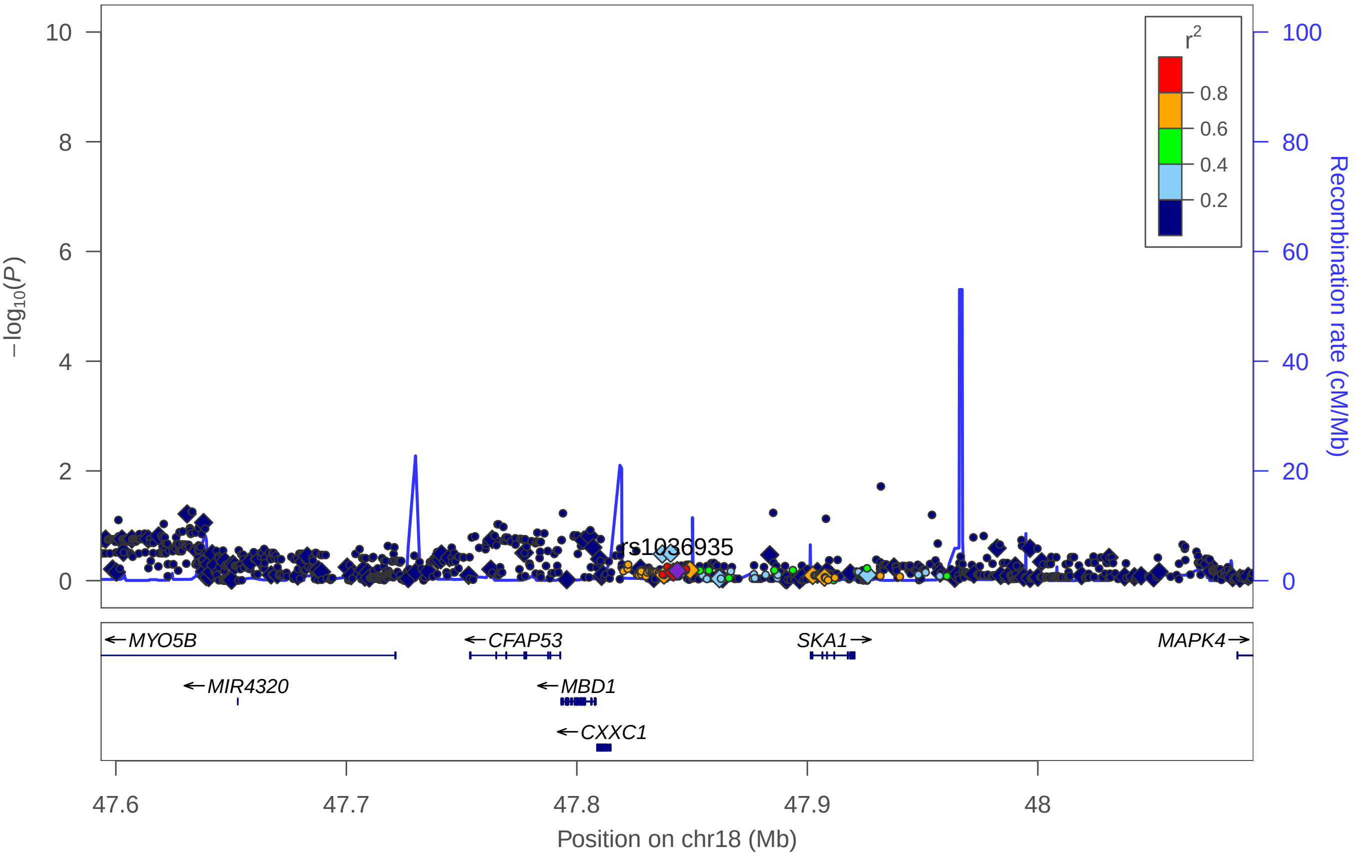

Supplementary Figure 54: **Regional association plot of TTFT (time to first time treatment) for known CLL etiological risk variant rs1036935**. For each study, allelic dosage was estimated for the minor allele at each variant position and included in a Cox proportional hazard model to estimate hazard ratio (HR) and 95% confidence interval (CI). Variants were included in the meta-analysis if they had results from all six studies. Study-specific single nucleotide polymorphism (SNP) effects were combined in a fixed effect model using an inverse-variance-weighted method. The upper panel shows SNP coordinates based on genomic build b37/hg19 on the x-axis, and -log$_{10}$ (P values) on the y-axis. Genotyped and imputed SNPs are represented by diamonds and circles, respectively, and are coloured according to their linkage disequilibrium (pairwise r^2) with the CLL etiological risk variant based on the 1000 Genomes European panel. Reference genes in the region are shown in the lower panel, with arrows indicating transcript direction, dense blocks representing exons and horizontal lines representing introns.

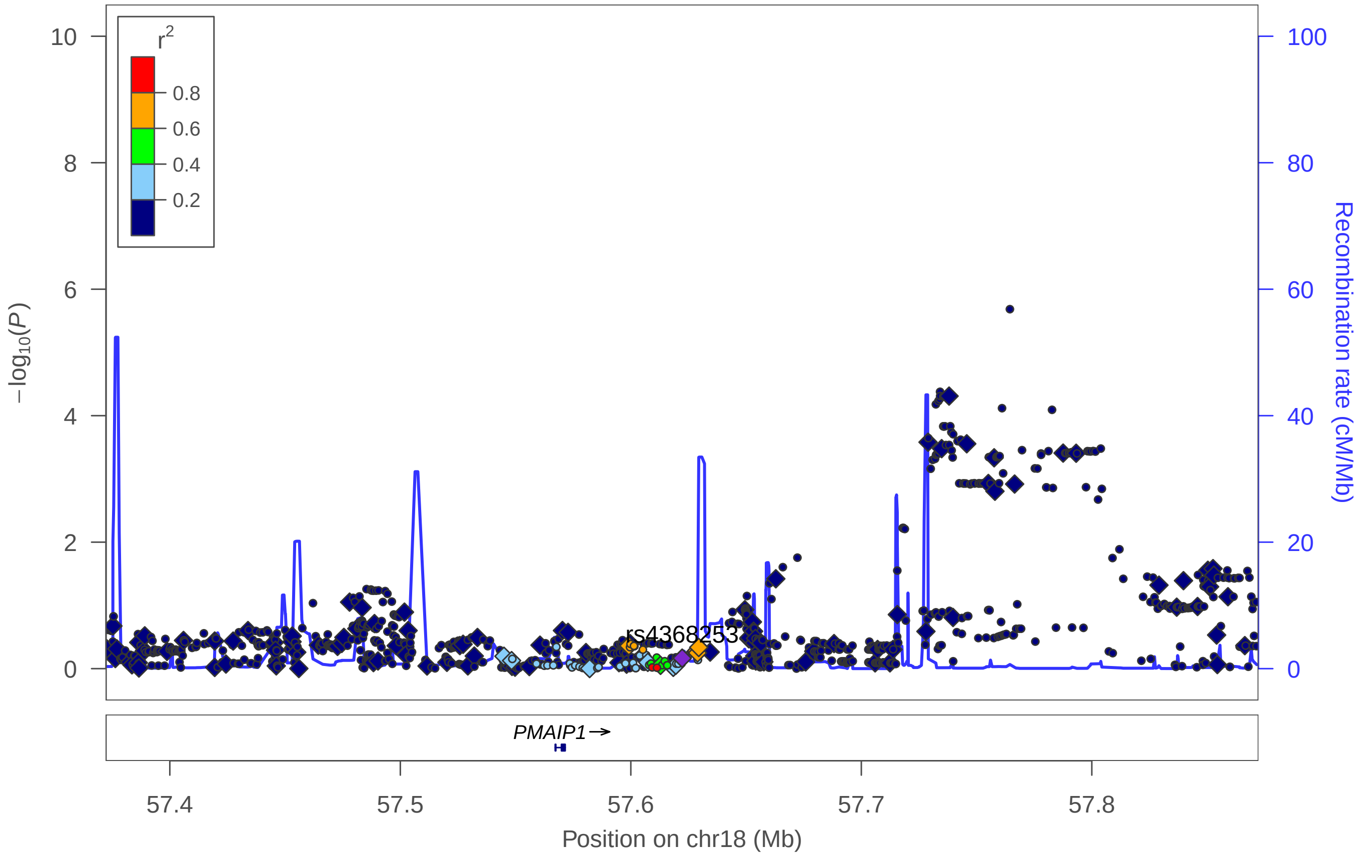

Supplementary Figure 55: **Regional association plot of TTFT (time to first time treatment) for known CLL etiological risk variant rs4368253**. For each study, allelic dosage was estimated for the minor allele at each variant position and included in a Cox proportional hazard model to estimate hazard ratio (HR) and 95% confidence interval (CI). Variants were included in the meta-analysis if they had results from all six studies. Study-specific single nucleotide polymorphism (SNP) effects were combined in a fixed effect model using an inverse-variance-weighted method. The upper panel shows SNP coordinates based on genomic build b37/hg19 on the x-axis, and -$\log_{10}$ (P values) on the y-axis. Genotyped and imputed SNPs are represented by diamonds and circles, respectively, and are coloured according to their linkage disequilibrium (pairwise r^2) with the CLL etiological risk variant based on the 1000 Genomes European panel. Reference genes in the region are shown in the lower panel, with arrows indicating transcript direction, dense blocks representing exons and horizontal lines representing introns.

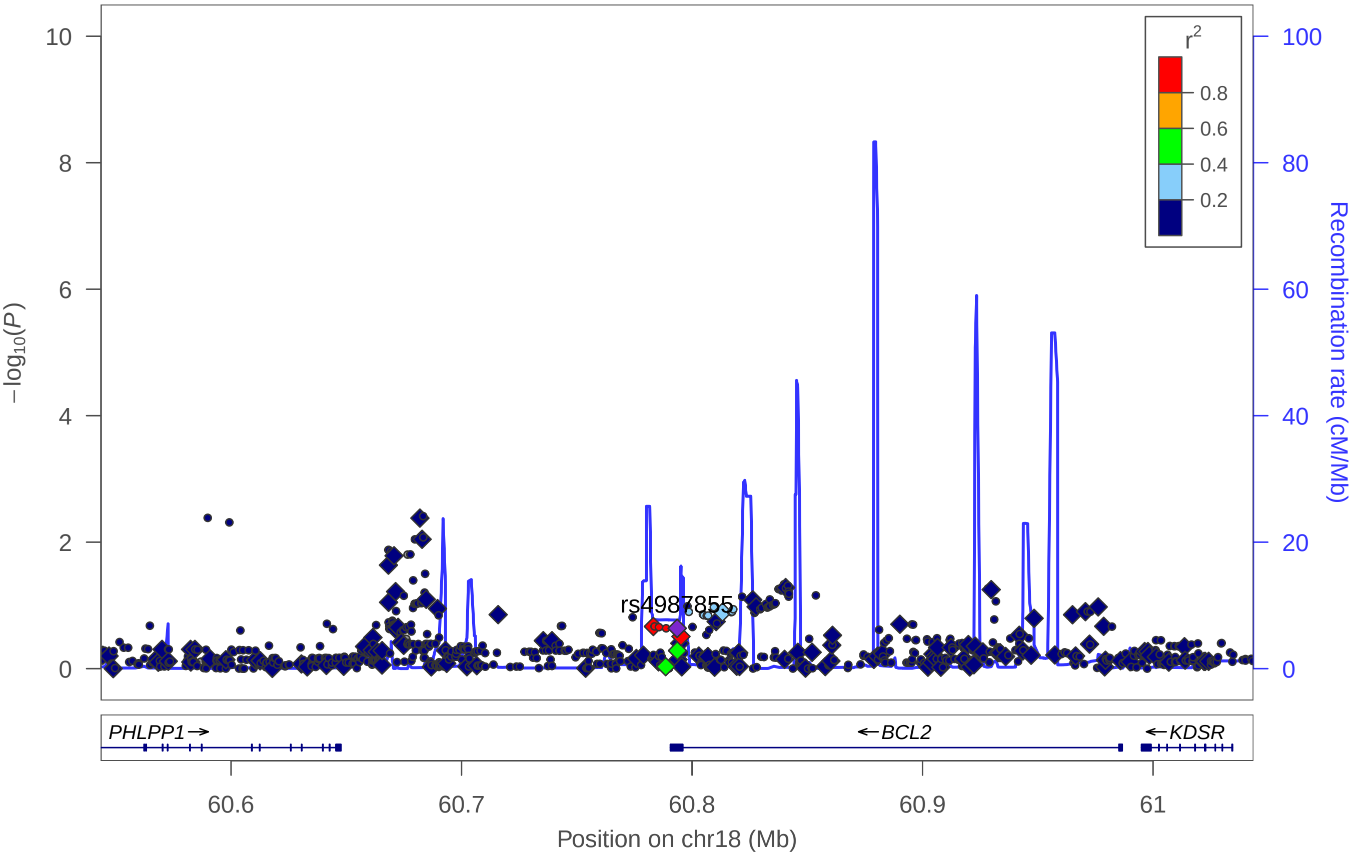

Supplementary Figure 56: **Regional association plot of TTFT (time to first time treatment) for known CLL etiological risk variant rs4987855**. For each study, allelic dosage was estimated for the minor allele at each variant position and included in a Cox proportional hazard model to estimate hazard ratio (HR) and 95% confidence interval (CI). Variants were included in the meta-analysis if they had results from all six studies. Study-specific single nucleotide polymorphism (SNP) effects were combined in a fixed effect model using an inverse-variance-weighted method. The upper panel shows SNP coordinates based on genomic build b37/hg19 on the x-axis, and -log$_{10}$ (P values) on the y-axis. Genotyped and imputed SNPs are represented by diamonds and circles, respectively, and are coloured according to their linkage disequilibrium (pairwise r^2) with the CLL etiological risk variant based on the 1000 Genomes European panel. Reference genes in the region are shown in the lower panel, with arrows indicating transcript direction, dense blocks representing exons and horizontal lines representing introns.

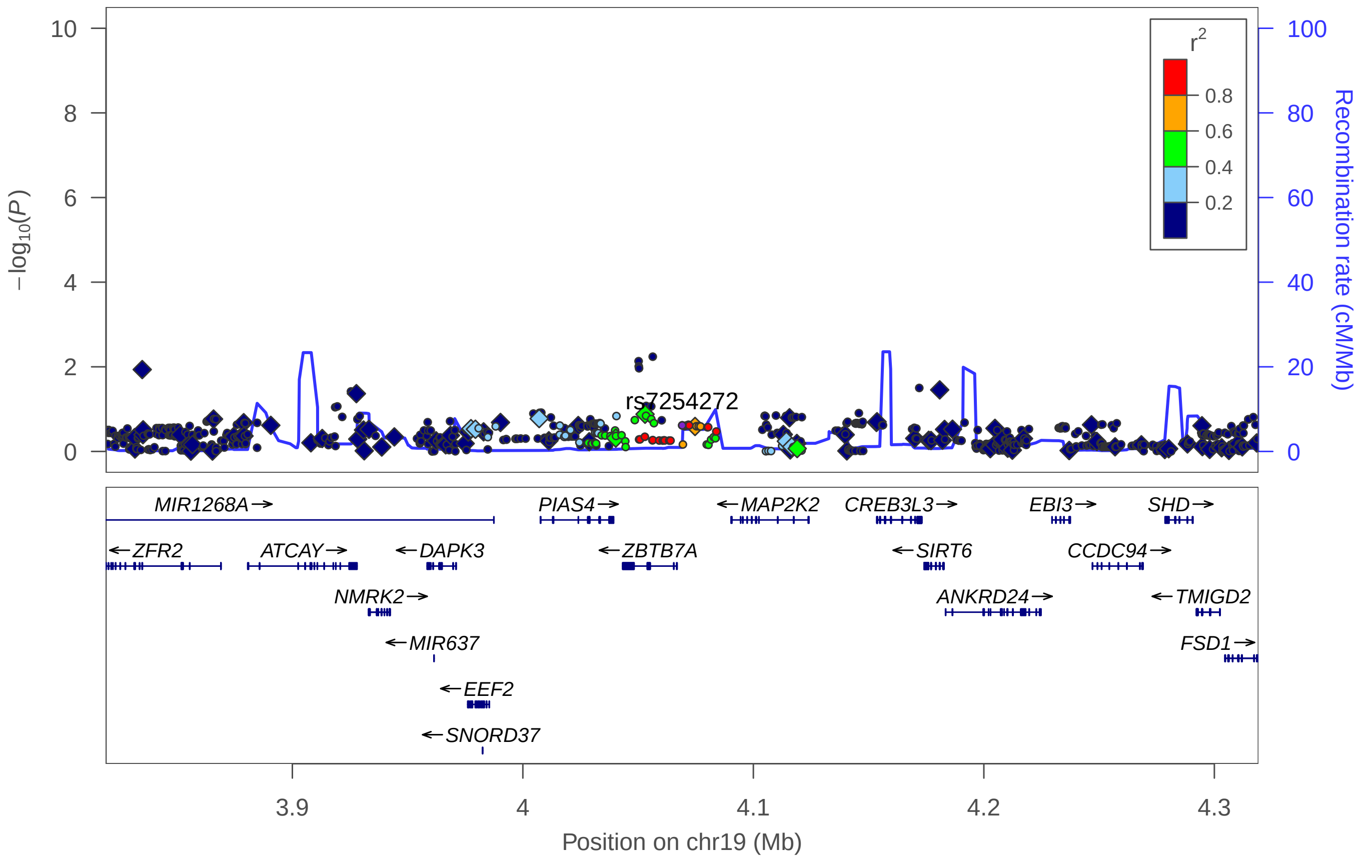

Supplementary Figure 57: **Regional association plot of TTFT (time to first time treatment) for known CLL etiological risk variant rs7254272**. For each study, allelic dosage was estimated for the minor allele at each variant position and included in a Cox proportional hazard model to estimate hazard ratio (HR) and 95% confidence interval (CI). Variants were included in the meta-analysis if they had results from all six studies. Study-specific single nucleotide polymorphism (SNP) effects were combined in a fixed effect model using an inverse-variance-weighted method. The upper panel shows SNP coordinates based on genomic build b37/hg19 on the x-axis, and -$\log_{10}$ (P values) on the y-axis. Genotyped and imputed SNPs are represented by diamonds and circles, respectively, and are coloured according to their linkage disequilibrium (pairwise r^2) with the CLL etiological risk variant based on the 1000 Genomes European panel. Reference genes in the region are shown in the lower panel, with arrows indicating transcript direction, dense blocks representing exons and horizontal lines representing introns.

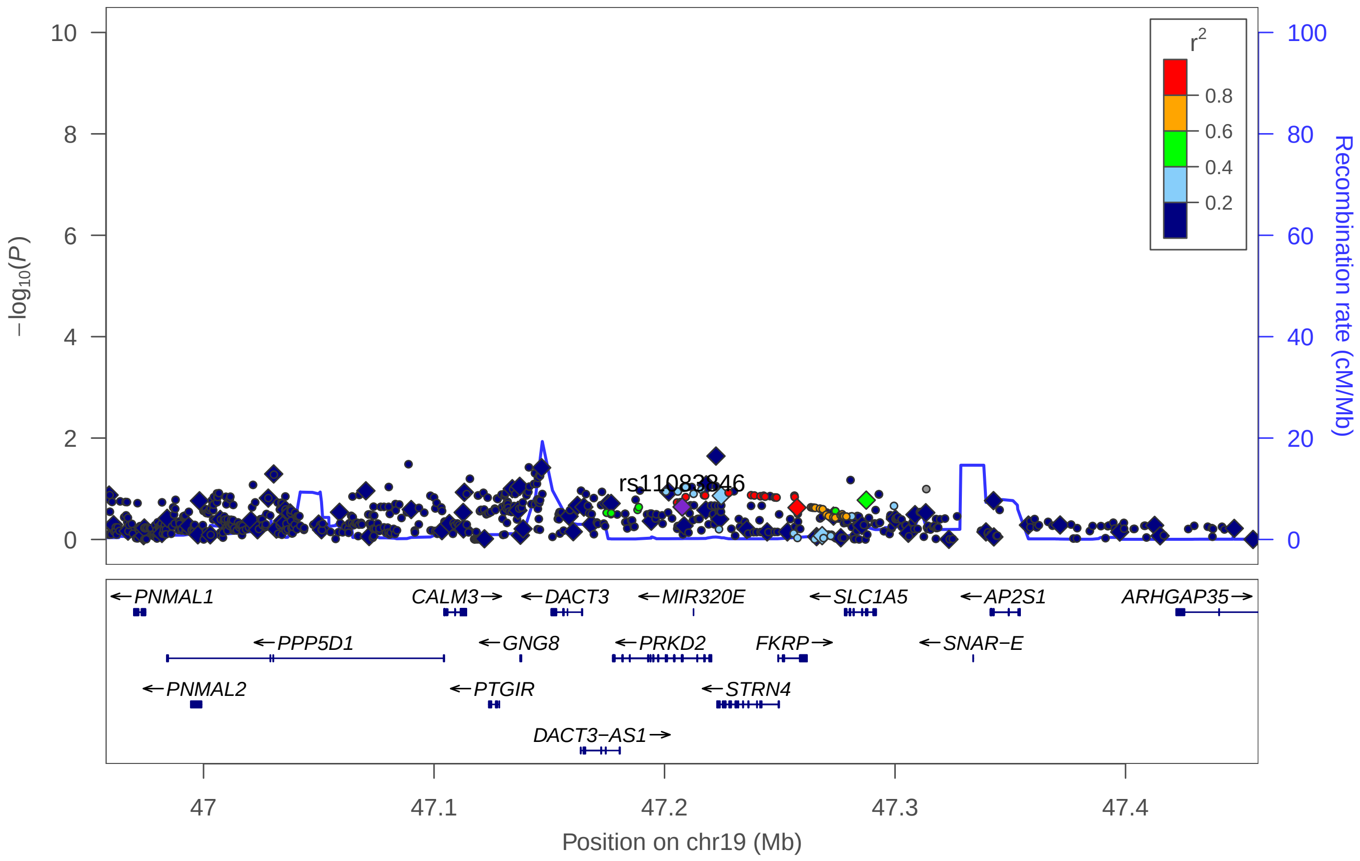

Supplementary Figure 58: **Regional association plot of TTFT (time to first time treatment) for known CLL etiological risk variant rs11083846**. For each study, allelic dosage was estimated for the minor allele at each variant position and included in a Cox proportional hazard model to estimate hazard ratio (HR) and 95% confidence interval (CI). Variants were included in the meta-analysis if they had results from all six studies. Study-specific single nucleotide polymorphism (SNP) effects were combined in a fixed effect model using an inverse-variance-weighted method. The upper panel shows SNP coordinates based on genomic build b37/hg19 on the x-axis, and -$\log_{10}$ (P values) on the y-axis. Genotyped and imputed SNPs are represented by diamonds and circles, respectively, and are coloured according to their linkage disequilibrium (pairwise r^2) with the CLL etiological risk variant based on the 1000 Genomes European panel. Reference genes in the region are shown in the lower panel, with arrows indicating transcript direction, dense blocks representing exons and horizontal lines representing introns.

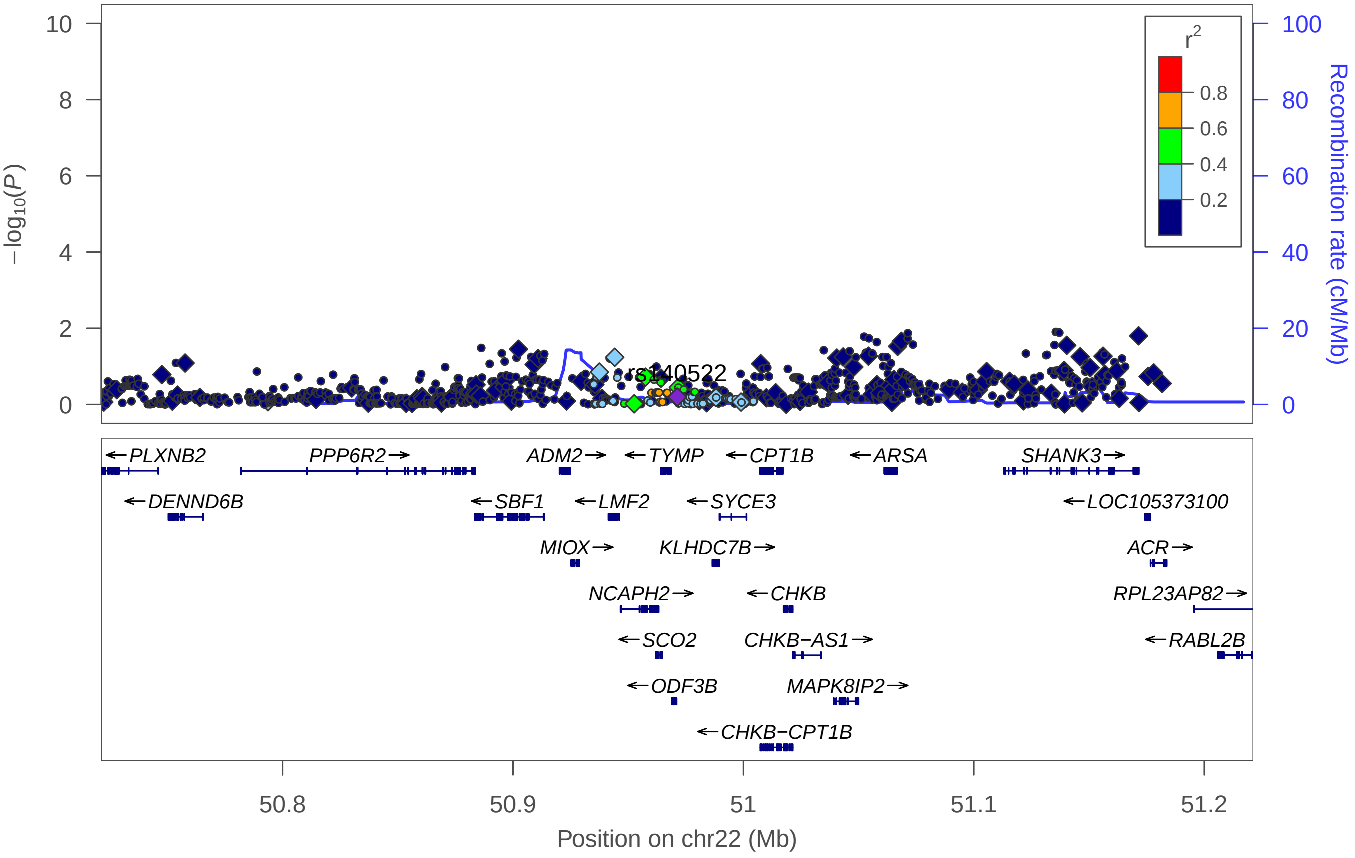

Supplementary Figure 59: **Regional association plot of TTFT (time to first time treatment) for known CLL etiological risk variant rs140522**. For each study, allelic dosage was estimated for the minor allele at each variant position and included in a Cox proportional hazard model to estimate hazard ratio (HR) and 95% confidence interval (CI). Variants were included in the meta-analysis if they had results from all six studies. Study-specific single nucleotide polymorphism (SNP) effects were combined in a fixed effect model using an inverse-variance-weighted method. The upper panel shows SNP coordinates based on genomic build b37/hg19 on the x-axis, and -$\log_{10}$ (P values) on the y-axis. Genotyped and imputed SNPs are represented by diamonds and circles, respectively, and are coloured according to their linkage disequilibrium (pairwise r^2) with the CLL etiological risk variant based on the 1000 Genomes European panel. Reference genes in the region are shown in the lower panel, with arrows indicating transcript direction, dense blocks representing exons and horizontal lines representing introns.

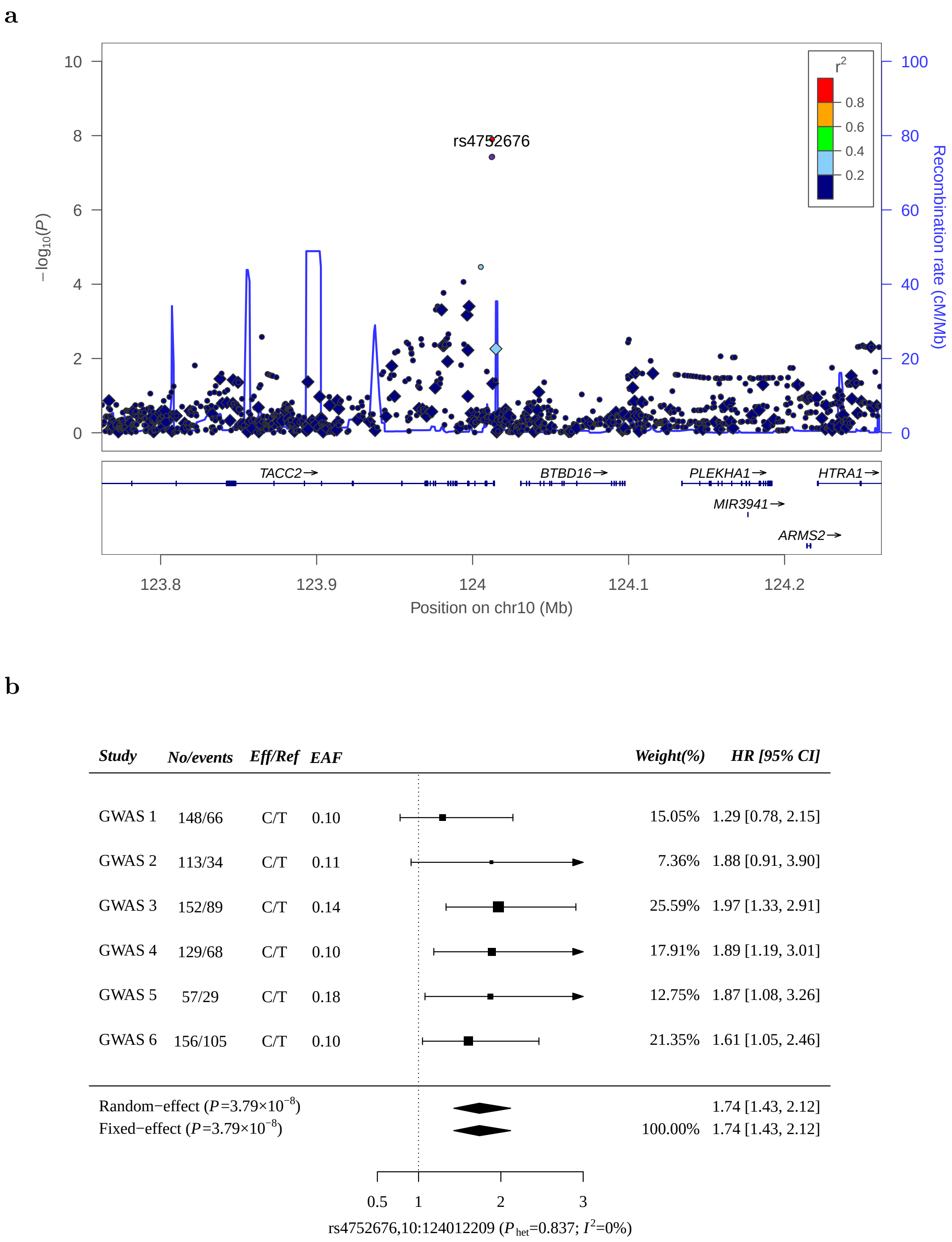

Supplementary Figure 60: **Regional and forest plots of rs4752676.** TTFT (time to first time treatment) survival associations in rs4752676 region (a). The upper panel plots SNPs based on genomic build b37/h19 coordinates on the x-axis, and their -$\log_{10}$(p-values) on the y-axis. Diamond and circle shapes are used for genotyped and imputed SNPs, respectively, with colours according to their pairwise r^2 with the lead SNP based on 1000 Genomes European panel (Nov 2014). Reference genes in the region are shown in the lower panel, with arrows indicating transcript direction, dense blocks for exons, and lines for introns. Plot was generated using LocusZoom. Forest plot of rs4752676 effect on TTFT by study (b). No/events: No. CLL patients/No. patients receiving treatment (the first time); Eff/Ref: effect/reference allele; EAF: frequencies of the effect allele; HR: hazard ratio; CI: confidence interval; Squares denote the per-allele HR, with their size inversely proportional to the variance of the effect estimates. Pooled HRs derived from both the fixed and random-effect models are shown in diamond shapes, with their corresponding meta p-values indicated in the left parentheses. Format of x-axis label is rsid, chromosome:position(b37), P for Cochran's Q test (P_{het}) and I^2 for heterogeneity in parenethesis. All statistical tests were two-sided.

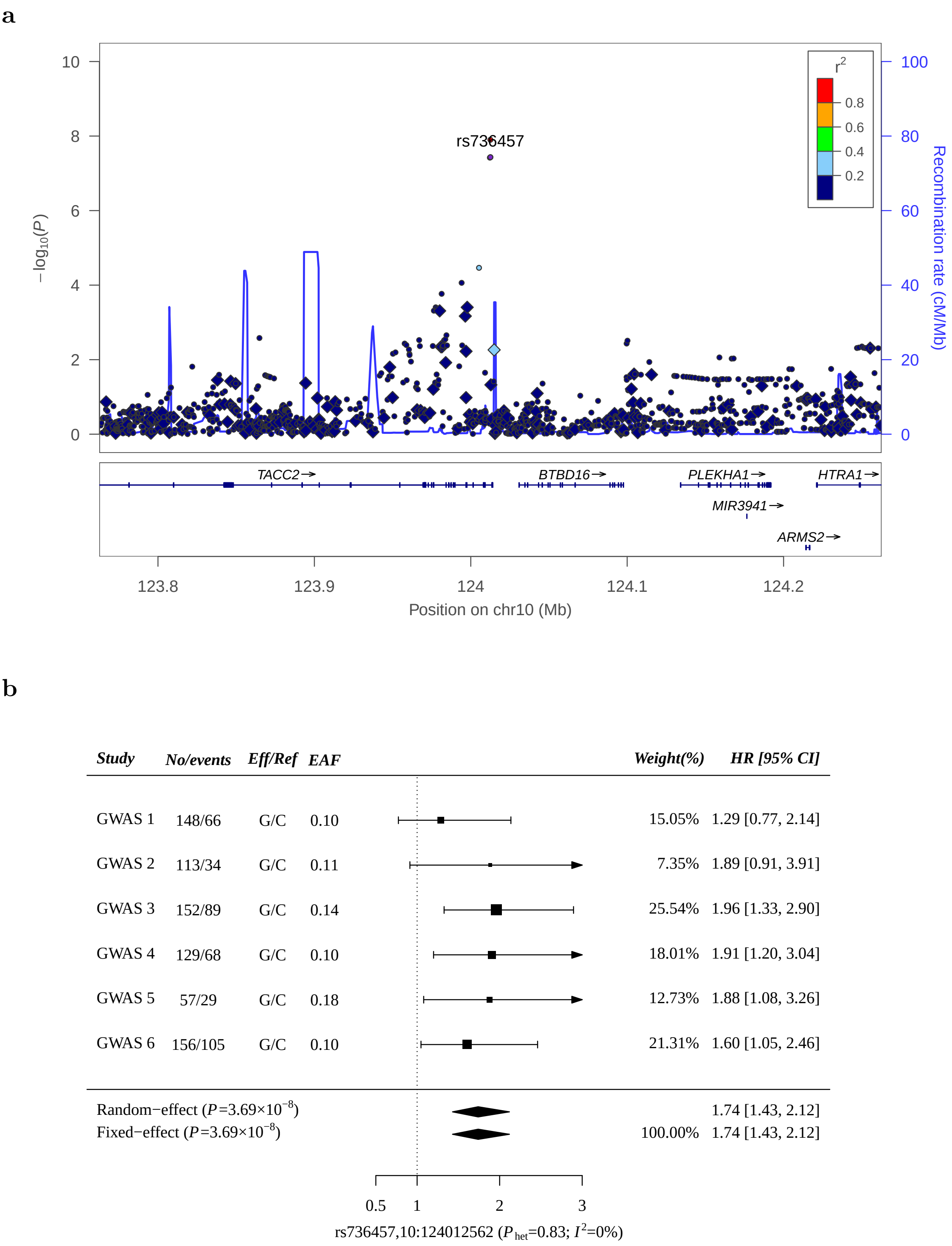

Supplementary Figure 61: **Regional and forest plots of rs736457.** TTFT (time to first time treatment) survival associations in rs736457 region (a). The upper panel plots SNPs based on genomic build b37/h19 coordinates on the x-axis, and their -$\log_{10}$(p-values) on the y-axis. Diamond and circle shapes are used for genotyped and imputed SNPs, respectively, with colours according to their pairwise r^2 with the lead SNP based on 1000 Genomes European panel (Nov 2014). Reference genes in the region are shown in the lower panel, with arrows indicating transcript direction, dense blocks for exons, and lines for introns. Plot was generated using LocusZoom. Forest plot of rs736457 effect on TTFT by study (b). No/events: No. CLL patients/No. patients receiving treatment (the first time); Eff/Ref: effect/reference allele; EAF: frequencies of the effect allele; HR: hazard ratio; CI: confidence interval; Squares denote the per-allele HR, with their size inversely proportional to the variance of the effect estimates. Pooled HRs derived from both the fixed and random-effect models are shown in diamond shapes, with their corresponding meta p-values indicated in the left parentheses. Format of x-axis label is rsid, chromosome:position(b37), P for Cochran's Q test (P_{het}) and I^2 for heterogeneity in parenethesis. All statistical tests were two-sided.

a

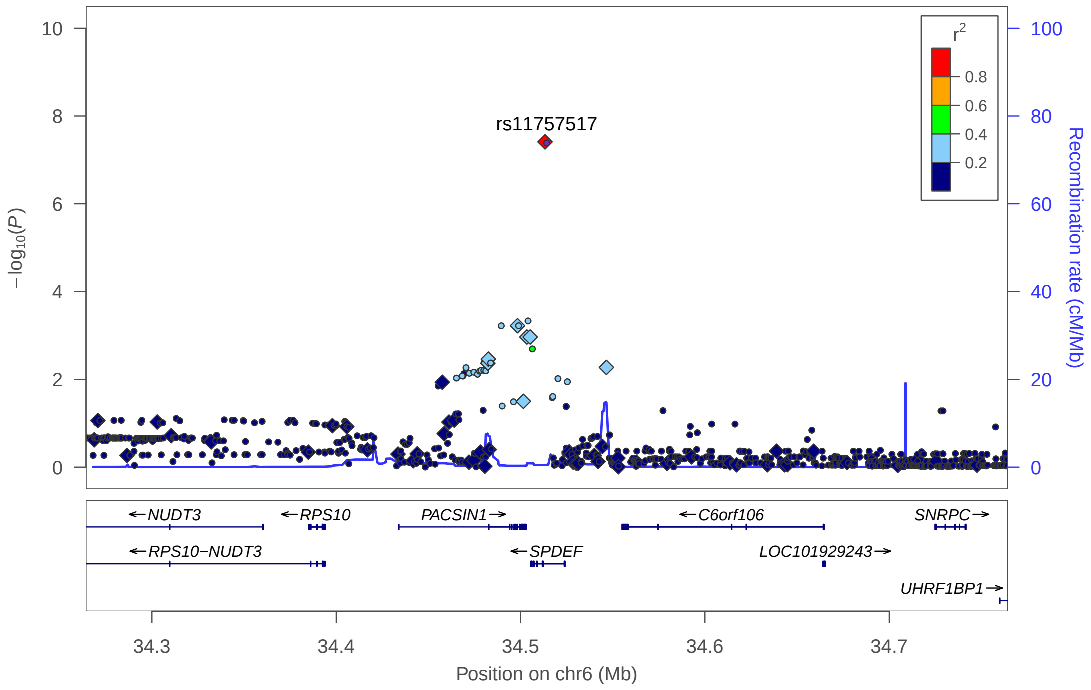

b

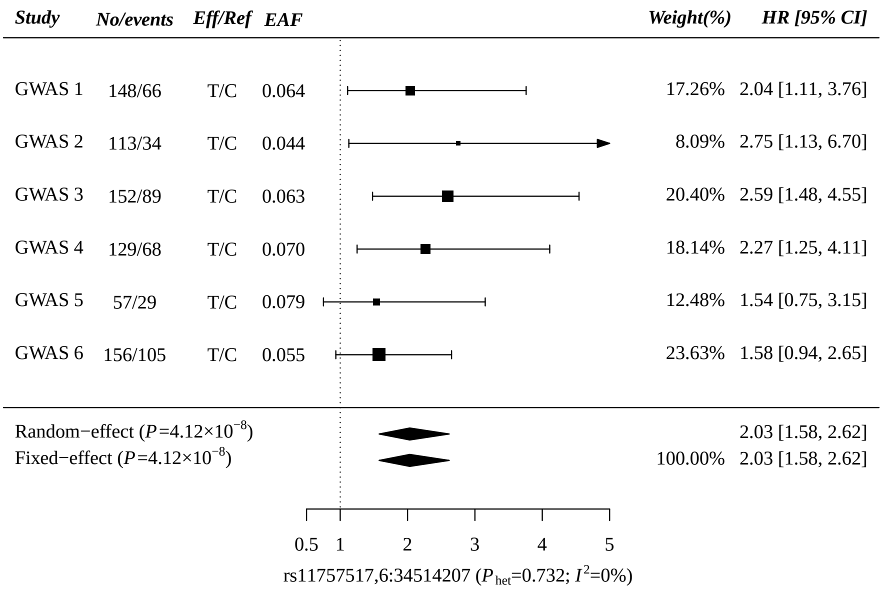

Supplementary Figure 62: **Regional and forest plots of rs11757517.** TTFT (time to first time treatment) survival associations in rs11757517 region (a). The upper panel plots SNPs based on genomic build b37/h19 coordinates on the x-axis, and their -$\log_{10}$(p-values) on the y-axis. Diamond and circle shapes are used for genotyped and imputed SNPs, respectively, with colours according to their pairwise r^2 with the lead SNP based on 1000 Genomes European panel (Nov 2014). Reference genes in the region are shown in the lower panel, with arrows indicating transcript direction, dense blocks for exons, and lines for introns. Plot was generated using LocusZoom. Forest plot of rs11757517 effect on TTFT by study (b). No/events: No. CLL patients/No. patients receiving treatment (the first time); Eff/Ref: effect/reference allele; EAF: frequencies of the effect allele; HR: hazard ratio; CI: confidence interval; Squares denote the per-allele HR, with their size inversely proportional to the variance of the effect estimates. Pooled HRs derived from both the fixed and random-effect models are shown in diamond shapes, with their corresponding meta p-values indicated in the left parentheses. Format of x-axis label is rsid, chromosome:position(b37), P for Cochran's Q test (P_{het}) and I^2 for heterogeneity in parenethesis. All statistical tests were two-sided.

Supplementary Table 1: Demographic and clinical characteristics of chronic lymphocytic leukemia (CLL) cases

		GWAS 1	GWAS 2	GWAS 3	GWAS 4	GWAS 5	GWAS 6	Study 7	*P* value*
Sex	Male	84	86	90	82	31	101	49	
	Female	69	37	62	47	26	55	38	0.128
	Not available	1	3	0	0	0	0	0	
Age	$\leq$ 65	70	49	87	64	32	56	36	
	>65	83	52	58	65	25	100	51	0.002
	Not available	1	25	7	0	0	0	0	
Binet stage	A	134	107	126	107	48	87	70	
	B/C	18	18	24	21	9	57	9	4.39×10^{-8}
	Not available	2	1	2	1	0	12	8	
IGHV	Mutated	110	97	94	31	1	63	0	
	Unmutated	43	26	50	10	0	46	2	0.003
	Not available	1	3	8	88	56	47	85	
CD38	Negative	98	74	95	89	33	49	54	
	Positive	52	50	29	29	23	28	29	0.023
	Not available	4	2	28	11	1	79	4	
β2 Microglobulin	$\leq$ 3.5 mgL^{-1}	0	40	83	75	0	0	2	
	>3.5mgL^{-1}	0	20	37	32	0	0	2	0.814
	Not available	154	66	32	22	57	156	83	
TP53 status	Normal	135	14	30	5	1	139	70	
	Abnormal	3	3	6	2	0	17	9	0.002
	Not available	16	109	116	122	56	0	8	

GWAS, genome-wide association study; *Fishers exact test. All statistical tests were two-sided.

Supplementary Table 2: eQTL results for rs736456

SNP ID	SNP position	Assessed Allele	Chromosome	Gene	Gene Symbol	Gene Position	*P* value eQTL[a]	P_{BH} eQTL[b]	Z score eQTL
rs736456	124012547	G	10	ENSG00000255624	RP11-564D11.3	124648738	0.07364703	0.3124953	1.7889
				ENSG00000179988	PSTK	124735463	0.08726587	0.3124953	-1.71
				ENSG00000107672	NSMCE4A	123725667	0.0961524	0.3124953	1.664
				ENSG00000095574	IKZF5	124759327	0.13754382	0.320929072	-1.4849
				ENSG00000154473	BUB3	124919339	0.14812111	0.320929072	1.4463
				ENSG00000066468	FGFR2	123297910	0.23855749	0.443035339	-1.1785
				ENSG00000107669	ATE1	123594127	0.41759542	0.562342092	0.8108
				ENSG00000166033	HTRA1	124247732	0.41771032	0.562342092	-0.8103
				ENSG00000119965	C10orf88	124702169	0.43257084	0.562342092	0.785
				ENSG00000196177	ACADSB	124793161	0.72288928	0.854323695	-0.3546
				ENSG00000138161	CUZD1	124615405	0.9349648	0.96378848	0.0818
				ENSG00000213185	FAM24B	124623875	0.96378848	0.96378848	0.0455
				ENSG00000107679	PLEKHA1	124163039	9.90E-17	1.28677E-15	8.3061

eQTL data for genes within 1MB of the risk SNP are shown. eQTL, expression quantitative trait loci; SNP, single nucleotide polymorphism.

[a]Unadjusted *P* value based on summary-data-based Mendelian randomization (SMR).

[b]Benjamini-Hochberg corrected *P* value.

Supplementary Table 3: eQTL results for rs3778076

SNP ID	SNP position	Assessed Allele	Chromosome	Gene	Gene Symbol	Gene Position	P value eQTL[a]	P_{BH} eQTL[b]	Z score eQTL
rs3778076	34513266	A	6	ENSG00000112039	FANCE	35427509	0.00083537	0.004646106	-3.3406
				ENSG00000124507	PACSIN1	34468461	0.00086039	0.004646106	-3.3326
				ENSG00000198755	RPL10A	35437373	0.01193704	0.05371668	-2.5139
				ENSG00000023892	DEF6	35277571	0.0367798	0.141864943	2.0884
				ENSG00000064999	ANKS1A	34958110	0.12283078	0.414553883	-1.5429
				ENSG00000269490	SBP1	33663335	0.14700276	0.44100828	-1.4501
				ENSG00000186577	C6orf1	34215702	0.22566553	0.609296931	-1.2115
				ENSG00000137309	HMGA1	34209329	0.25121634	0.616621925	-1.1473
				ENSG00000225339	RP11-513I15.6	34251000	0.30347967	0.667102562	1.029
				ENSG00000220583	RPL35P2	34231269	0.32119753	0.667102562	0.9922
				ENSG00000065029	ZNF76	35245224	0.35861887	0.691622106	-0.9179
				ENSG00000137288	MNF1	33672424	0.42347908	0.762262344	-0.8002
				ENSG00000146197	SCUBE3	35201523	0.47919369	0.779264312	0.7078
				ENSG00000196114	RP3-391O22.3	34544234	0.4981522	0.779264312	0.6775
				ENSG00000030110	BAK1	33544174	0.54664175	0.779264312	0.6029
				ENSG00000161904	LEMD2	33747946	0.61918905	0.779264312	-0.497
				ENSG00000112033	PPARD	35353151	0.65704258	0.779264312	-0.4439
				ENSG00000112664	NUDT3	34308224	0.663998	0.779264312	-0.4344
				ENSG00000124614	RPS10	34389566	0.67404719	0.779264312	-0.4205
				ENSG00000266509	MIR3934	33665958	0.69535379	0.779264312	0.3916
				ENSG00000096433	ITPR3	33626436	0.69683235	0.779264312	0.3896
				ENSG00000007866	TEAD3	35453113	0.72154103	0.779264312	-0.3562
				ENSG00000204188	GGNBP1	33554159	0.8424192	0.86674062	0.199
				ENSG00000064995	TAF11	34850710	0.86674062	0.86674062	0.1679
				ENSG00000065060	UHRF1BP1	34805386	2.49E-140	6.7281E-139	25.2188
				ENSG00000196821	C6orf106	34609850	2.62E-65	3.53943E-64	17.0668
				ENSG00000124562	SNRPC	34733377	7.57E-23	6.81399E-22	-9.8399

eQTL data for genes within 1MB of the risk SNP are shown. eQTL, expression quantitative trait loci; SNP, single nucleotide polymorphism.
[a]Unadjusted P value based on summary-data-based Mendelian randomization (SMR).
[b]Benjamini-Hochberg corrected P value.

Supplementary Table 4: eQTL results for rs3800461

SNP ID	SNP position	Assessed Allele	Chromosome	Gene	Gene Symbol	Gene Position	P value eQTL[a]	P_{BH} eQTL[b]	Z score eQTL
rs3800461	34616322	C	6	ENSG00000112039	FANCE	35427509	0.00100401	0.366527794	-3.2894
				ENSG00000124507	PACSIN1	34468461	0.00367018	0.003137531	-2.9052
				ENSG00000225339	RP11-513I15.6	34251000	0.00947011	0.010194944	2.5947
				ENSG00000065029	ZNF76	35245224	0.01256734	0.023675275	2.4959
				ENSG00000007866	TEAD3	35453113	0.0168943	0.028562136	2.389
				ENSG00000112664	NUDT3	34308224	0.0313344	0.035196458	2.1529
				ENSG00000186577	C6orf1	34215702	0.08235187	0.060258462	1.7372
				ENSG00000269490	SBP1	33663335	0.13823389	0.147056911	-1.4822
				ENSG00000064995	TAF11	34850710	0.21364682	0.230389817	-1.2436
				ENSG00000196114	RP3-391O22.3	34544234	0.2492389	0.333823156	1.1523
				ENSG00000137288	MNF1	33672424	0.27827613	0.386494625	-1.0842
				ENSG00000161904	LEMD2	33747946	0.33344406	0.438742184	-0.9672
				ENSG00000266509	MIR3934	33665958	0.4002359	0.481018595	0.8413
				ENSG00000146197	SCUBE3	35201523	0.40405562	0.481018595	-0.8342
				ENSG00000137309	HMGA1	34209329	0.55224461	0.627550693	-0.5943
				ENSG00000096433	ITPR3	33626436	0.60376074	0.656261674	0.519
				ENSG00000112033	PPARD	35353151	0.79162581	0.824610219	0.2644
				ENSG00000220583	RPL35P2	34231269	0.98165027	0.98165027	-0.0229
				ENSG00000124562	SNRPC	34733377	1.04E-60	8.66167E-60	-16.4368
				ENSG00000064999	ANKS1A	34958110	1.24E-06	0.00000516	-4.8493
				ENSG00000196821	C6orf106	34609850	3.27E-310	<1.00E-300	41.0453
				ENSG00000065060	UHRF1BP1	34805386	3.27E-310	<1.00E-300	51.2666
				ENSG00000023892	DEF6	35277571	4.10E-23	2.56556E-22	9.9014
				ENSG00000124614	RPS10	34389566	5.19E-05	0.000185439	-4.0468
				ENSG00000198755	RPL10A	35437373	5.31E-07	2.65445E-06	-5.0148

eQTL data for genes within 1MB of the risk SNP are shown. eQTL, expression quantitative trait loci; SNP, single nucleotide polymorphism.
[a]Unadjusted P value based on summary-data-based Mendelian randomization (SMR).
[b]Benjamini-Hochberg corrected P value.
