# Supplementary material for: Genome-wide association study identifies risk loci for progressive chronic lymphocytic leukemia
Source: Nat Commun. 2021 Jan 28;12:665. doi: 10.1038/s41467-020-20822-9 (PMC7843618; doi:10.1038/s41467-020-20822-9)
Supplement: Supplementary file 3 — Reporting Summary [file 41467_2020_20822_MOESM3_ESM.pdf]

nature research

Corresponding author(s): James Allan, David Allsup

Last updated by author(s): Nov 20, 2020

Reporting Summary

Nature Research wishes to improve the reproducibility of the work that we publish. This form provides structure for consistency and transparency in reporting. For further information on Nature Research policies, see Authors & Referees and the Editorial Policy Checklist.

Statistics

For all statistical analyses, confirm that the following items are present in the figure legend, table legend, main text, or Methods section.

n/a	Confirmed	
☐	☒	The exact sample size (*n*) for each experimental group/condition, given as a discrete number and unit of measurement
☒	☐	A statement on whether measurements were taken from distinct samples or whether the same sample was measured repeatedly
☐	☒	The statistical test(s) used AND whether they are one- or two-sided<br>*Only common tests should be described solely by name; describe more complex techniques in the Methods section.*
☐	☒	A description of all covariates tested
☐	☒	A description of any assumptions or corrections, such as tests of normality and adjustment for multiple comparisons
☐	☒	A full description of the statistical parameters including central tendency (e.g. means) or other basic estimates (e.g. regression coefficient) AND variation (e.g. standard deviation) or associated estimates of uncertainty (e.g. confidence intervals)
☐	☒	For null hypothesis testing, the test statistic (e.g. *F*, *t*, *r*) with confidence intervals, effect sizes, degrees of freedom and *P* value noted<br>*Give P values as exact values whenever suitable.*
☒	☐	For Bayesian analysis, information on the choice of priors and Markov chain Monte Carlo settings
☒	☐	For hierarchical and complex designs, identification of the appropriate level for tests and full reporting of outcomes
☒	☐	Estimates of effect sizes (e.g. Cohen's *d*, Pearson's *r*), indicating how they were calculated

Our web collection on statistics for biologists contains articles on many of the points above.

Software and code

Policy information about availability of computer code

Data collection	No software was used.
Data analysis	Genotypes were determined using Illumina GenomeStudio software (v2.0) (Illumina). Quality control and data analysis were performed using R and PLINK. B allele frequency and Log R ratio at variant positions was interrogated using Nexus Copy Number 10 software (build version 9665) (BioDiscovery, California). Haplotypes were estimated from genotypes using ShapeIT (v2.r790) implemented on the Michigan Imputation Server. Study-specific single nucleotide polymorphism (SNP) effects were combined using an inverse-variance-weighted method (fixed effects model) and the DerSimonian-Laird approach (random effects model) using R metafor package (v2.4-0). IGHV gene sequences were analysed using IgBlast (v1.3.0) (https://www.ncbi.nlm.nih.gov/igblast/). Regional plots were generated using LocusZoom downloaded from https://github.com/statgen/locuszoom-standalone.

For manuscripts utilizing custom algorithms or software that are central to the research but not yet described in published literature, software must be made available to editors/reviewers. We strongly encourage code deposition in a community repository (e.g. GitHub). See the Nature Research guidelines for submitting code & software for further information.

Data

Policy information about availability of data

All manuscripts must include a data availability statement. This statement should provide the following information, where applicable:

- Accession codes, unique identifiers, or web links for publicly available datasets
- A list of figures that have associated raw data
- A description of any restrictions on data availability

Genome-wide association summary statistics (Lin_TTFT_CLLmetaAssoc.txt) are available for download from 10.25405/data.ncl.12136365. eQTL data is available from the eQTLGen consortium via http://www.eqtlgen.org/cis-eqtls.html (2019-12-11-cis-eQTLsFDR-ProbeLevel-CohortInfoRemoved-BonferroniAdded.txt.gz). The source data underlying Figures 3A, 3C and Supplementary Figures 7, 10A, 10B, 11A, 11B, 60A, 61A and 62A are provided as a Source Data file. The remaining data are contained within the supplementary information files or available from the authors upon reasonable request.

Field-specific reporting

Please select the one below that is the best fit for your research. If you are not sure, read the appropriate sections before making your selection.

☒ Life sciences ☐ Behavioural & social sciences ☐ Ecological, evolutionary & environmental sciences

For a reference copy of the document with all sections, see nature.com/documents/nr-reporting-summary-flat.pdf

Life sciences study design

All studies must disclose on these points even when the disclosure is negative.

Sample size	Observational study, so the results are based on all available data from chronic lymphocytic leukemia patients recruited to participating centres. This study includes data on 842 new cases.
Data exclusions	Data were quality controlled using established measures. Specifically, for each GWAS we excluded SNP markers with departure from Hardy-Weinberg equilibrium (HWE; P ≤ 10-3), a call rate < 95% or with significant differences in minor allele frequency (P ≤ 10-3) between genotype batches. Samples were excluded if the call rate was < 95%, heterozygosity exceeded 3 standard deviations from the overall mean heterozygosity or were identified as non-European based on principal components analysis using 1000 genome data as a reference. Samples were also removed such that there were no two individuals with estimated relatedness pihat ≥ 0.1875, with retention of the sample with the higher call rate.
Replication	Observational study, so results based on all available data. Results were meta-analysed.
Randomization	Observational study, so randomisation not relevant. Sample recruitment based on a diagnosis of chronic lymphocytic leukemia.
Blinding	Observational study, so blinding not relevant. Sample recruitment based on a diagnosis of chronic lymphocytic leukemia.

Reporting for specific materials, systems and methods

We require information from authors about some types of materials, experimental systems and methods used in many studies. Here, indicate whether each material, system or method listed is relevant to your study. If you are not sure if a list item applies to your research, read the appropriate section before selecting a response.

Materials & experimental systems

n/a	Involved in the study	
☒	☐	Antibodies
☒	☐	Eukaryotic cell lines
☒	☐	Palaeontology
☒	☐	Animals and other organisms
☐	☒	Human research participants
☒	☐	Clinical data

Methods

n/a	Involved in the study	
☒	☐	ChIP-seq
☒	☐	Flow cytometry
☒	☐	MRI-based neuroimaging

Human research participants

Policy information about studies involving human research participants

Population characteristics	All patients had typical CLL with an absolute B cell count >5 x 109 L-1 expressing CD5, CD23, CD19 and were diagnosed in accordance with World Health Organisation guidelines contemporaneous to the time of diagnosis. Patient characteristics are described in the methods section and supplementary table 1 of the manuscript.
Recruitment	CLL patients were diagnosed at clinical centres in the United Kingdom, including Bournemouth (GWAS 1), Cardiff (GWAS 2), Hull (GWAS 3 and GWAS 4, study 7), Newcastle (GWAS 5) and Gateshead/Sunderland (GWAS 6). The results are based on all available data from chronic lymphocytic leukemia patients daignosed at participating centres.
Ethics oversight	Collection of patient samples and associated clinico-pathological information was undertaken with written informed consent. All studies were conducted in accordance with the Declaration of Helsinki and received local institutional review board and/or research ethics approval (Hull 08/H1304/35; Cardiff 02/4806). Newcastle, Gateshead and Sunderland CLL patient samples were collected and stored following approval from the Newcastle Academic Health Partners Biobank (http://www.ncl.ac.uk/biobanks/collections/nbrtb/).

Note that full information on the approval of the study protocol must also be provided in the manuscript.
